# σ- *versus* π-Activation of Alkynyl Benzoates Using B(C_6_F_5_)_3_

**DOI:** 10.3390/molecules20034530

**Published:** 2015-03-12

**Authors:** Alexander Bähr, Lewis C. Wilkins, Kevin Ollegott, Benson M. Kariuki, Rebecca L. Melen

**Affiliations:** School of Chemistry, Main Building, Cardiff University, Cardiff CF10 3AT, Cymru/Wales, UK; E-Mails: alexander.baehr@rub.de (A.B.); WilkinsLC@cardiff.ac.uk (L.C.W.); kevin.ollegott@rub.de (K.O.); KariukiB@cardiff.ac.uk (B.M.K.)

**Keywords:** boron, trispentafluorophenyl borane, B(C_6_F_5_)_3_, Lewis acid, alkyne

## Abstract

We have prepared a range of alkynyl benzoates in high yields and have investigated their reactivities with the strong Lewis acid B(C_6_F_5_)_3_. In such molecules both σ-activation of the carbonyl and π-activation of the alkyne are possible. In contrast to the reactivity of propargyl esters with B(C_6_F_5_)_3_ which proceed via 1,2-addition of the ester and B(C_6_F_5_)_3_ across the alkyne, the inclusion of an additional CH_2_ spacer switches off the intramolecular cyclization and selective σ-activation of the carbonyl group is observed through adduct formation. This change in reactivity appears due to the instability of the species which would be formed through B(C_6_F_5_)_3_ activation of the alkyne.

## 1. Introduction

The intrinsic Lewis acidity of Group 13 compounds has led to their broad application in Lewis acid- catalyzed reactions in synthetic chemistry. In particular, B(C_6_F_5_)_3_, which was first reported in the 1960s [1,2] has been widely employed in an assortment of transformations in both organic and organometallic chemistry [3,4,5,6,7,8,9,10,11,12]. Owing to its highly electrophilic but sterically protected nature, B(C_6_F_5_)_3_ has been commonly used as the Lewis acid component in Frustrated Lewis Pair (FLP) chemistry [13,14,15,16,17,18,19,20]. Combinations of a Lewis acid and a Lewis base that do not form an adduct constitute FLPs and have been widely used in a range of small molecule activation reactions and in metal free catalysis [13,14,15,16,17,18,19,20]. In our research we have focused on the activation of alkynes by electrophilic boron reagents towards nucleophilic attack. This reactivity is well-precedented in FLP chemistry and many examples have been reported in which the FLP adds in a 1,2-manner across the alkyne. Lewis bases in these reactions include bulky amines [21,22] phosphines [23,24,25,26] and pyrroles [27]. In the case of terminal alkynes deprotonation may occur, particularly in the case of more basic phosphines, e.g., P*^t^*Bu_3_, to yield phosphonium borate salts [R_3_PH][R′-C≡C-B(C_6_F_5_)_3_] [26,28,29,30,31,32]. B(C_6_F_5_)_3_ may also react with an alkyne in the absence of a nucleophile in a 1,1-carboboration reaction [33,34,35,36,37,38]. Such reactions have been used to generate intramolecular FLPs and complex boron containing compounds [39].

**Scheme 1:**
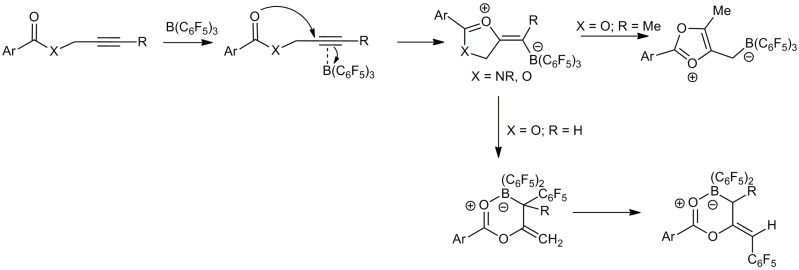
Cyclization pathways of propargyl amides and esters with the Lewis acid B(C_6_F_5_)_3_.

Previously we have probed how B(C_6_F_5_)_3_ can mimic established precious metal π-Lewis acid catalysts in intramolecular alkyne activation for the generation of oxazoles from propargyl amides [40] and formation of versatile boron allylation reagents from propargyl esters (Scheme 1) [41]. In all cases these intramolecular cyclization reactions involve the 1,2-addition of the carbonyl oxygen atom from the ester or amide and the borane across the alkyne [40,41,42]. Unlike the reactions of FLPs with alkynes, in these reactions the Lewis basic carbonyl oxygen atom is not sterically protected and thus coordination of the oxygen lone pairs to the borane is possible. This competitive activation process between the carbonyl and the alkyne is reflected in the rates of these cyclization reactions. For example, amide carbonyl groups coordinate better when compared to ester groups leading to slower cyclization as a result of poorer alkyne activation [43]. Conversely, propargyl esters undergo faster π-alkyne activation and hence faster 1,2-addition.

In this study we describe the synthesis of a range of alkynyl benzoates which include an additional methylene spacer between ester and alkynyl functionalities. We investigate their reactivity with B(C_6_F_5_)_3_ potentially affording access to ring-expanded derivatives of the established chemistry outlined in Scheme 1. Interestingly, π-activation appears to be entirely suppressed in favor of σ-adduct formation between the carbonyl group and the Lewis acid. Such differences in reactivity between these alkynyl benzoate substrates and the related propargyl esters and amides are discussed.

## 2. Results and Discussion

**Scheme 2:**
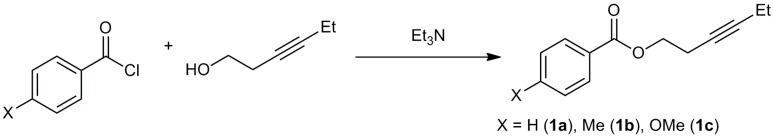
Synthesis of alkynyl benzoates.

A series of alkynyl benzoates **1a**–**c** were synthesized in moderate to high yields (72%–83%) from the room temperature reactions of hex-3-yn-1-ol with the corresponding benzoyl chloride derivatives in the presence of triethylamine as a weak base (Scheme 2). These compounds were fully characterized by multinuclear NMR, IR and mass spectroscopies.

**Figure 1:**
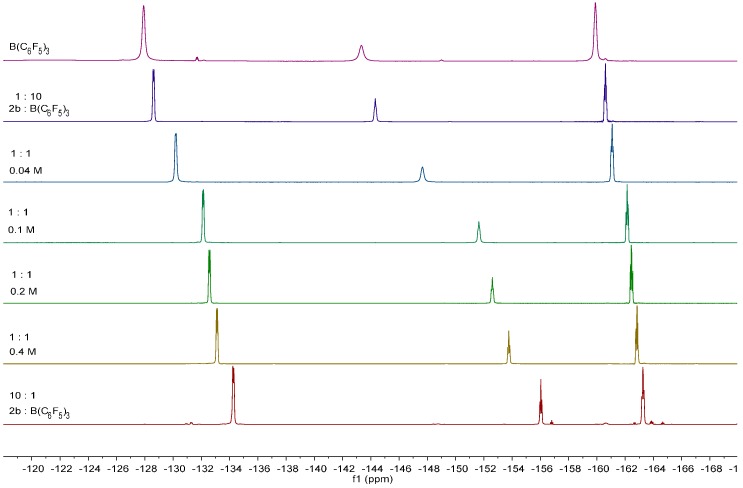
^19^F stacked spectra of the reactions of **1b** with B(C_6_F_5_)_3_.

Addition of the Lewis acid B(C_6_F_5_)_3_ to **1** at ambient temperature resulted in adduct formation between the ester oxygen atom and the vacant orbital at boron, evidenced by ^11^B-NMR data which displayed a broad peak consistent with other carbonyl adducts of B(C_6_F_5_)_3_ [43]. The ^11^B- and ^19^F-NMR spectra are dependent upon both the concentration of the reaction and on the mole ratio of B(C_6_F_5_)_3_ to alkynyl benzoate. With a large excess of B(C_6_F_5_)_3_ the ^19^F and ^11^B spectra correspond closely to that of free B(C_6_F_5_)_3_. Conversely with a large excess of ester, the peaks in both the ^19^F- and ^11^B-NMR spectra shift to high field. In the ^11^B-NMR spectrum the signal is broad and its chemical shift is consistent with adduct formation. These observations are consistent with an equilibrium whose dynamics are rapid on the NMR timescale. These are supported by concentration dependent measurements which show an upfield shift in the ^11^B-NMR spectrum with increasing concentration whose chemical shifts are close to that with excess alkynyl benzoate. At a low concentration (0.04 M) the positions correspond closely to the reactions with a ten-fold excess of B(C_6_F_5_)_3_ and that of free B(C_6_F_5_)_3_ (Figure 1). 

**Scheme 3:**
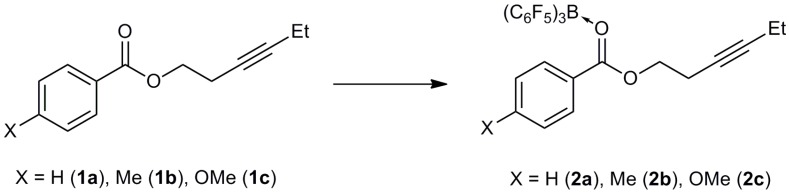
Formation of adducts from the reactions of alkynyl benzoates with B(C_6_F_5_)_3_.

The 1:1 stoichiometric reactions of alkynyl benzoates with B(C_6_F_5_)_3_ on a 0.2 mmol scale followed by recrystallization resulted in the formation of the ester-B(C_6_F_5_)_3_ adducts **2a**–**c** (Scheme 3) which were characterized by X-ray diffraction (*vide infra*). The IR spectra of the adducts **2** all show a red-shift in the carbonyl stretching frequency relative to the alkynyl benzoates **1** of *ca.* 70 cm^−1^ upon coordination to boron (Table 1). 

**Table 1:** IR stretching frequencies for free and coordinated alkynyl benzoates. 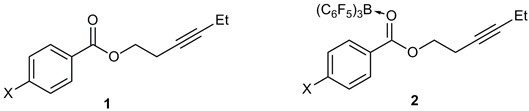

X	IR Carbonyl Stretching Frequency (cm^−1^)		
	*ν*_CO_ (1)	*ν*_CO_ (2)	Δ*ν*_CO_
H (**a**)	1717	1647	70
Me (**b**)	1717	1647	70
OMe (**c**)	1713	1645	68

### 2.1. Crystallographic Studies

**Figure 2:**
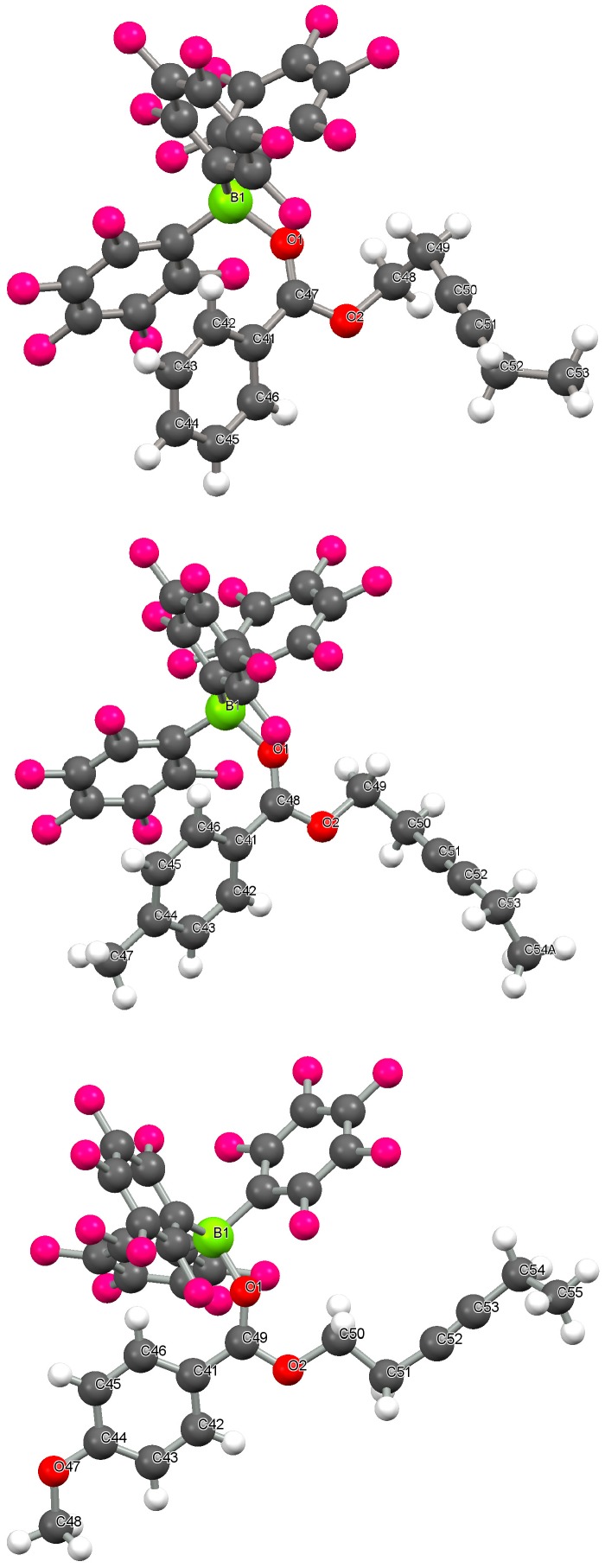
Crystal structure of **2a** (top), **2b** (middle) and **2c** (bottom). C: grey, O: red, H: white, B: yellow-green, F: pink.

Large colorless crystals of **2a**–**c** suitable for X-ray diffraction could be obtained by cooling a very concentrated hot toluene/petroleum ether solution. The solvent could then be decanted off and the crystals washed to give analytically pure **2a** and **2b** in 36%–38% recovered yield, whilst **2c** was recovered in 26% yield. The adducts **2a**–**c** all crystallized in the triclinic *P-1* space group with one molecule in the asymmetric unit (Figure 2). Compounds **2a** and **2b** show very similar B(1)-O(1) bond lengths [1.589(2) Å and 1.585(2)Å (2a and 2b respectively)] and are identical within error (Table 2). However, the B(1)-O(1) bond length in **2c** is shorter at 1.565(2) Å. These distances are all similar to B-O bond lengths observed previously; a search of the CSD (2013) revealed 2822 structures containing a B-O bond between 4-coordinate boron and 2-coordinate oxygen with a mean B-O distance of 1.48(4) Å. The C=O bond lengths in **2** are 1.247(2) Å (**2a**), 1.255(2) Å (**2b**), 1.255(2) Å (**2c**) and are just slightly longer than the mean C=O bond distances for conventional ester compounds reported on the CSD (52047 structures, 2013) at 1.20 Å. In all three cases, the adducts adopt a bent geometry from the donation of the HOMO of the carbonyl (one of the lone pairs in an *sp^2^* orbital on the oxygen atom) with the borane coordinated in a formally *cis* conformation to the aryl group with respect to the C=O. The C(1)-O(1)-B(1) angles in **2a**–**c** are 135.5(1)°, 135.8(1)° and 138.4(1)° respectively. The C(2)-C(1)-O(1)-B(1) dihedral angle in **2a**–**c** are 23.18°, 29.62° and 33.80° respectively with the boron atom lying out of the C(2)-C(1)-O(1) plane. This presumably arises due to steric interactions between the aryl group on the alkynyl benzoates and the perfluoroaryl groups on boron since a dihedral angle of 0° would be expected to be the most favorable energetically [43]. In all cases the aryl ring on the alkynyl benzoates is rotated slightly such that there is reduced conjugation with the carbonyl group with C(3)-C(2)-C(1)-O(1) dihedral angles of 32.54° (**2a**), 31.12° (**2b**) and 13.02° (**2c**). The distortions are very similar for **2a** and **2b** although this distortion for **2c** is much less suggesting a greater extent of conjugation presumably brought about by the electron donating ability of the *para*-oxygen atom. This is also reflected in a slightly shorter C(2)-C(1) bond between the aryl ring and the carbonyl group of 1.451(2) Å (**2c**) in relation to the same C(2)-C(1) bond length in **2a** [1.474(2) Å].

**Table 2:** Structural properties of **2a**–**c**. 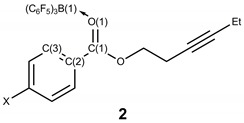

	Compound		
	2a	2b	2c
B(1)-O(1)/Å	1.589(2)	1.585(2)	1.565(2)
C(1)-O(1)/Å	1.247(2)	1.255(2)	1.255(2)
C(2)-C(1) bond length/Å	1.474(2)	1.462(2)	1.451(2)
C(1)-O(1)-B(1) angle/°	135.5(1)	135.8(1)	138.4(1)
C(2)-C(1)-O(1)-B(1) dihedral angle/°	23.18	29.62	33.80
C(3)-C(2)-C(1)-O(1) dihedral angle/°	32.54	31.12	13.02

### 2.2. Computational Studies

The electron-donating abilities of the aryl ring increase in the order Ph < *p*-MeC_6_H_4_ < *p*-MeOC_6_H_4_ based on their Hammett parameters (0.000, −0.170 and −0.268 respectively) [44]. These appear in agreement with the B-O bond lengths which show a shortening with increasing donor ability. However, the variation in the C-O bond lengths and particularly the change in *ν*_CO_ are more ambiguous and prompted us to undertake theoretical calculations to probe this behavior. DFT studies were undertaken to determine the optimized structures (B3LYP/6-31G*) and thermodynamic calculations were determined using the higher level triple zeta 6-311G* basis set. Calculations were undertaken on the esters **1**, B(C_6_F_5_)_3_ and the corresponding adducts **2**. The B-O and C-O bond lengths in the geometry-optimized structures and the energetics of adduct formation (corrected for ZPE) are presented in Table 3. These clearly support the general geometric changes reflected in the crystallographic and IR data that adduct formation occurs with concomitant weakening of the C=O bond with the computed energetics correlating well with those expected based on the Hammett parameter. The apparent anomalous behavior in the IR spectra of **2c** is not manifested in these calculations and may arise as a feature of the solid state packing (in relation to gas phase computations). The slightly smaller shift in Δ*ν*_CO_ (2 cm^−1^) corresponds to just 0.02 kJ/mol and some slight weakening of this interaction could easily be absorbed to accommodate crystal packing forces. In this context it is notable that the torsion associated with the aryl-carboxyl fragment is substantially smaller for **2c** than **2a** and **2b**. 

**Table 3:** B3LYP/6-31G* geometry-optimized B-O and C-O bond lengths determined for **1** and **2** along with energetics of adduct formation based on B3LYP/6-311G* calculations.

Ester	C=O/Å	Adduct	C-O/Å	B-O/Å	∆H_adduct_/kJ·mol^−1^
**1a**	1.21	**2a**	1.25	1.61	−8
**1b**	1.21	**2b**	1.25	1.60	−14
**1c**	1.21	**2c**	1.25	1.60	−17

The enthalpy of adduct formation in all cases is small when compared to a classical B-O covalent bond (*ca.* 530 kJ/mol) [45] but is consistent with the significant steric demands of the B(C_6_F_5_)_3_ group. These enthalpy changes indicate that this is likely a reversible process as is experimentally observed for adducts of propargyl amides and esters [40,41,42]. Indeed the Gibbs free energy changes for adduct formation are all positive, in agreement with such a supposition. 

### 2.3. Effect of Temperature

**Scheme 4:**
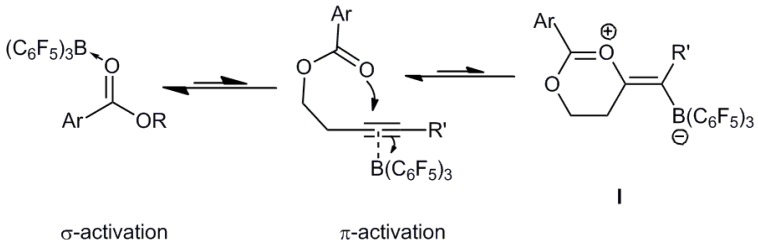
Adduct formation *versus* 1,2-addition.

**Scheme 5:**
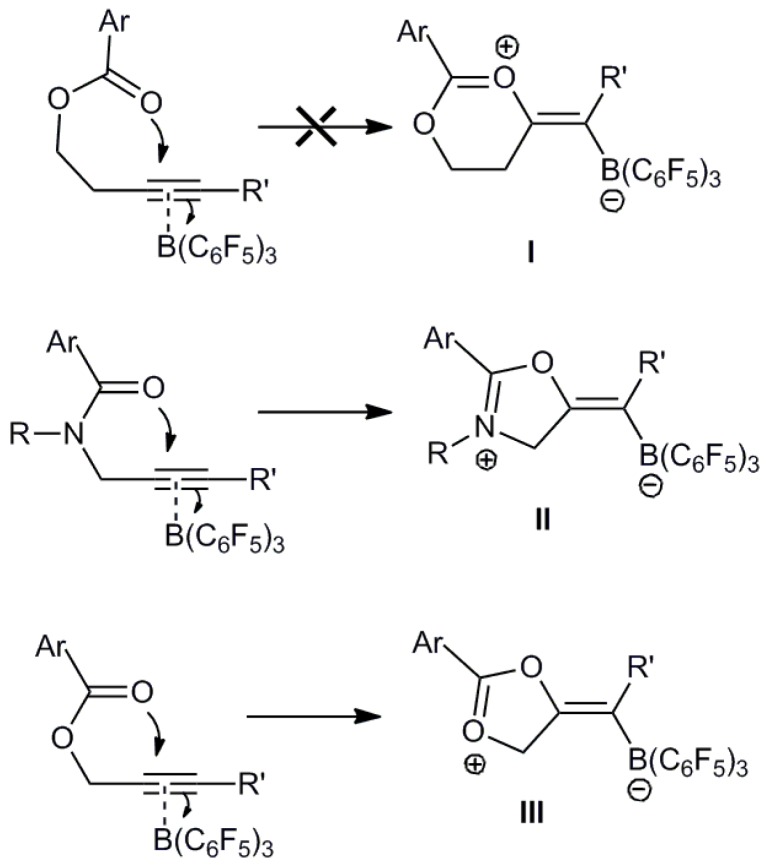
1,2-Addition products.

We subsequently investigated if these compounds would undergo 1,2-addition to form the zwitterionic 1,2-addition products similar to those seen previously with the reactions of propargyl esters and amides with B(C_6_F_5_)_3_ [40,41,42]. In both those cases the initial adduct could be driven to dissociate and, at elevated temperatures, undergo 1,2-addition at the alkyne. In contrast to the propargyl esters and amides, even after extended heating these reactions showed no significant sign of 1,2-addition products. Although, in the *in situ* ^11^B-NMR reactions of **1** with B(C_6_F_5_)_3_ a sharp signal of extremely low intensity at −17.0 ppm (**1a**) and at −17.1 ppm (**1c**) could be observed after 4 days at 45 °C. This sharp signal is typical for four coordinate borate species indicating possible B−C bond formation. This chemical shift is similar to that observed for the cyclization of propargyl esters with B(C_6_F_5_)_3_ which gave rise to a chemical shift at −17.1 ppm [41]. At elevated temperatures there is no doubt that the initial adduct will be in equilibrium with the free acid B(C_6_F_5_)_3_ and ester in solution, the lack of reactivity of the alkyne is therefore unexpected and presumably arises from some instability in the initial six-membered ring product formed by cyclization. In this context we considered the mechanistic process in more detail (Scheme 4). Previously it was suggested that sterically demanding propargyl amides may undergo reversible 1,2-addition [40]. We therefore attribute the lack of significant amounts of 1,2-addition product to the instability of the carbocation in the zwitterionic product (**I**). In previous studies, propargyl amides undergo 1,2-addition to afford stable zwitterionic 5-alkylidene-4,5-dihydrooxazolium borate compounds [40] (**II**) in which the positive charge is localized predominantly on the amide nitrogen atom which exhibits better stabilization of positive charge over oxygen. In the case of the isolobal propargyl esters the 1,2-addition product was also observed to give **III**. However, this was found to be unstable and to rearrange rapidly with ring opening in solution to give allyl boron compounds (Scheme 5) [41]. This rapid rearrangement was attributed to the instability of the carbocation formed in the 1,2-addition product and also supports the instability of the 1,2-addition product, **I**. In addition formation of 6-membered rings is somewhat less favorable than 5-membered rings and so the additional methylene group in **1** compared to the propargyl esters also mitigates the propensity ring closure. 

## 3. Experimental Section 

### 3.1. General Information

With the exception of the synthesis of starting materials, all reactions including storage of the starting materials, room temperature reactions, product recovery and sample preparation for analysis were carried out under a dry, O_2_-free atmosphere using a nitrogen-filled glove box (MBRAUN, Garching, Germany). Molecular sieves (4 Å) were dried at 150 °C for 48 h prior to use. Toluene and DCM solvents were dried by employing a Grubbs-type column system (MBRAUN), degassed and stored over molecular sieves under a nitrogen atmosphere. Petroleum ether (bp. 40–60 °C) was distilled and stored over molecular sieves. Deuterated CDCl_3_ was dried over molecular sieves before use. Chemicals were purchased from commercial suppliers and used as received. ^1^H, ^13^C and ^11^B and spectra were recorded on Avance DPX-500 or 400 spectrometers (Bruker, Billerica, MA, USA). ^19^F-NMR were recorded on a JEOL Eclipse 300 spectrometer (Peabody, MA, USA). Chemical shifts are expressed as parts per million (ppm, δ) downfield of tetramethylsilane (TMS) (δ = 0 ppm) and are referenced to CDCl_3_ as internal standards. NMR spectra were referenced to CFCl_3_ (^19^F) and BF_3_•Et_2_O/CDCl_3_ (^11^B). All coupling constants are absolute values and *J* values are expressed in Hertz (Hz). Mass spectral data were performed in house employing electrospray ionization techniques in positive ion mode. Infrared spectra were recorded on an IRAffinity^−1^ FT-IR spectrometer (Shimadzu, Kyot, Japan). Infrared data are quoted in wavenumbers (cm^−1^). Elemental analysis results were determined by Mr. Stephen Boyer using the elemental analysis service at London Metropolitan University, U.K.

### 3.2. Synthesis of Starting Materials

#### 3.2.1. Synthesis of Hex-3-yn-1-yl benzoate (**1a**)

To DCM (100 mL), triethylamine (TEA, 14 mL, 100 mmol) and benzyl chloride (5.8 mL, 50 mol) were added at 273 K. 3‑Hexyn‑1‑ol (5.5 mL, 50 mmol,) was then added slowly to this solution. The reaction was stirred overnight at 298 K. The resulting solution was then washed with water and brine and the solvent was removed to give a dark yellow oil. The oil was cooled to −50 °C to give a solid which was then washed with cold hexane to give pure **1a**. Yield: 8.27 g, 41 mmol, 82%. IR (wavenumbers in cm^−1^): 2978, 2938, 2361, 1717, 1603, 1584, 1452, 1385, 1316, 1267, 1109, 1069, 1026, 708. ^1^H-NMR (500 MHz, CDCl_3_, 298 K): 8.03 (m, 2H, *o*-ArH), 7.52 (tt, 1H, ^3^*J*_HH_ = 7.8 Hz, ^4^*J*_HH_ = 1.1 Hz, *p*-ArH), 7.40 (t, 2H, ^3^*J*_HH_ = 7.9 Hz, *m*-ArH), 4.35 (t, 2H, ^3^*J*_HH_ = 7.0 Hz, -CH_2_O(CO)-), 2.60 (m, 2H, ‑C≡CCH_2_‑CH_2_-), 2.13 (m, 2H, Me-CH_2_-C≡), 1.08 (t, 3H, ^3^*J*_HH_ = 7.4 Hz, -CH_3_). ^13^C-NMR (125 MHz, CDCl_3_, 298 K): 166.3, 132.9, 130.2, 129.6, 128.3, 83.5, 74.9, 63.3, 19.4, 14.1, 12.4. MS (ES^+^, *m*/*z*): 202.10 (M+), 123.03, 105.69, 80.05, 77.02, 76.03, 65.04. 

#### 3.2.2. Synthesis of Hex-3-yn-1-yl 4-methylbenzoate (**1b**)

To DCM (100 mL), TEA (14 mL, 100 mmol) and *p*-tolyl chloride (8.0 mL, 60 mmol) were added at 273 K. 3‑Hexyn‑1‑ol (5.5 mL, 50 mmol,) was then slowly added. The reaction was stirred overnight at 298 K. The solution was then washed with water and brine and the solvent removed to give a yellow oil. The oil was purified by column chromatography using a mixture of hexane and ethyl acetate (80/20 vol. %) to give pure **1b**. Yield: 7.82 g, 36 mmol, 72%. IR (wavenumbers in cm^−1^): 2972, 2940, 2367, 1717, 1613, 1578, 1508, 1454, 1385, 1310, 1270, 1177, 1105, 1020, 752. ^1^H-NMR (400 MHz, CDCl_3_, 298 K): 7.94 (d, 2H, ^3^*J*_HH_ = 8.4 Hz, *o*-ArH), 7.23 (d, 2H, ^3^*J*_HH_ = 8.4 Hz, *m*-ArH), 4.36 (t, 2H, ^3^*J*_HH_ = 7.1 Hz, -CH_2_O(CO)-), 2.61 (tt, 2H, ^3^*J*_HH_ = 7.1 Hz, ^4^*J*_HH_ = 2.4 Hz, ‑C≡CCH_2_‑CH_2_-), 2.40 (s, 3H, -CH_3_), 2.15 (qt, 2H, ^3^*J*_HH_ = 7.5 Hz, ^4^*J*_HH_ = 2.4 Hz, Me-CH_2_-C≡), 1.11 (t, 3H, ^3^*J*_HH_ = 7.5 Hz, -CH_3_). ^13^C-NMR (101 MHz, CDCl_3_, 298 K): 166.5, 143.7, 129.8, 129.1, 127.5, 83.6, 80.0, 63.2, 21.8, 19.5, 14.2, 12.5. MS (ES^+^, *m*/*z*): 216.115 (M^+^), 137.04, 119.75, 91.04, 79.04, 77.04, 65.03.

#### 3.2.3. Synthesis of Hex-3-yn-1-yl 4-methoxybenzoate (**1c**)

To DCM (100 mL), TEA (14 mL, 100 mmol) and 6.8 mL 4‑methoxybenzylchloride (50 mmol) were added at 273 K. 3‑Hexyn‑1‑ol (5.5 mL, 50 mmol) was then added slowly. The reaction was subsequently stirred overnight at 298 K. The resulting solution was washed with water and brine and the solvent removed to give a brown solid which was washed with cold hexane to give pure **1c**. Yield: 9.62 g, 41 mmol, 83%. Melting point: 42 °C. IR (wavenumbers in cm^−1^): 2974, 2363, 1780, 1713, 1605, 1578, 1512, 1387, 1317, 1256, 1167, 1101, 1028, 843, 768. ^1^H-NMR (500 MHz, CDCl_3_, 298 K): 8.00 (d, 2H, ^3^*J*_HH_ = 8.9 Hz, *o*-ArH), 6.91 (d, 2H, ^3^*J*_HH_ = 8.9 Hz, *m*-ArH), 4.34 (t, 2H, ^3^*J*_HH_ = 7.1 Hz, -CH_2_O(CO)-), 3.85 (s, 3H, -OCH_3_), 2.60 (tt, 2H, ^3^*J*_HH_ = 7.1 Hz, ^4^*J*_HH_ = 2.3 Hz, ‑C≡CCH_2_‑CH_2_-), 2.15 (qt, 2H, ^3^*J*_HH_ = 7.5 Hz, ^4^*J*_HH_ = 2.3 Hz, Me-CH_2_-C≡), 1.11 (t, 3H, ^3^*J*_HH_ = 7.5 Hz, -CH_3_). ^13^C-NMR (125 MHz, CDCl_3_, 298 K): 166.2, 163.5, 131.8, 122.8, 113.7, 83.6, 75.1, 63.2, 55.6, 19.6, 14.3, 12.5. MS (ES^+^, *m*/*z*): 232.11 (M^+^), 152.02, 135.02, 107.05, 92.02, 80.06, 79.05, 77.03, 65.04, 64.03. 

#### 3.2.4. Synthesis of Trispentafluorophenylborane, B(C_6_F_5_)_3_

Trispentafluorophenylborane [B(C_6_F_5_)_3_] was synthesized in a manner similar to that reported previously [46]. Magnesium turnings (7.2 g, 0.3 mol) were suspended in ether (*ca.* 600 mL) and a small amount of iodine added followed by the addition of a little BrC_6_F_5_ (74.1 g, 0.3 mol) dropwise resulting in a turbid grey mixture. Once the Grignard reaction had initiated, the remaining BrC_6_F_5_ was added slowly whilst making sure the solution does not reflux by cooling the reaction on an ice bath when necessary. Once the addition of BrC_6_F_5_ was complete, the resulting mixture was stirred for 1h at room temperature giving a dark brown/black solution. The solution was then cooled to 0 °C and transferred to a cooled solution of BF_3_·OEt_2_ (14.19 g, 0.1 mol) in toluene (*ca.* 200 mL). The resulting solution was allowed to warm to room temperature and the majority of the ether solvent was removed *in vacuo*. The resulting solution was then heated to 95 °C for 1h and the remaining solvent removed to give a brown solid. The solid was extracted with hot petroleum ether (500 mL) and the solution cooled to −80 °C to result in crystallization of B(C_6_F_5_)_3_. The solid was extracted three further times using the same solvent from the recrystallization mixture. The solvent was then filtered off from the B(C_6_F_5_)_3_ and the product dried under vacuum. ^19^F-NMR (376 MHz, CDCl_3_, 298 K): −127.89 (br. s, 2F, *o*-F), −143.32 (br. s, 1F, *p*-F), −159.91 (m, 2F, *m*-F).

### 3.3. Synthesis of Adducts

#### 3.3.1. Synthesis of **2a**

Compound **1a** (40 mg, 0.2 mmol) was dissolved in toluene (5 mL) and was added to B(C_5_F_6_)_3_ (105 mg, 0.2 mmol). The solution was left overnight and the solvent was removed and the remaining brown oil was recrystallized from a concentrated solution of pet. ether (40–60) and DCM. The crystals were washed with pet. ether (3 × 2 mL) to afford the pure product **2a**. Yield: 51 mg, 0.07 mmol, 36%. Melting point: 119 °C. IR (wavenumbers in cm^−1^): 3420, 3171, 2336, 1647, 1591, 1570, 1458, 1285, 1235, 1103, 980, 719. ^1^H-NMR (500 MHz, CDCl_3_, 298 K) *crystals*: 8.03 (m, 2H, *o*-ArH), 7.57 (tt, 1H, ^3^*J*_HH_ = 7.4 Hz, ^4^*J*_HH_ = 1.3 Hz, *p*-ArH), 7.44 (br. t, 2H, ^3^*J*_HH_ = 7.8 Hz, *m*-ArH), 4.37 (t, 2H, ^3^*J*_HH_ = 7.1 Hz, -CH_2_O(CO)-), 2.62 (tt, 2H, ^3^*J*_HH_ = 7.1 Hz, ^4^*J*_HH_ = 2.4 Hz, ‑C≡CCH_2_‑CH_2_-), 2.16 (qt, 2H, ^3^*J*_HH_ = 7.5 Hz, ^4^*J*_HH_ = 2.5 Hz, Me-CH_2_-C≡), 1.11 (t, 3H, ^3^*J*_HH_ = 7.5 Hz, -CH_3_). *In situ* ^13^C-NMR (125 MHz, CDCl_3_, 298 K, 0.2 M): 170.4 (s), 148.0 (m, C_6_F_5_), 141.8 (m, C_6_F_5_), 137.4 (m, C_6_F_5_), 134.2 (s), 130.2 (s), 128.7 (s), 128.5 (s), 115.8 (m, C_6_F_5_), 84.3 (s), 74.7 (s), 66.1 (s), 19.4 (s), 14.1 (s), 12.5 (s). ^19^F-NMR (376 MHz, CDCl_3_, 298 K) *crystals*: −128.26 (br. s, 2F, *o*-F), −143.71 (br. s, 1F, *p*-F), −160.01 (m, 2F, *m*-F). Elemental analysis calcd (%) for C_31_H_14_BF_15_O_2_·2DCM: C 44.83, H 2.05; Obs. C 44.73, H 1.78.

#### 3.3.2. Synthesis of **2b**

Compound **1b** (43 mg, 0.2 mmol) was dissolved in toluene (5 mL) and was added to B(C_5_F_6_)_3_ (105 mg, 0.2 mmol). The solution was left overnight and the solvent was removed and the remaining brown oil was recrystallized from a concentrated solution of pet. ether (40–60) and DCM. The crystals were washed with pet. ether (3 × 2 mL) to afford the pure product **2b**. Yield: 55 mg, 0.08 mmol, 38%. Melting point: 130 °C. IR (wavenumbers in cm^−1^): 3217, 2916, 2346, 1647, 1587, 1559, 1518, 1464, 1285, 1105, 1086, 970. ^1^H-NMR (500 MHz, CDCl_3_, 298 K) *crystals*: 7.86 (d, 2H, ^3^*J*_HH_ = 8.1 Hz, *o*-ArH), 7.21 (d, 2H, ^3^*J*_HH_ = 8.1 Hz, *m*-ArH), 4.44 (t, 2H, ^3^*J*_HH_ = 6.9 Hz, -CH_2_O(CO)-), 2.64 (tt, 2H, ^3^*J*_HH_ = 7.0 Hz, ^4^*J*_HH_ = 2.4 Hz, ‑C≡CCH_2_‑CH_2_-), 2.40 (s, 3H, -CH_3_), 2.15 (qt, 2H, ^3^*J*_HH_ = 7.5 Hz, ^4^*J*_HH_ = 2.4 Hz, Me-CH_2_-C≡), 1.11 (t, 3H, ^3^*J*_HH_ = 7.5 Hz, -CH_3_). *In situ* ^13^C-NMR (125 MHz, CDCl_3_, 298 K, 0.2 M): 171.1 (s), 147.9 (m, C_6_F_5_), 145.9 (s), 145.5 (s), 141.0 (m, C_6_F_5_), 137.3 (m, C_6_F_5_), 130.5 (s), 129.2 (s), 116.5 (m, C_6_F_5_), 84.3 (s), 74.0 (s), 66.4 (s), 21.8 (s), 19.4 (s), 14.1 (s), 12.4 (s). ^19^F-NMR (376 MHz, CDCl_3_, 298 K) *crystals*: −130.23 (br. s, 2F, *o*-F), −147.73 (br. s, 1F, *p*-F), −160.06 (m, 2F, *m*-F). Elemental analysis calcd (%) for C_32_H_16_BF_15_O_2_: C 52.78, H 1.98; Obs. C 52.65, H 1.73.

#### 3.3.3. Synthesis of **2c**

Compound **1c** (46 mg, 0.2 mmol) was dissolved in toluene (5 mL) and was added to B(C_5_F_6_)_3_ (105 mg, 0.2 mmol). The solution was left overnight and the solvent was removed and the remaining brown oil was recrystallized from a concentrated solution of pet. ether (40–60) and DCM. The crystals were washed with pet. ether (3 × 2 mL) to afford the pure product **2c**. Yield: 39 mg, 0.05 mmol, 26%. Melting point: 118 °C. IR (wavenumbers in cm^−1^): 3165, 2955, 2355, 1645, 1605, 1557, 1516, 1454, 1379, 1277, 1177, 1099, 1026, 974, 770. ^1^H-NMR (500 MHz, CDCl_3_, 298 K) *crystals*: 7.95 (d, 2H, ^3^*J*_HH_ = 9.0 Hz, *o*-ArH), 6.89 (d, 2H, ^3^*J*_HH_ = 9.0 Hz, *m*-ArH), 4.41 (t, 2H, ^3^*J*_HH_ = 7.0 Hz, -CH_2_O(CO)-), 3.86 (s, 3H, -OCH_3_), 2.63 (tt, 2H, ^3^*J*_HH_ = 7.0 Hz, ^4^*J*_HH_ = 2.3 Hz, ‑C≡CCH_2_‑CH_2_-), 2.16 (qt, 2H, ^3^*J*_HH_ = 7.6 Hz, ^4^*J*_HH_ = 2.3 Hz, Me-CH_2_-C≡), 1.11 (t, 3H, ^3^*J*_HH_ = 7.6 Hz, -CH_3_). *In situ* ^13^C-NMR (125 MHz, CDCl_3_, 298 K, 0.2 M): 171.6 (s), 165.2 (s), 147.8 (m, C_6_F_5_), 141.2 (m, C_6_F_5_), 137.1 (m, C_6_F_5_), 133.2 (s), 119.1 (s), 116.1 (m, C_6_F_5_), 113.7 (s), 84.3 (s), 73.6 (s), 66.9 (s), 66.1 (s), 55.6 (s), 19.2 (s), 13.9 (s), 12.2 (s). ^19^F-NMR (376 MHz, CDCl_3_, 298 K) *crystals*: −129.89 (br. s, 2F, *o*-F), 147.07 (br. s, 1F, *p*-F), −160.89 (m, 2F, *m*-F). Elemental analysis calcd (%) for C_32_H_16_BF_15_O_3_: C 51.64, H 2.17; Obs. C 51.54, H 2.07.

### 3.4. In Situ NMR Studies of Varying Concentration

#### 3.4.1. NMR Experiments of **2a**

Samples of 0.4, 0.2, 0.1 and 0.04 M concentration were prepared by dissolving the required amount of **2a** and B(C_6_F_5_)_3_ in a 1:1 ratio in CDCl_3_ (0.5 mL). ^1^H-NMR (500 MHz, CDCl_3_, 298 K, **0.4 M**): 7.87 (dd, 2H, ^3^*J*_HH_ = 8.7 Hz, ^4^*J*_HH_ = 1.2 Hz, *o*-ArH), 7.59 (br. tt, 1H, ^3^*J*_HH_ = 7.5, ^4^*J*_HH_ = 1.2 Hz, *p*-ArH), 7.40 (br. t, 2H, ^3^*J*_HH_ = 7.9, *m*-ArH), 4.58 (t, 2H, ^3^*J*_HH_ = 7.0 Hz, -CH_2_O(CO)-), 2.68 (tt, 2H, ^3^*J*_HH_ = 7.0 Hz, ^4^*J*_HH_ = 2.5 Hz, ‑C≡CCH_2_‑CH_2_-), 2.18 (qt, 2H, ^3^*J*_HH_ = 7.6 Hz, ^4^*J*_HH_ = 2.3 Hz, Me-CH_2_-C≡), 1.12 (t, 3H, ^3^*J*_HH_ = 7.6 Hz, -CH_3_). ^11^B-NMR (160 MHz, CDCl_3_, 298 K, **0.4 M**): 40.10, 28.51. ^19^F-NMR (376 MHz, CDCl_3_, 298 K, **0.4 M**): −132.07 (s, 2F, *o*-F), −151.36 (s, 1F, *p*-F), −162.18 (m, 2F, *m*-F). ^1^H-NMR (500 MHz, CDCl_3_, 298 K, **0.2 M**): 7.91 (dd, 2H, ^3^*J*_HH_ = 8.6 Hz, ^4^*J*_HH_ = 1.1 Hz, *o*-ArH), 7.59 (br. tt, 1H, ^3^*J*_HH_ = 7.5, ^4^*J*_HH_ = 1.2 Hz, *p*-ArH), 7.40 (br. t, 2H, ^3^*J*_HH_ = 8.1, *m*-ArH), 4.53 (t, 2H, ^3^*J*_HH_ = 6.7 Hz, -CH_2_O(CO)-), 2.67 (tt, 2H, ^3^*J*_HH_ = 7.0 Hz, ^4^*J*_HH_ = 2.5 Hz, ‑C≡CCH_2_‑CH_2_-), 2.17 (qt, 2H, ^3^*J*_HH_ = 7.6 Hz, ^4^*J*_HH_ = 2.3 Hz, Me-CH_2_-C≡), 1.12 (t, 3H, ^3^*J*_HH_ = 7.5 Hz, -CH_3_). ^11^B-NMR (160 MHz, CDCl_3_, 298 K, **0.2 M**): 40.20, 30.27. ^19^F-NMR (376 MHz, CDCl_3_, 298 K, **0.2 M**): −131.73 (s, 2F, *o*-F), −150.78 (s, 1F, *p*-F), −161.93 (m, 2F, *m*-F). ^1^H-NMR (500 MHz, CDCl_3_, 298 K, **0.1 M**): 8.0 (dd, 2H, ^3^*J*_HH_ = 8.2 Hz, ^4^*J*_HH_ = 1.4 Hz, *o*-ArH), 7.58 (br. tt, 1H, ^3^*J*_HH_ = 7.5, ^4^*J*_HH_ = 1.3 Hz, *p*-ArH), 7.41 (br. t, 2H, ^3^*J*_HH_ = 8.0, *m*-ArH), 4.49 (t, 2H, ^3^*J*_HH_ = 6.9 Hz, -CH_2_O(CO)-), 2.66 (tt, 2H, ^3^*J*_HH_ = 7.0 Hz, ^4^*J*_HH_ = 2.4 Hz, ‑C≡CCH_2_‑CH_2_-), 2.17 (qt, 2H, ^3^*J*_HH_ = 7.6 Hz, ^4^*J*_HH_ = 2.4 Hz, Me-CH_2_-C≡), 1.12 (t, 3H, ^3^*J*_HH_ = 7.5 Hz, -CH_3_). ^11^B-NMR (160 MHz, CDCl_3_, 298 K, **0.1 M**): 39.51. ^19^F-NMR (376 MHz, CDCl_3_, 298 K, **0.1 M**): −130.79 (s, 2F, *o*-F), −148.84 (s, 1F, *p*-F), −161.40 (m, 2F, *m*-F). ^1^H-NMR (500 MHz, CDCl_3_, 298 K, **0.04 M**): 8.0 (dd, 2H, ^3^*J*_HH_ = 8.5 Hz, ^4^*J*_HH_ = 1.1 Hz, *o*-ArH), 7.57 (br. tt, 1H, ^3^*J*_HH_ = 7.5, ^4^*J*_HH_ = 1.3 Hz, *p*-ArH), 7.43 (br. t, 2H, ^3^*J*_HH_ = 7.9, *m*-ArH), 4.45 (t, 2H, ^3^*J*_HH_ = 7.0 Hz, -CH_2_O(CO)-), 2.64 (tt, 2H, ^3^*J*_HH_ = 7.0 Hz, ^4^*J*_HH_ = 2.4 Hz, ‑C≡CCH_2_‑CH_2_-), 2.17 (qt, 2H, ^3^*J*_HH_ = 7.6 Hz, ^4^*J*_HH_ = 2.3 Hz, Me-CH_2_-C≡), 1.12 (t, 3H, ^3^*J*_HH_ = 7.5 Hz, -CH_3_). ^11^B-NMR (160 MHz, CDCl_3_, 298 K, **0.04 M**): 46.88. ^19^F-NMR (376 MHz, CDCl_3_, 298 K, **0.04 M**): −129.60 (s, 2F, *o*-F), −146.36 (s, 1F, *p*-F), −160.73 (m, 2F, *m*-F).

#### 3.4.2. NMR Experiments of **2b**

Samples of 0.4, 0.2, 0.1 and 0.04 M concentration were prepared by dissolving the required amount of a **2b** and B(C_6_F_5_)_3_ in a 1:1 ratio in CDCl_3_ (0.5 mL). ^1^H-NMR (500 MHz, CDCl_3_, 298 K, **0.4 M**): 7.75 (d, 2H, ^3^*J*_HH_ = 8.3 Hz, *o*-ArH), 7.18 (d, 2H, ^3^*J*_HH_ = 8.0 Hz, *m*-ArH), 4.58 (t, 2H, ^3^*J*_HH_ = 6.9 Hz, -CH_2_O(CO)-), 2.68 (tt, 2H, ^3^*J*_HH_ = 6.9 Hz, ^4^*J*_HH_ = 2.4 Hz, ‑C≡CCH_2_‑CH_2_-), 2.39 (s, 3H, Ar-CH_3_), 2.17 (qt, 2H, ^3^*J*_HH_ = 7.5 Hz, ^4^*J*_HH_ = 2.3 Hz, Me-CH_2_-C≡), 1.12 (t, 3H, ^3^*J*_HH_ = 7.5 Hz, -CH_3_). ^11^B-NMR (160 MHz, CDCl_3_, 298 K, **0.4 M**): 18.09, −16.63. ^19^F-NMR (376 MHz, CDCl_3_, 298 K, **0.4 M**): −133.11 (d, 2F, ^3^*J*_FF_ = 19.7 Hz, *o*-F), −153.76 (t, 1F, ^3^*J*_FF_ = 20.1 Hz, *p*-F), −162.85 (td, 2F, ^3^*J*_FF_ = 20.9 Hz, ^4^*J*_FF_ = 6.9 Hz, *m*-F). ^1^H-NMR (500 MHz, CDCl_3_, 298 K, **0.2 M**): 7.76 (d, 2H, ^3^*J*_HH_ = 8.2 Hz, *o*-ArH), 7.19 (d, 2H, ^3^*J*_HH_ = 8.2 Hz, *m*-ArH), 4.55 (t, 2H, ^3^*J*_HH_ = 6.9 Hz, -CH_2_O(CO)-), 2.67 (tt, 2H, ^3^*J*_HH_ = 6.9 Hz, ^4^*J*_HH_ = 2.3 Hz, ‑C≡CCH_2_‑CH_2_-), 2.39 (s, 3H, Ar-CH_3_), 2.17 (qt, 2H, ^3^*J*_HH_ = 7.6 Hz, ^4^*J*_HH_ = 2.4 Hz, Me-CH_2_-C≡), 1.12 (t, 3H, ^3^*J*_HH_ = 7.5 Hz, -CH_3_). ^11^B-NMR (160 MHz, CDCl_3_, 298 K, **0.2 M**): 23.07. ^19^F-NMR (376 MHz, CDCl_3_, 298 K, **0.2 M**): −132.58 (d, 2F, ^3^*J*_FF_ = 18.4 Hz, *o*-F), −152.60 (t, 1F, ^3^*J*_FF_ = 19.8 Hz, *p*-F), −162.46 (td, 2F, ^3^*J*_FF_ = 20.7 Hz, ^4^*J*_FF_ = 6.9 Hz, *m*-F). ^1^H-NMR (500 MHz, CDCl_3_, 298 K, **0.1 M**): 7.80 (br. d, 2H, ^3^*J*_HH_ = 8.4 Hz, *o*-ArH), 7.20 (d, 2H, ^3^*J*_HH_ = 8.0 Hz, *m*-ArH), 4.50 (t, 2H, ^3^*J*_HH_ = 7.0 Hz, -CH_2_O(CO)-), 2.65 (tt, 2H, ^3^*J*_HH_ = 7.0 Hz, ^4^*J*_HH_ = 2.4 Hz, ‑C≡CCH_2_‑CH_2_-), 2.40 (s, 3H, Ar-CH_3_), 2.17 (qt, 2H, ^3^*J*_HH_ = 7.6 Hz, ^4^*J*_HH_ = 2.4 Hz, Me-CH_2_-C≡), 1.11 (t, 3H, ^3^*J*_HH_ = 7.5 Hz, -CH_3_). ^11^B-NMR (160 MHz, CDCl_3_, 298 K, **0.1 M**): 27.34. ^19^F-NMR (376 MHz, CDCl_3_, 298 K, **0.1 M**): −132.13 (d, 2F, ^3^*J*_FF_ = 19.7 Hz, *o*-F), −152.60 (br. s, 1F, *p*-F), −162.16 (td, 2F, ^3^*J*_FF_ = 21.8 Hz, ^4^*J*_FF_ = 6.8 Hz, *m*-F). ^1^H-NMR (500 MHz, CDCl_3_, 298 K, **0.04 M**): 7.84 (br. d, 2H, ^3^*J*_HH_ = 8.4 Hz, *o*-ArH), 7.21 (d, 2H, ^3^*J*_HH_ = 8.1 Hz, *m*-ArH), 4.46 (t, 2H, ^3^*J*_HH_ = 6.9 Hz, -CH_2_O(CO)-), 2.64 (tt, 2H, ^3^*J*_HH_ = 6.9 Hz, ^4^*J*_HH_ = 2.3 Hz, ‑C≡CCH_2_‑CH_2_-), 2.40 (s, 3H, Ar-CH_3_), 2.17 (qt, 2H, ^3^*J*_HH_ = 7.6 Hz, ^4^*J*_HH_ = 2.4 Hz, Me-CH_2_-C≡), 1.11 (t, 3H, ^3^*J*_HH_ = 7.5 Hz, -CH_3_). ^11^B-NMR (160 MHz, CDCl_3_, 298 K, **0.04 M**): 43.58. ^19^F-NMR (376 MHz, CDCl_3_, 298 K, **0.04 M**): −130.20 (d, 2F, ^3^*J*_FF_ = 16.4 Hz, *o*-F), −147.64 (br. s, 1F, *p*-F), −161.08 (br. t, 2F, ^3^*J*_FF_ = 17.4 Hz, *m*-F).

#### 3.4.3. NMR Experiments of **2c**

Samples of 0.4, 0.2, 0.1 and 0.04 M concentration were prepared by dissolving the required amount of **2c** and B(C_6_F_5_)_3_ in a 1:1 ratio in CDCl_3_ (0.5 mL). ^1^H-NMR (500 MHz, CDCl_3_, 298 K, **0.4 M**): 7.86 (d, 2H, ^3^*J*_HH_ = 8.9 Hz, *o*-ArH), 6.84 (d, 2H, ^3^*J*_HH_ = 9.0 Hz, *m*-ArH), 4.55 (t, 2H, ^3^*J*_HH_ = 6.9 Hz, -CH_2_O(CO)-), 3.85 (s, 3H, -OCH_3_), 2.67 (br. tt, 2H, ^3^*J*_HH_ = 6.9 Hz, ^4^*J*_HH_ = 2.2 Hz, ‑C≡CCH_2_‑CH_2_-), 2.17 (qt, 2H, ^3^*J*_HH_ = 7.5 Hz, ^4^*J*_HH_ = 2.3 Hz, Me-CH_2_-C≡), 1.11 (t, 3H, ^3^*J*_HH_ = 7.5 Hz, -CH_3_). ^11^B-NMR (160 MHz, CDCl_3_, 298 K, **0.4 M**): 40.16, 10.33. ^19^F-NMR (376 MHz, CDCl_3_, 298 K, **0.4 M**): −133.70 (d, 2F, ^3^*J*_FF_ = 19.2 Hz, *o*-F), 154.94 (br. s, 1F, *p*-F), −163.16 (t, 2F, ^3^*J_FF_ =* 17.5 Hz, *m*-F). ^1^H-NMR (500 MHz, CDCl_3_, 298 K, **0.2 M**): 7.87 (d, 2H, ^3^*J*_HH_ = 9.03 Hz, *o*-ArH), 6.84 (d, 2H, ^3^*J*_HH_ = 8.9 Hz, *m*-ArH), 4.51 (t, 2H, ^3^*J*_HH_ = 6.9 Hz, -CH_2_O(CO)-), 3.85 (s, 3H, -OCH_3_), 2.66 (br. tt, 2H, ^3^*J*_HH_ = 6.9 Hz, ^4^*J*_HH_ = 2.3 Hz, ‑C≡CCH_2_‑CH_2_-), 2.17 (qt, 2H, ^3^*J*_HH_ = 7.4 Hz, ^4^*J*_HH_ = 2.3 Hz, Me-CH_2_-C≡), 1.11 (t, 3H, ^3^*J*_HH_ = 7.7 Hz, -CH_3_). ^11^B-NMR (160 MHz, CDCl_3_, 298 K, **0.2 M**): 39.88, 12.71. ^19^F-NMR (376 MHz, CDCl_3_, 298 K, **0.2 M**): −133.45 (d, 2F, ^3^*J*_FF_ = 19.2 Hz, *o*-F), 154.36 (t, 1F, ^3^*J*_FF_ = 19.4 Hz, *p*-F), −162.91 (td, 2F, ^3^*J_FF_ =* 21.9, ^4^*J*_FF_ = 6.5 Hz, *m*-F). ^1^H-NMR (500 MHz, CDCl_3_, 298 K, **0.1 M**): 7.88 (br. d, 2H, ^3^*J*_HH_ = 8.9 Hz, *o*-ArH), 6.85 (br. d, 2H, ^3^*J*_HH_ = 8.9 Hz, *m*-ArH), 4.49 (t, 2H, ^3^*J*_HH_ = 6.9 Hz, -CH_2_O(CO)-), 3.86 (s, 3H, -OCH_3_), 2.65 (br. tt, 2H, ^3^*J*_HH_ = 7.0 Hz, ^4^*J*_HH_ = 2.4 Hz, ‑C≡CCH_2_‑CH_2_-), 2.16 (qt, 2H, ^3^*J*_HH_ = 7.5 Hz, ^4^*J*_HH_ = 2.4 Hz, Me-CH_2_-C≡), 1.11 (t, 3H, ^3^*J*_HH_ = 7.5 Hz, -CH_3_). ^11^B-NMR (160 MHz, CDCl_3_, 298 K, **0.1 M**): 40.27, 19.46. ^19^F-NMR (376 MHz, CDCl_3_, 298 K, **0.1 M**): −132.83 (d, 2F, ^3^*J*_FF_ = 19.2 Hz, *o*-F), 153.08 (br. s, 1F, *p*-F), −162.52 (td, 2F, ^3^*J_FF_ =* 21.9 Hz, ^4^*J*_FF_ = 7.1 Hz, *m*-F). ^1^H-NMR (500 MHz, CDCl_3_, 298 K, **0.04 M**): 7.91 (br. d, 2H, ^3^*J*_HH_ = 8.9 Hz, *o*-ArH), 6.87 (br. d, 2H, ^3^*J*_HH_ = 9.0 Hz, *m*-ArH), 4.46 (t, 2H, ^3^*J*_HH_ = 6.9 Hz, -CH_2_O(CO)-), 3.86 (s, 3H, -OCH_3_), 2.64 (tt, 2H, ^3^*J*_HH_ = 7.1 Hz, ^4^*J*_HH_ = 2.4 Hz, ‑C≡CCH_2_‑CH_2_-), 2.17 (qt, 2H, ^3^*J*_HH_ = 7.5 Hz, ^4^*J*_HH_ = 2.4 Hz, Me-CH_2_-C≡), 1.11 (t, 3H, ^3^*J*_HH_ = 7.5 Hz, -CH_3_). ^11^B-NMR (160 MHz, CDCl_3_, 298 K, **0.04 M**): 39.45, 27.72. ^19^F-NMR (376 MHz, CDCl_3_, 298 K, **0.04 M**): −132.00 (d, 2F, ^3^*J*_FF_ = 19.2 Hz, *o*-F), 151.37 (s, 1F, *p*-F), −162.04 (td, 2F, ^3^*J_FF_ =* 20.1 Hz, ^4^*J*_FF_ = 8.1 Hz, *m*-F).

### 3.5. In Situ NMR Studies of Varying Stoichiometry

Two separate samples were made up with a 10:1 excess of **2b** (10 mg, 0.05 mmol) over B(C_6_F_5_)_3_ (256 mg, 0.5 mmol) in one, then conversely a 10:1 excess of B(C_6_F_5_)_3_ (50 mg, 0.1 mmol) over **2b** (216 mg, 1 mmol) all in in all the other, all in CDCl_3_ (0.5 mL).

#### 3.5.1. Excess **2b**

^1^H-NMR (500 MHz, CDCl_3_, 298 K): 7.70 (d, 2H, ^3^*J*_HH_ = 8.4 Hz, *o*-ArH), 7.18 (d, 2H, ^3^*J*_HH_ = 8.0 Hz, *m*-ArH), 4.65 (t, 2H, ^3^*J*_HH_ = 6.8 Hz, -CH_2_O(CO)-), 2.71 (tt, 2H, ^3^*J*_HH_ = 6.8 Hz, ^4^*J*_HH_ = 2.4 Hz, ‑C≡CCH_2_‑CH_2_-), 2.40 (s, 3H, Ar-CH_3_), 2.18 (qt, 2H, ^3^*J*_HH_ = 7.5 Hz, ^4^*J*_HH_ = 2.4 Hz, Me-CH_2_-C≡), 1.13 (t, 3H, ^3^*J*_HH_ = 7.5 Hz, -CH_3_). ^11^B-NMR (160 MHz, CDCl_3_, 298 K): 53.05. ^19^F-NMR (376 MHz, CDCl_3_, 298 K): −128.62 (d, 2F, ^3^*J*_FF_ = 20.7 Hz, *o*-F), −144.31 (br. s, 1F, *p*-F), −160.60 (td, 2F, ^3^*J*_FF_ = 20.5 Hz, ^4^*J*_FF_ = 7.5 Hz, *m*-F).

#### 3.5.2. Excess B(C_6_F_5_)_3_

^1^H-NMR (500 MHz, CDCl_3_, 298 K): 7.90 (d, 2H, ^3^*J*_HH_ = 8.3 Hz, *o*-ArH), 7.18 (d, 2H, ^3^*J*_HH_ = 8.1 Hz, *m*-ArH), 4.36 (t, 2H, ^3^*J*_HH_ = 7.0 Hz, -CH_2_O(CO)-), 2.59 (tt, 2H, ^3^*J*_HH_ = 7.1 Hz, ^4^*J*_HH_ = 2.4 Hz, ‑C≡CCH_2_‑CH_2_-), 2.35 (s, 3H, Ar-CH_3_), 2.13 (qt, 2H, ^3^*J*_HH_ = 7.5 Hz, ^4^*J*_HH_ = 2.4 Hz, Me-CH_2_-C≡), 1.08 (t, 3H, ^3^*J*_HH_ = 7.5 Hz, -CH_3_). ^11^B-NMR (160 MHz, CDCl_3_, 298 K): 3.32. ^19^F-NMR (376 MHz, CDCl_3_, 298 K): −134.27 (d, 2F, ^3^*J*_FF_ = 19.8 Hz, *o*-F), −156.03 (t, 1F, ^3^*J*_FF_ = 20.7 Hz, *p*-F), −163.26 (t, 2F, ^3^*J*_FF_ = 20.4 Hz, *m*-F).

### 3.6. Crystallographic Studies

Single crystals of **2a**–**c** were grown under an inert atmosphere and protected from atmospheric air and moisture using an inert per-fluorinated polyether oil. Single crystals of **2a**–**c** were mounted in a cryoloop and crystallographic data collected on an Agilent Dual SuperNova diffractometer using monochromatic Cu-Kα radiation (1.54184 Å) and a CCD area detector. Data were collected at 150(2) K (**2a**,**c**) or 200(2) K (**2b**). Data collection and processing implemented CrysalisPro [47] and a gaussian absorption correction applied within the CrysalisPro suite. The structures were solved by direct methods and refined against *F^2^* using the SHELXTL package [48]. In the case of **2a**, a region of diffuse electron density was treated with SQUEEZE incorporated within the PLATON package [49] with both the void volume and electron count corresponding to one toluene molecule per unit cell. The structures have been deposited with the Cambridge Crystallographic Data Centre under CCDC deposition numbers 1046813-1046815. Crystallographic data for **2a**–**2c** are presented in Table 4.

**Table 4:** Crystallographic data for compounds **2a**–**2c**.

Compound	2a	2b	2c
Formula	C_31_H_14_BF_15_O_2_ *	C_32_H_16_BF_15_O_2_	C_32_H_16_BF_15_O_3_
M	714.23	728.26	744.26
Crystal System	Triclinic	Triclinic	Triclinic
Space Group	*P-1*	*P-1*	*P-1*
a	10.9048(8)	10.5395(4)	11.1660(5)
b	11.4463(6)	11.3370(4)	12.5550(5)
c	13.7289(6)	13.2062(4)	12.6939(4)
α	84.827(4)	102.149(3)	78.409(3)
β	74.262(5)	97.888(3)	68.678(4)
γ	63.545(6)	94.962(3)	65.281(4)
V	1475.75(16)	1517.15(9)	1503.32(13)
T/K	150(2)	200(2)	150(2)
Z	2	2	2
D_c_	1.607	1.594	1.644
θmin, θmax	4.264–73.720	3.468–74.078	3.744–73.995
Crystal size	0.26 × 0.08 × 0.07	0.33 × 0.28 × 0.23	0.46 × 0.34 × 0.12
μ/mm^−1^	1.479	1.451	1.505
F(000)	712	728	744
Total Reflections	9959	10254	10549
Independent Reflections	5788	5926	5876
Rint	0.0211	0.0164	0.0164
*R*_1_ (I > 2s(I))	0.0349	0.0439	0.0323
*wR*_2_ (all data)	0.1043	0.1377	0.0945
*S*	1.020	1.045	1.013
Min/max e^−^/Å^3^	+0.28/−0.27	+0.78/−0.31	+0.30/−0.24

* Excludes 0.5 DCM solvent of crystallization estimated from SQUEEZE.

### 3.7. Computational Studies

DFT calculations were undertaken within Jaguar [50]. Initial geometry optimizations implemented the B3LYP functional [51] and Pople double zeta 6-31G* basis set [52]. Additional triple zeta calculations were performed using the 6-311G* basis set for the thermodynamic calculations and zero point energy corrections made.

## 4. Conclusions 

In conclusion, we have synthesized a range of alkynyl benzoates bearing both ester and alkyne functionalities and have investigated their reactivities with the strong Lewis acid B(C_6_F_5_)_3_. Since both σ-activation of the carbonyl and π-activation of the alkyne are possible, we have observed that in these cases σ-activation dominates and adduct formation occurs. In these cases no 1,2-addition product from the addition of the ester and the boron across the alkyne could be isolated since the carbocation that would be formed is not very stable. This is different to the reactions seen previously with propargyl amides which do undergo 1,2-addition of the amide and B(C_6_F_5_)_3_ across the alkyne. In this case the positive charge is more stable since nitrogen is better able to stabilize a positive charge than oxygen. In the case of propargyl esters 1,2-addition does occur and the cyclized 1,2-addition product can be isolated however, this species undergoes rapid rearrangement to afford a more thermodynamically stable product. The investigation of the reactivities of alkynyl benzoates bearing terminal or internal alkynes in 1,2-addition reactions with external nucleophiles are currently being undertaken and will be the focus of our future studies. In addition, the reactions of these compounds (and their derivatives) in 1,1-carboboration reactions will also be investigated.

## Supplementary Materials

Supplementary materials can be accessed at: http://www.mdpi.com/1420-3049/20/03/4530/s1.
