# Supplementary material for: σ- versus π-Activation of Alkynyl Benzoates Using B(C6F5)3
Source: Molecules. 2015 Mar 12;20(3):4530–47. doi: 10.3390/molecules20034530 (PMC6272168; doi:10.3390/molecules20034530)

Supplementary Information

1. Experimental Selected NMR Spectra

1.1. NMR Spectra of Starting Materials

^{1}H-NMR (500 MHz, $CDCl_3$, 298K) spectrum of hex-3-yn-1-yl benzoate (**1a**)

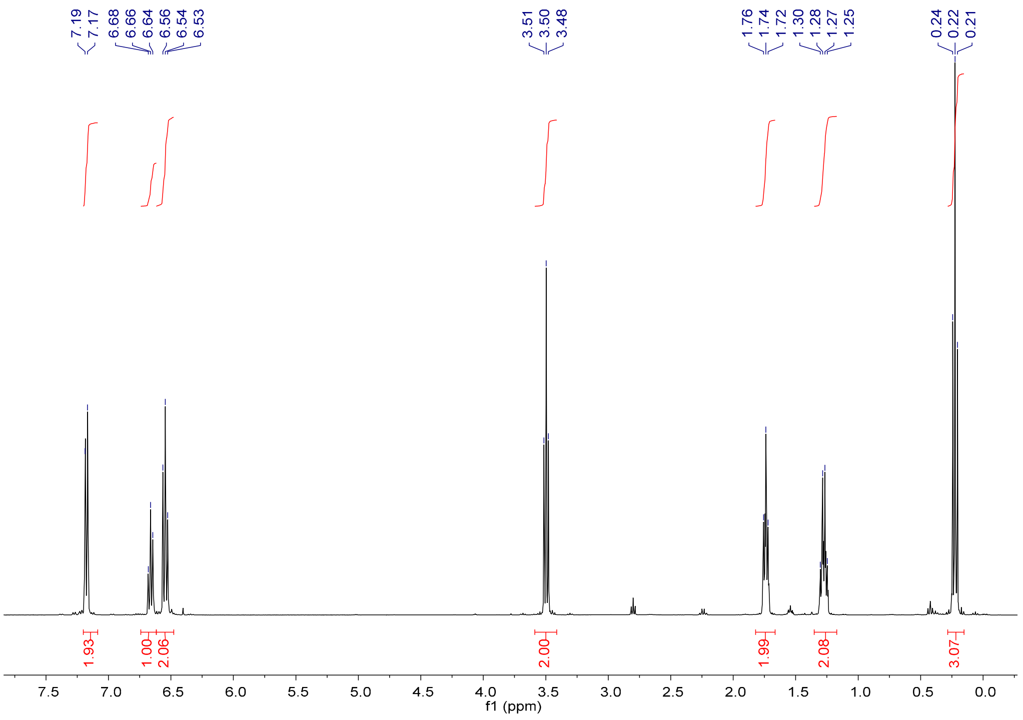

^{13}C-NMR (125 MHz, $CDCl_3$, 298K) spectrum of hex-3-yn-1-yl benzoate (**1a**)

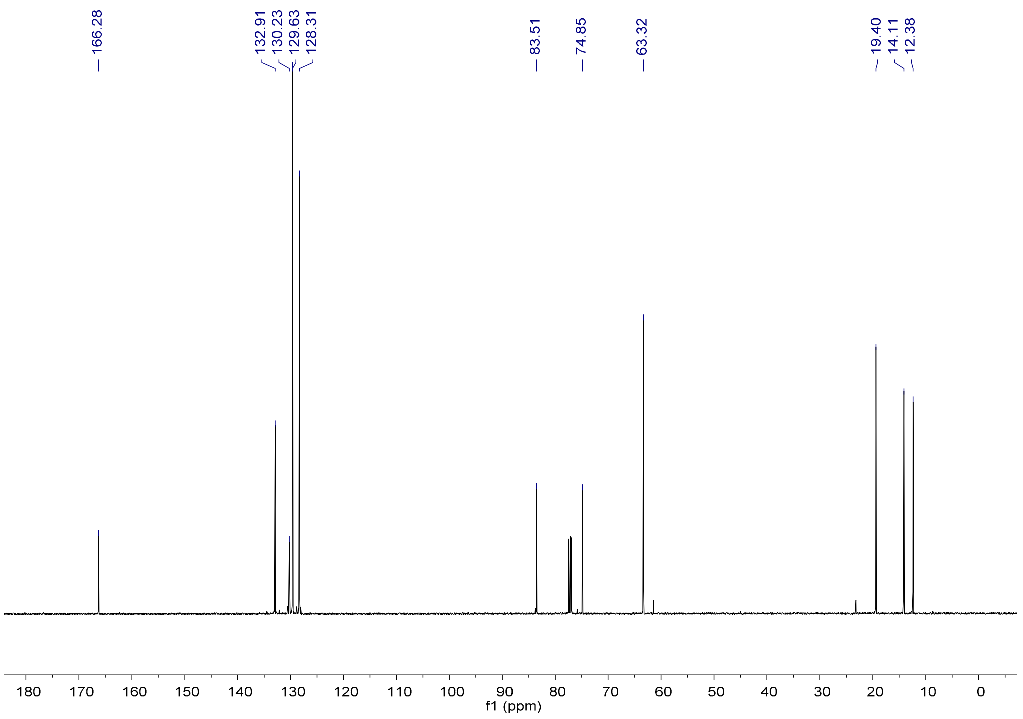

^{1}H-NMR (400 MHz, $CDCl_3$, 298K) spectrum of hex-3-yn-1-yl 4-methylbenzoate (**1b**)

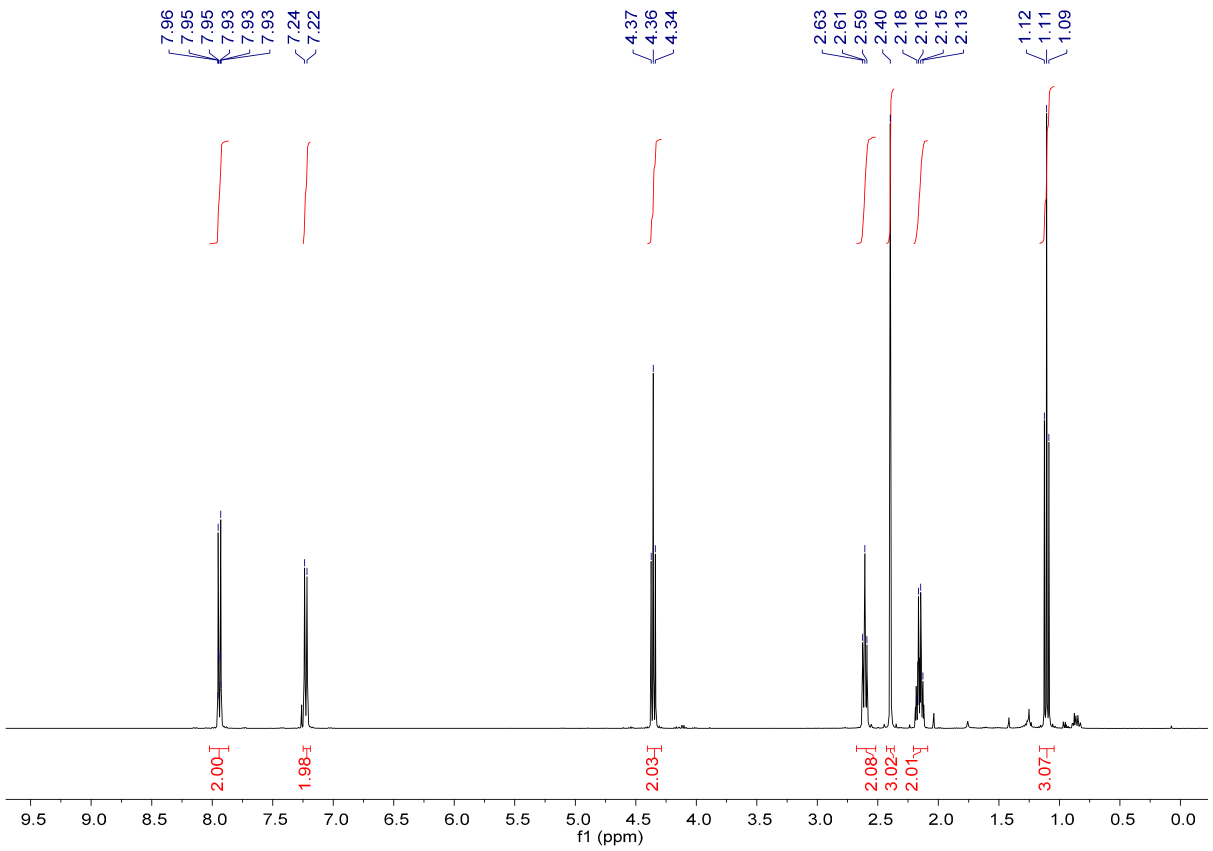

^{13}C-NMR (100.6 MHz, $CDCl_3$, 298K) spectrum of hex-3-yn-1-yl 4-methylbenzoate (**1b**)

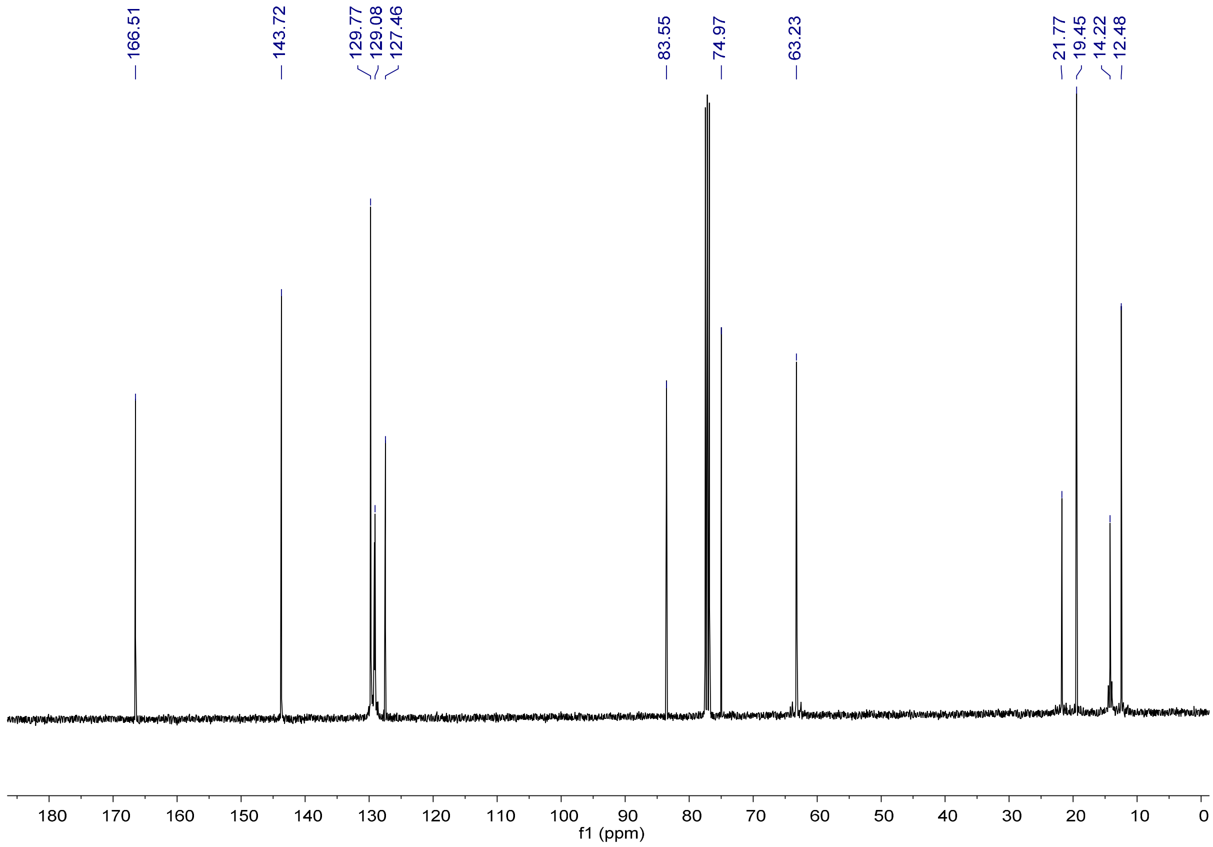

^{1}H-NMR (500 MHz, $CDCl_3$, 298K) spectrum of hex-3-yn-1-yl 4-methoxybenzoate (**1c**)

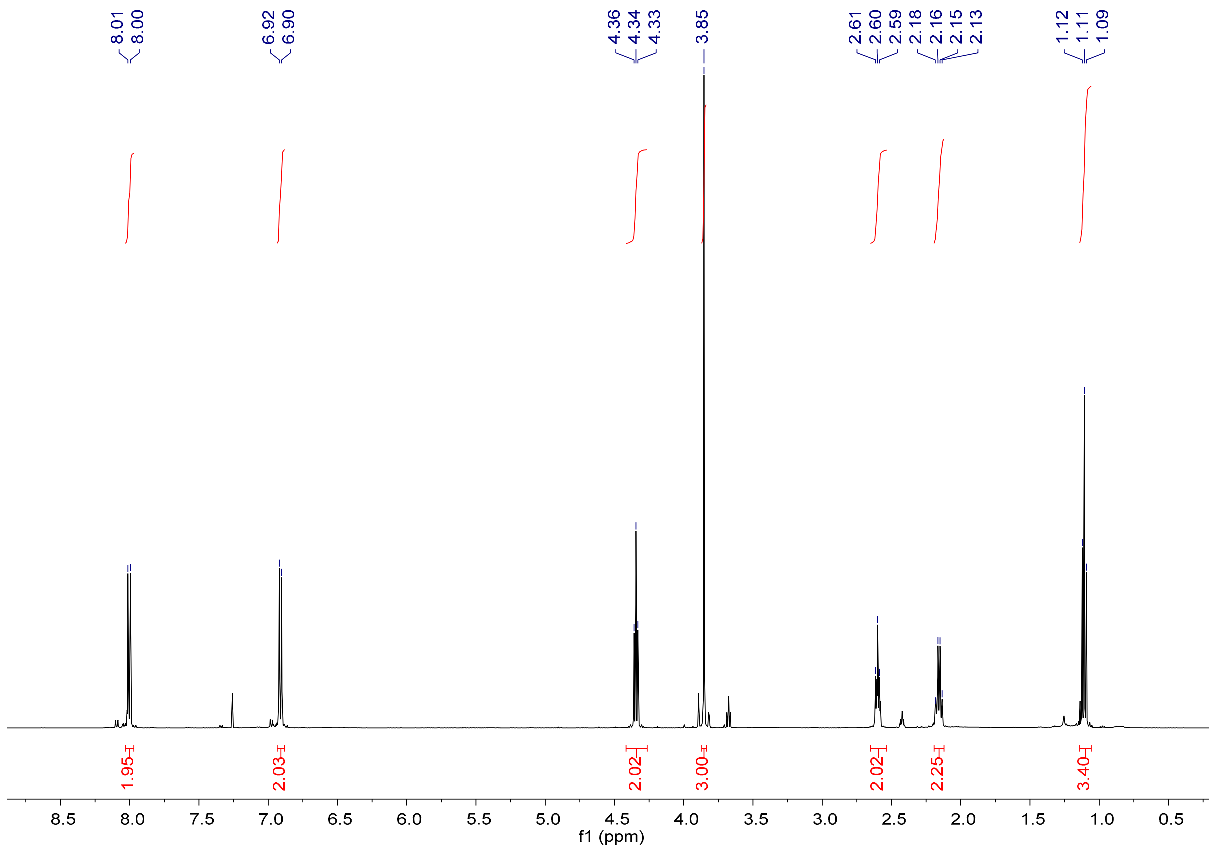

^{13}C-NMR (125 MHz, $CDCl_3$, 298K) spectrum of hex-3-yn-1-yl 4-methoxybenzoate (**1c**)

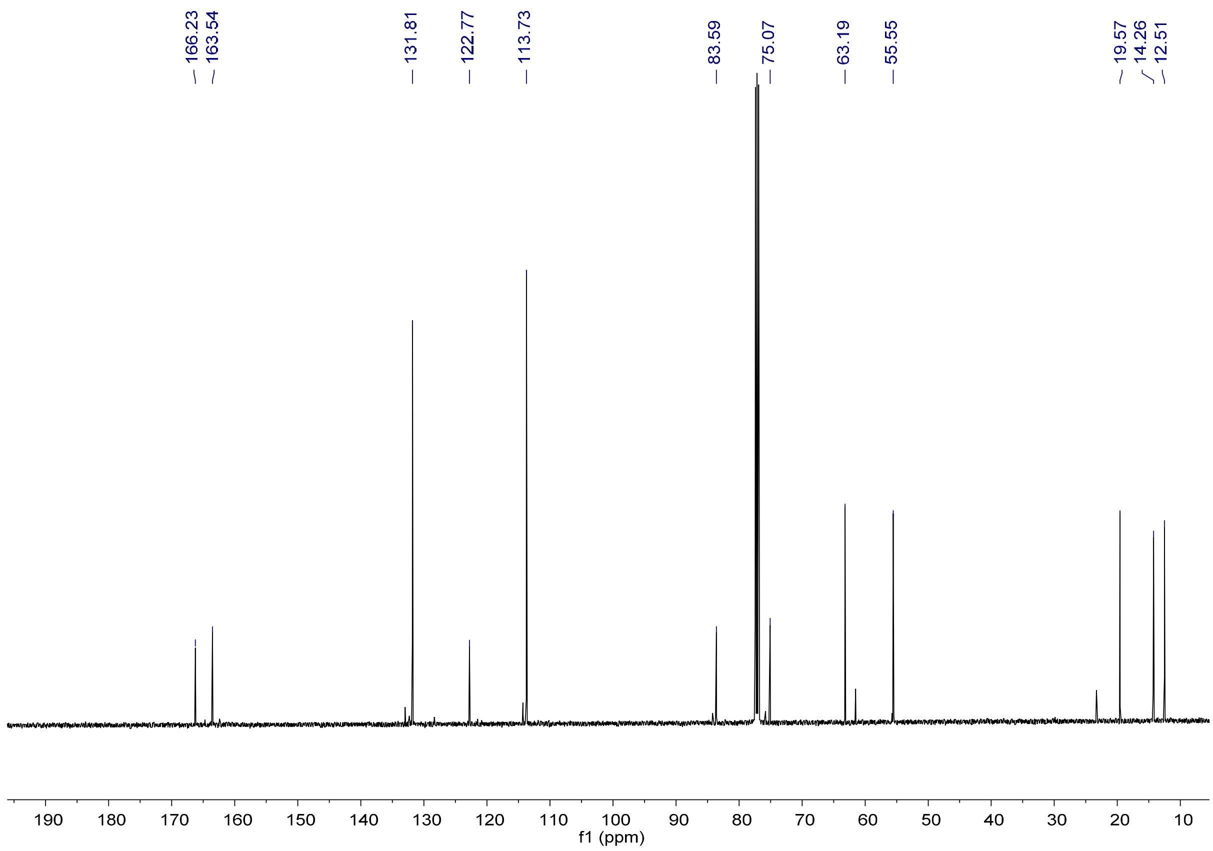

1.2. NMR Spectra of Products

^{1}H-NMR (500 MHz, $CDCl_3$, 298K) spectrum of **2a** crystals.

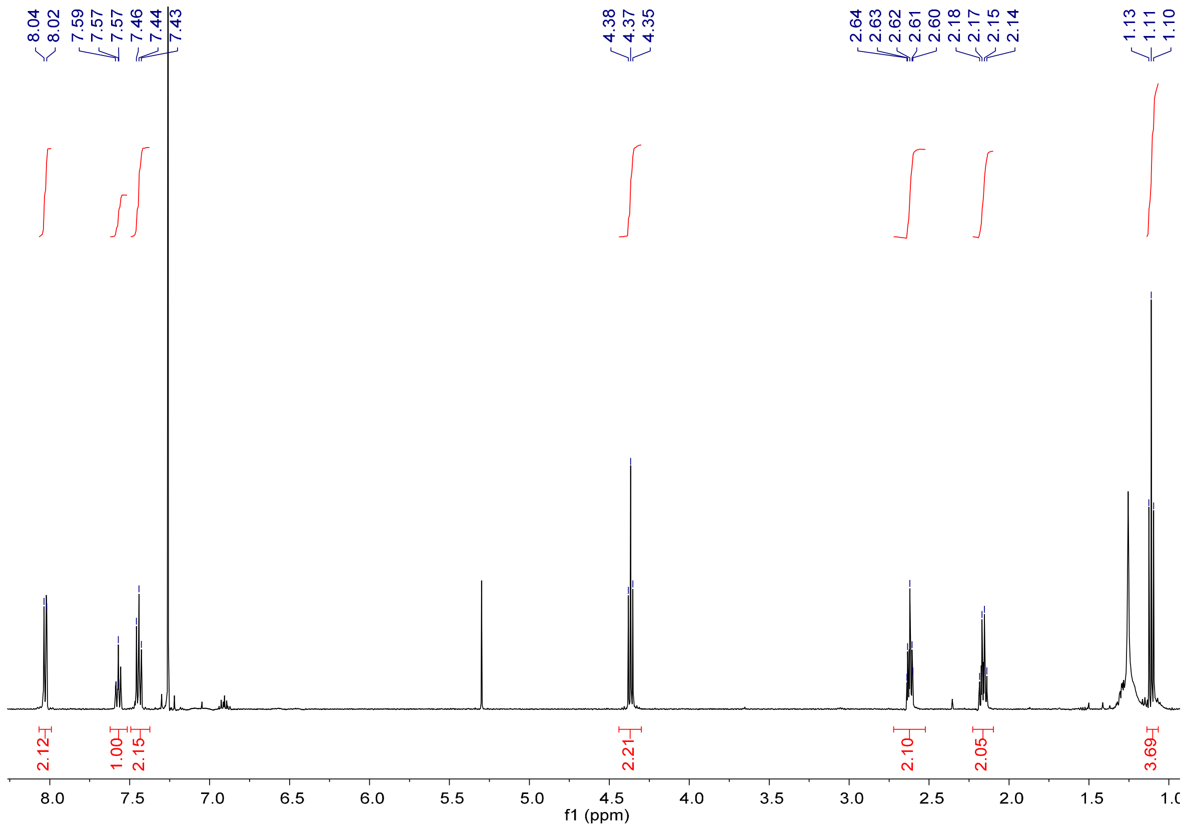

In situ ^{1}H-NMR (500 MHz, $CDCl_3$, 298K, 0.4 M) spectrum of **2a**.

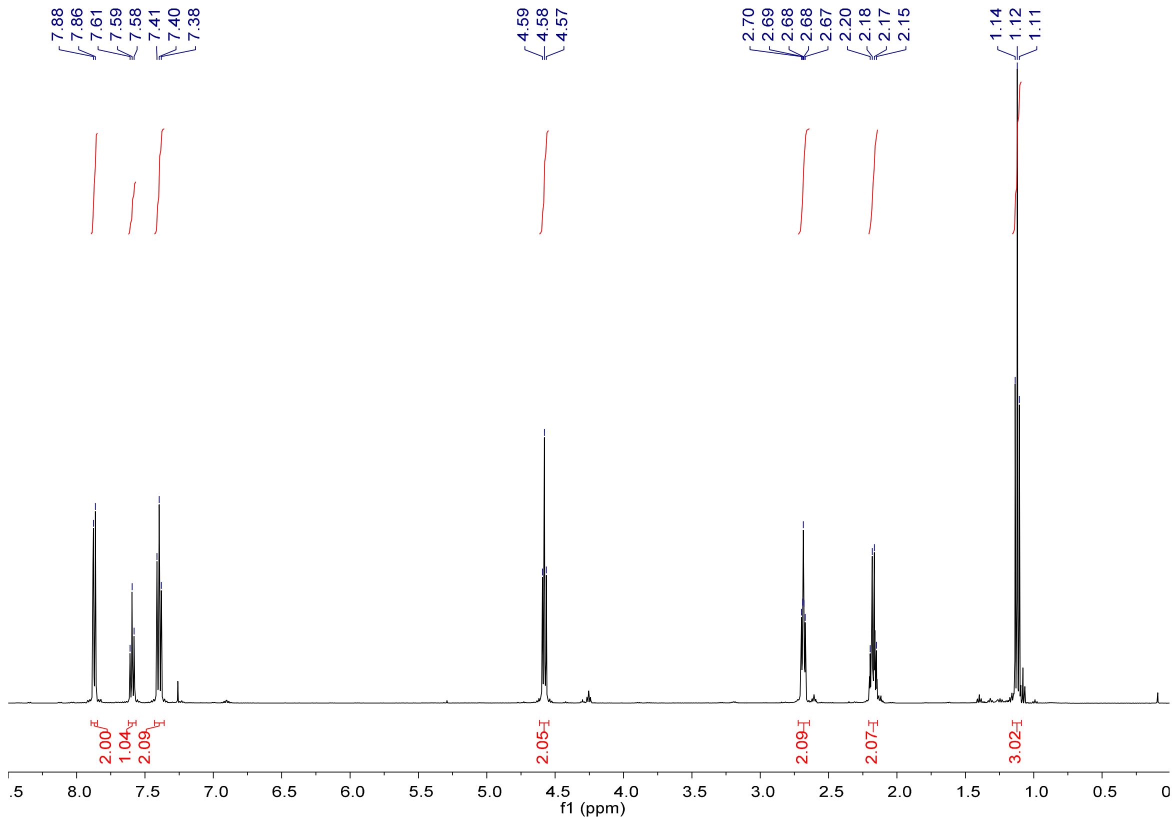

In situ ^{1}H-NMR (500 MHz, $CDCl_3$, 298K, 0.2 M) spectrum of **2a**.

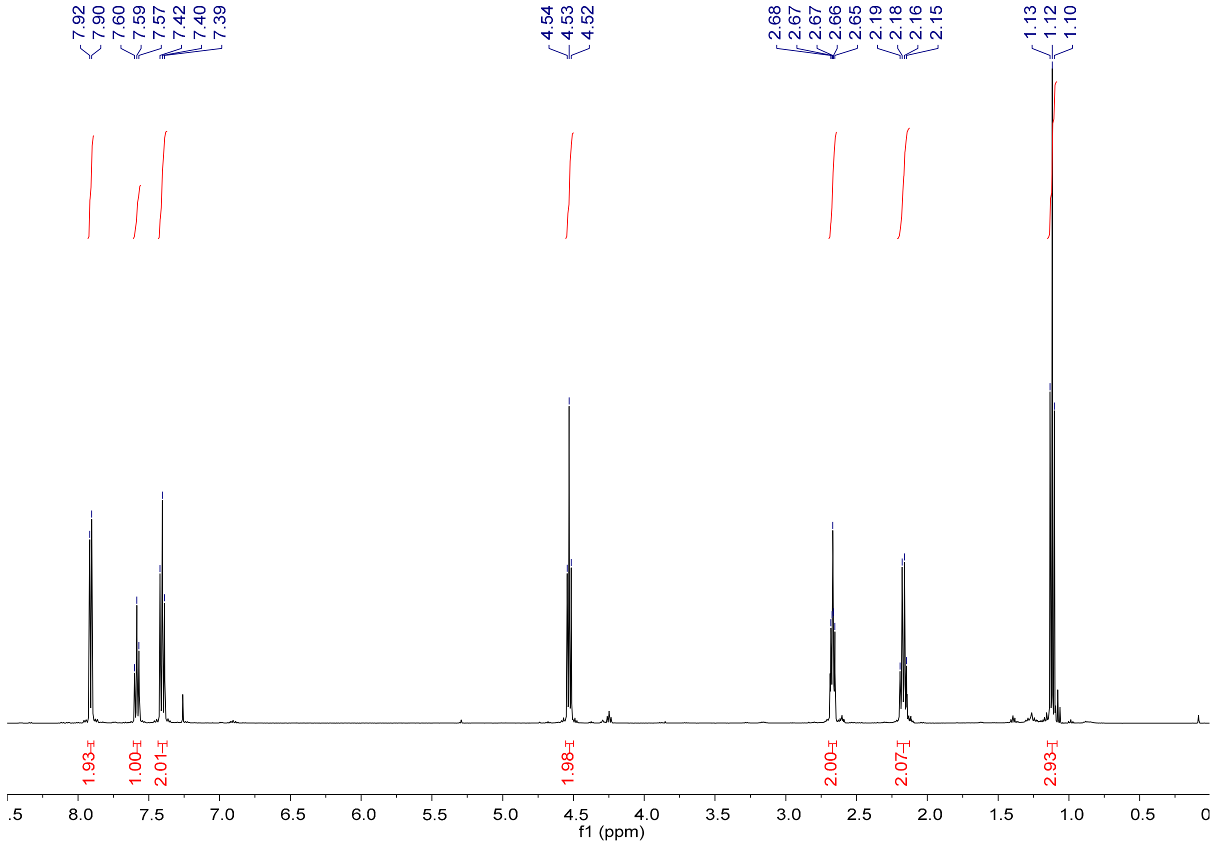

In situ ^{1}H-NMR (500 MHz, $CDCl_3$, 298K, 0.1 M) spectrum of **2a**.

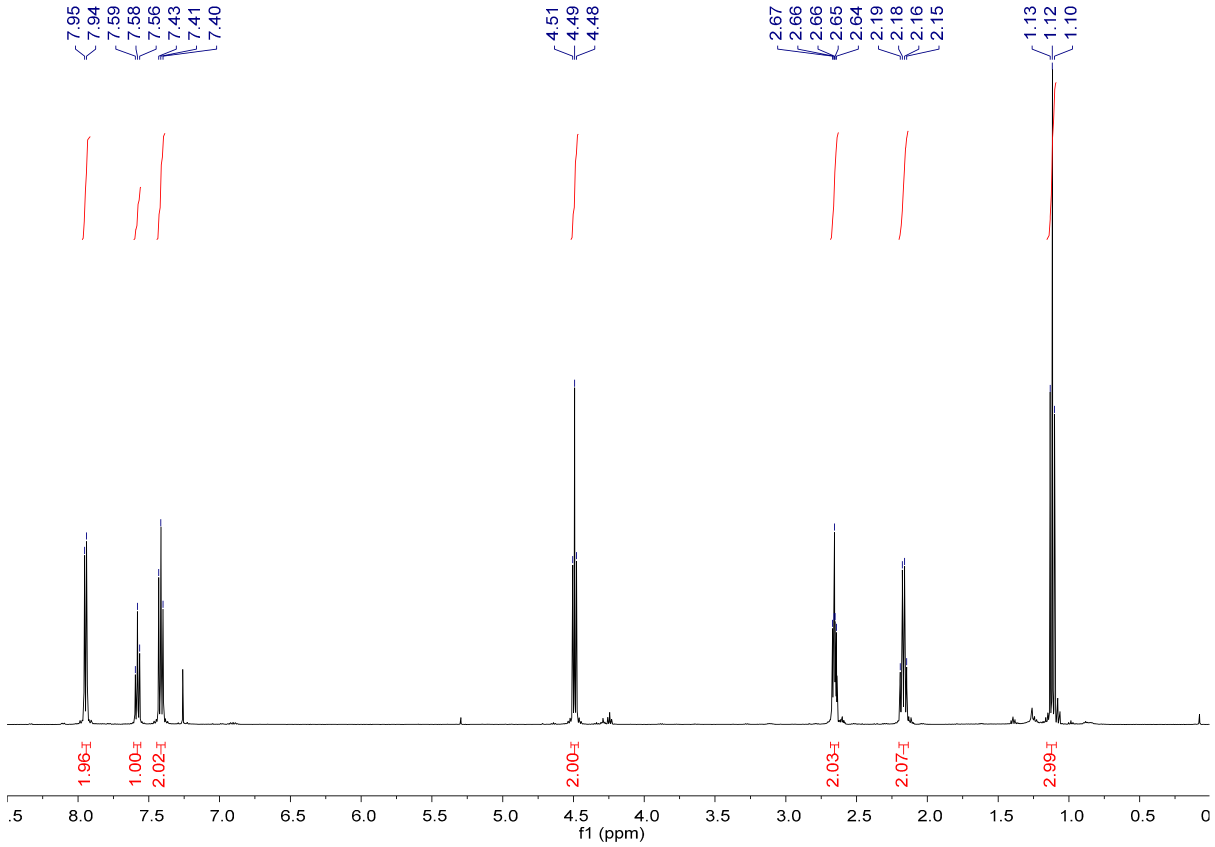

In situ ^{1}H-NMR (500 MHz, $CDCl_3$, 298K, 0.04 M) spectrum of **2a**.

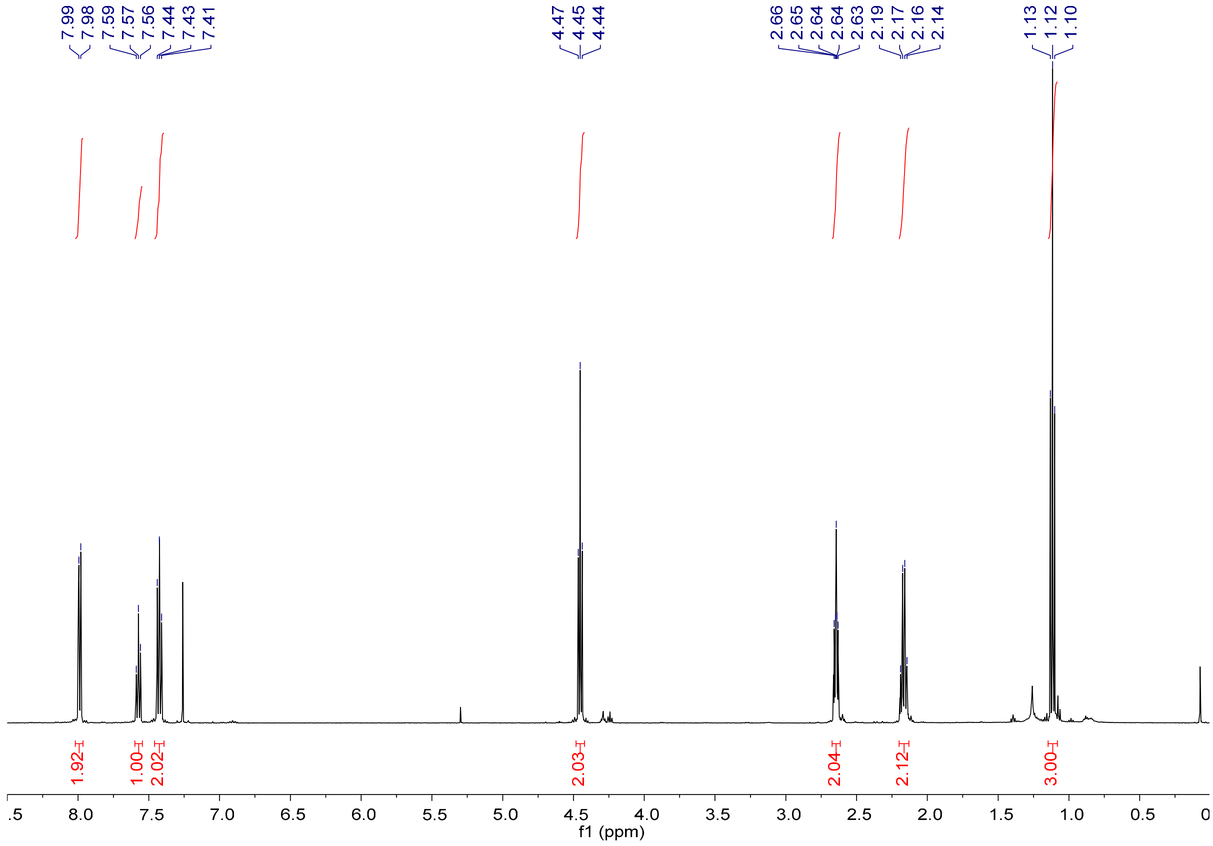

In situ ^{1}H-NMR (500 MHz, $CDCl_3$, 298K) spectra of **2a** across concentrations 0.04–0.4 M.

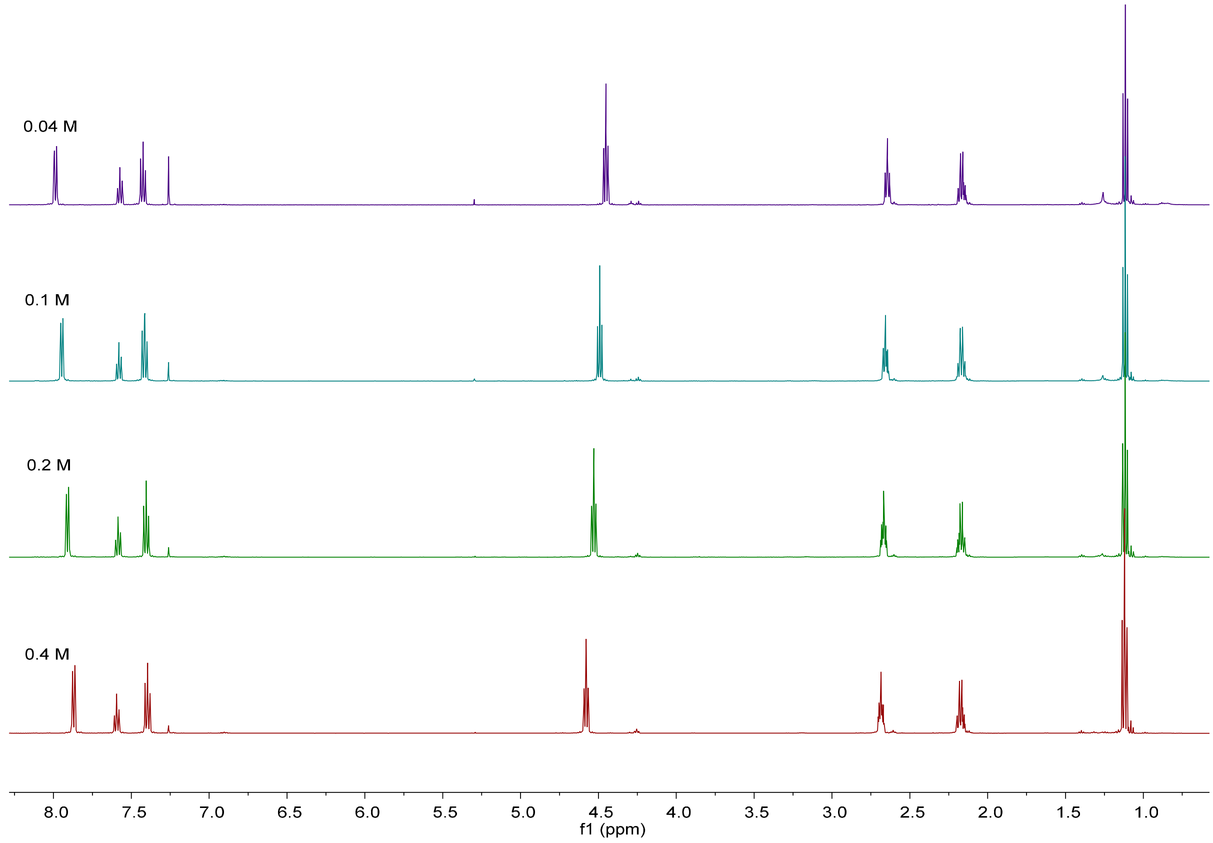

In situ ^{1}H-NMR (500 MHz, $CDCl_3$, 298K) specta of **2a,** expansion of C<u>H</u>$_2$O(CO)-) across concentrations 0.04–0.4 M.

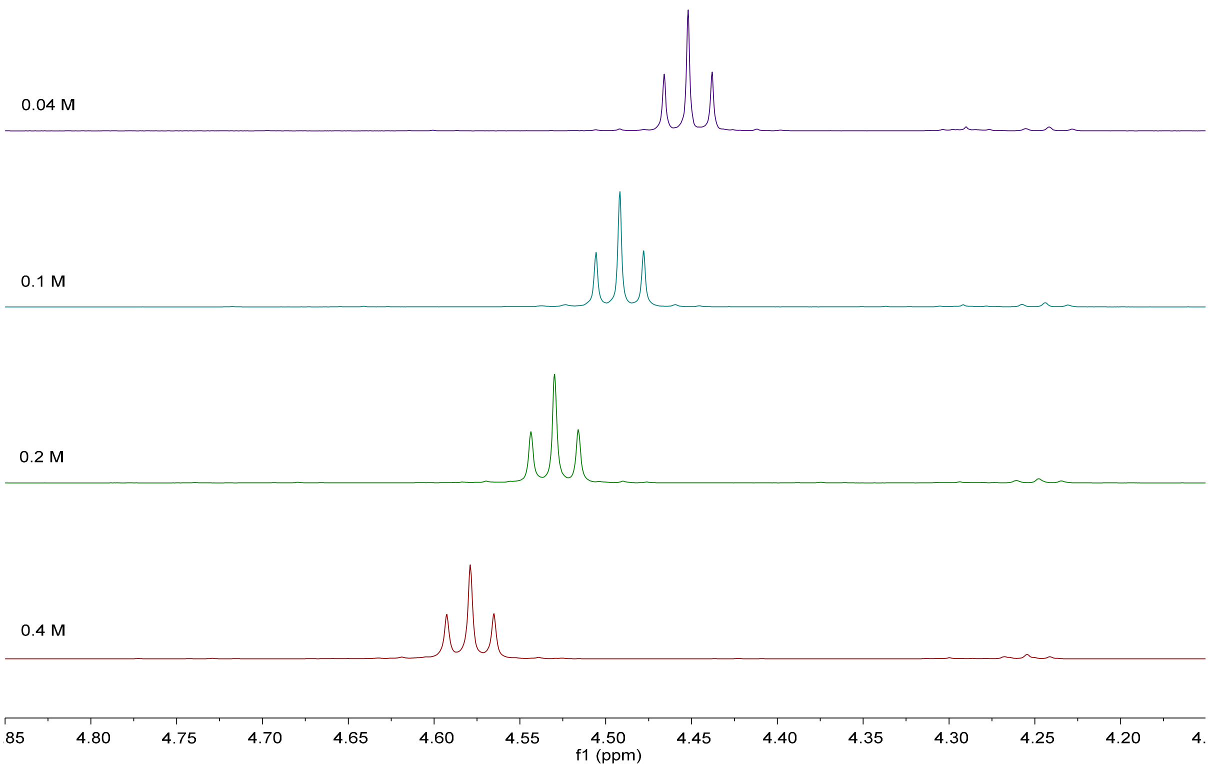

In situ ^{13}C-NMR (125 MHz, $CDCl_3$, 298K, 0.2 M) spectrum of **2a**.

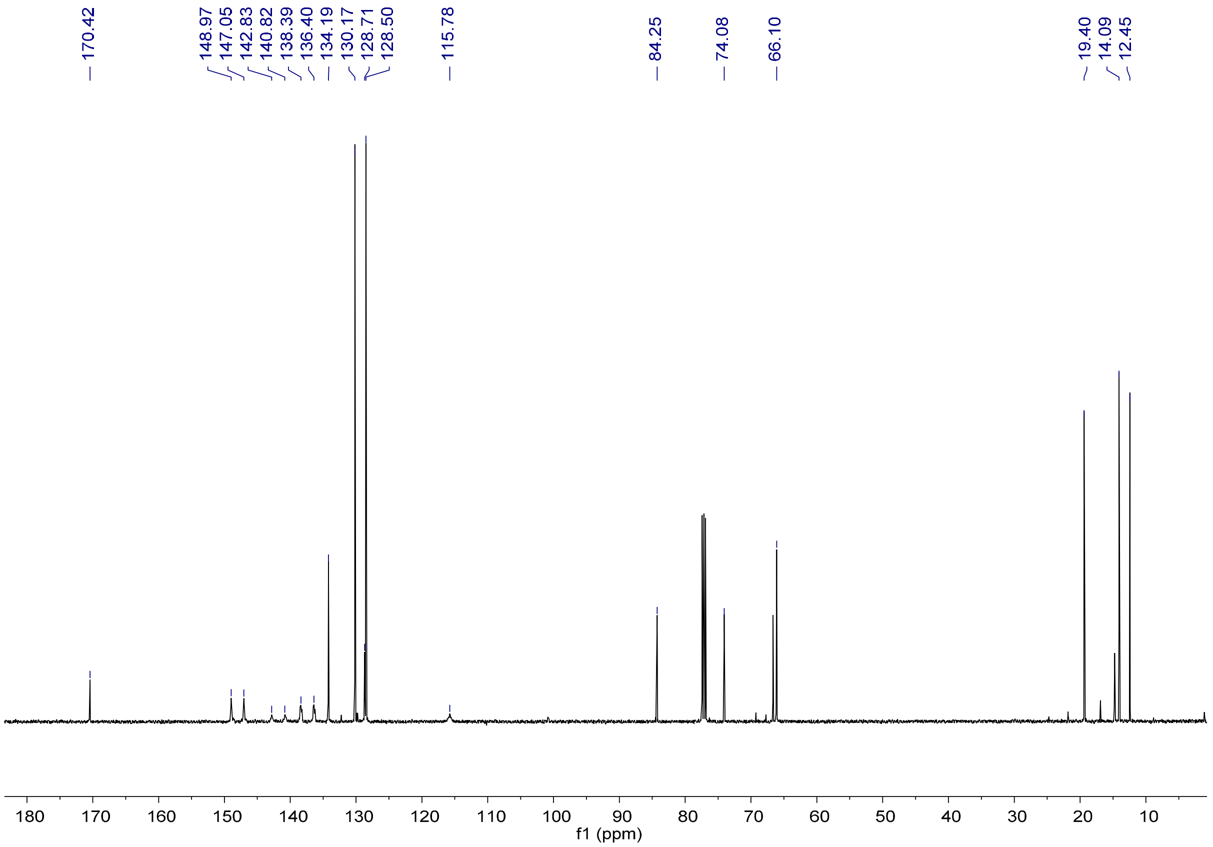

In situ ^{11}B-NMR (160 MHz, $CDCl_3$, 298 K, 0.4 M) spectrum of **2a**.

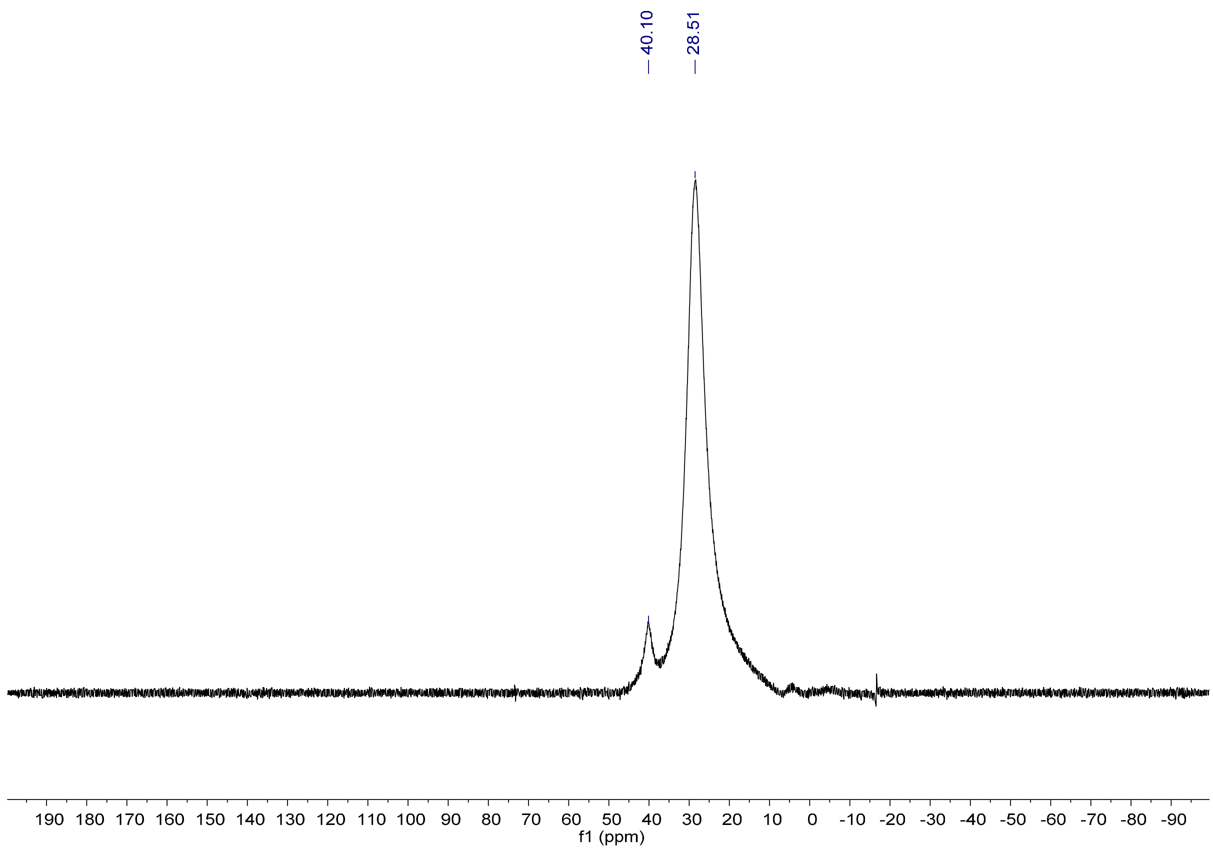

In situ ^{11}B-NMR (160 MHz, $CDCl_3$, 298 K, 0.2 M) spectrum of **2a**.

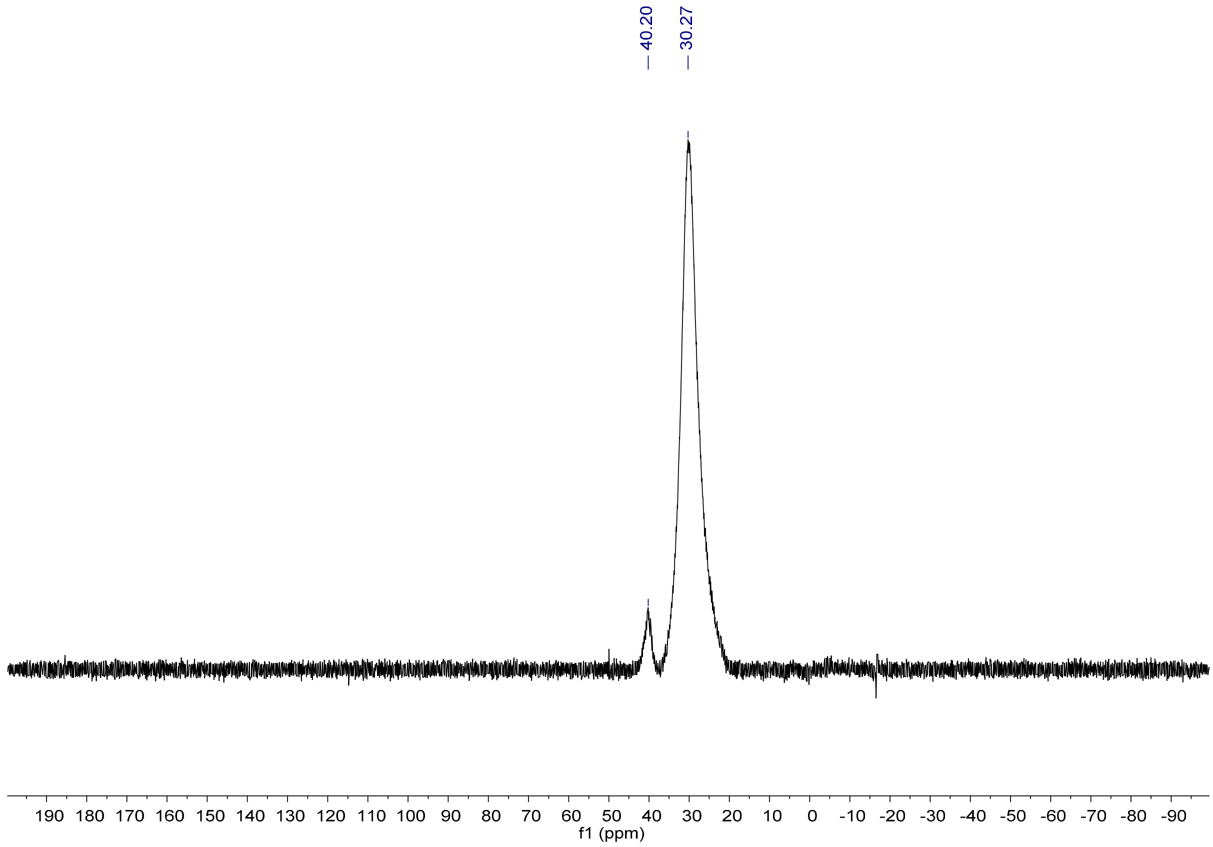

In situ ^{11}B-NMR (160 MHz, CDCl3, 298 K, 0.1 M) spectrum of **2a**.

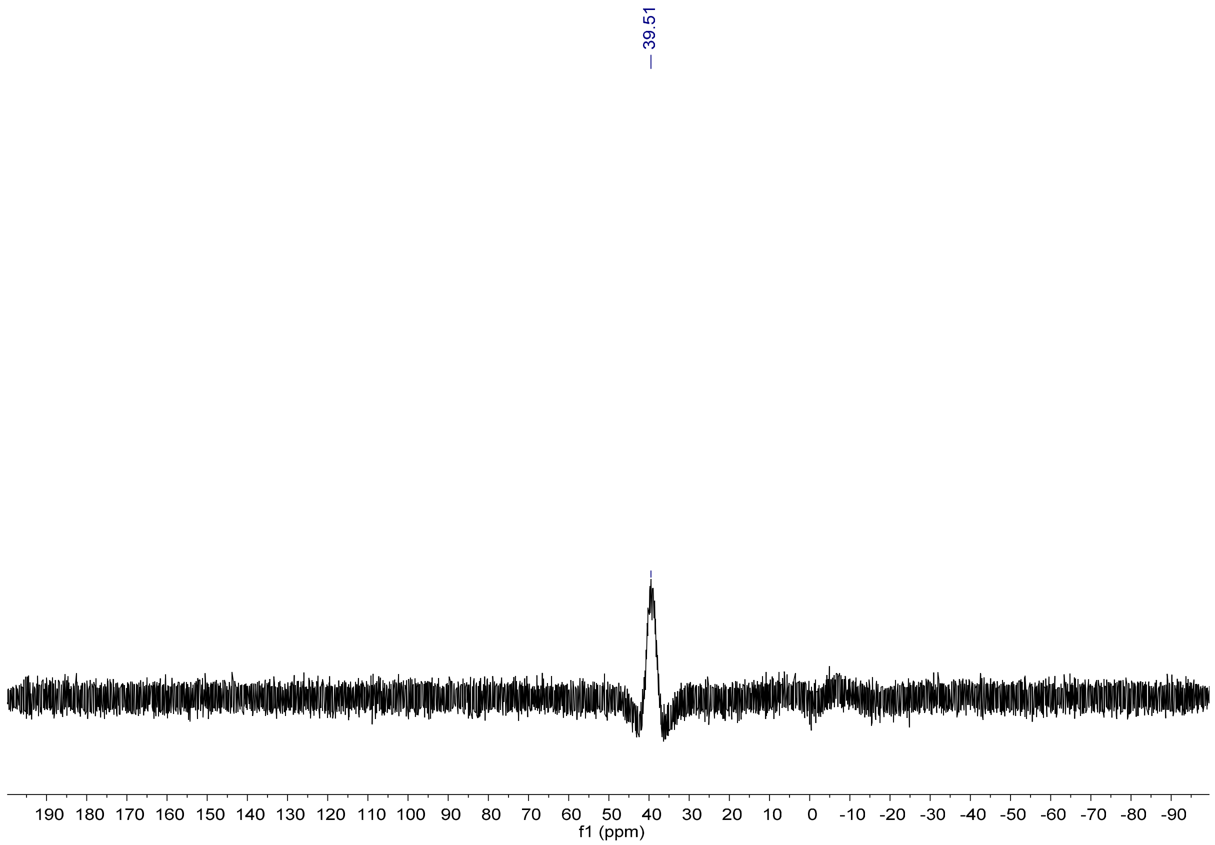

In situ ^{11}B-NMR (160 MHz, CDCl3, 298 K, 0.04 M) spectrum of **2a**.

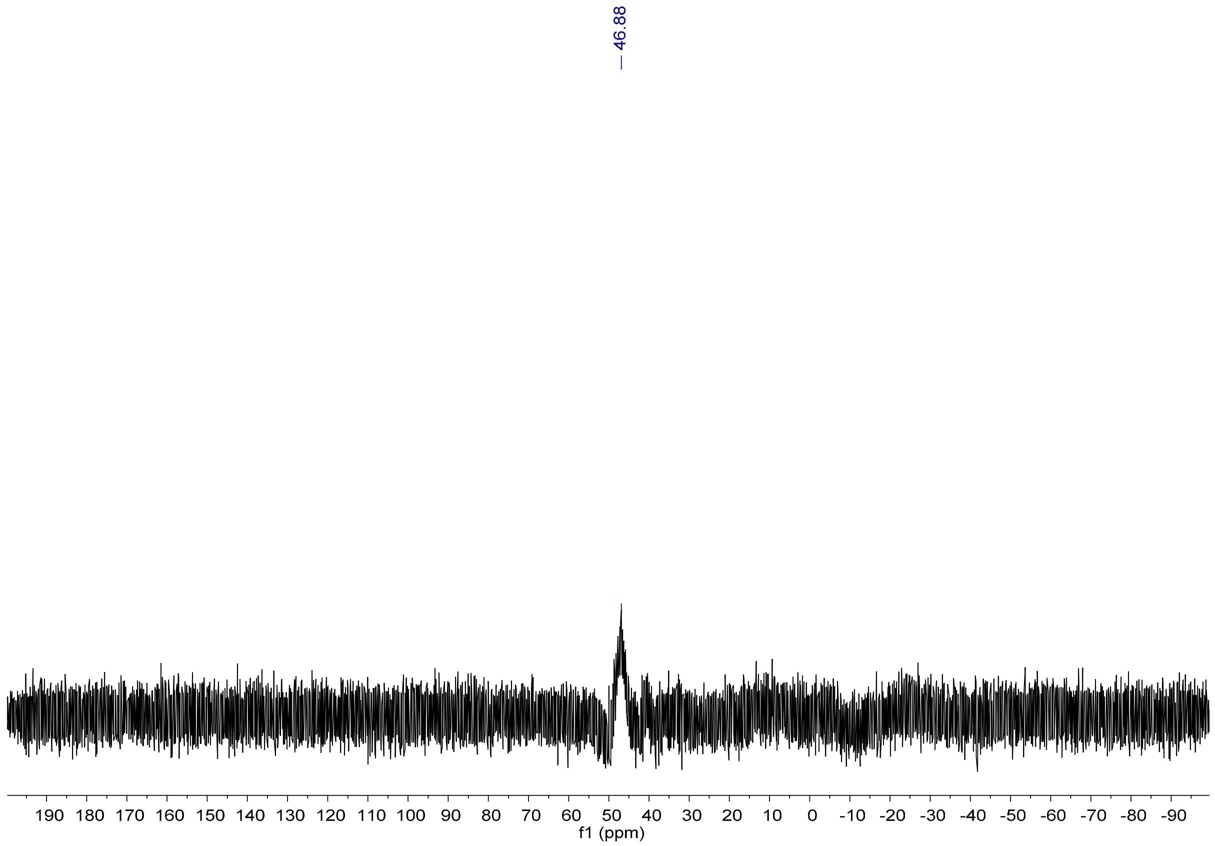

In situ ^{11}B-NMR (160 MHz, $CDCl_3$, 298 K) spectra of **2a** across concentrations 0.04–0.4 M.

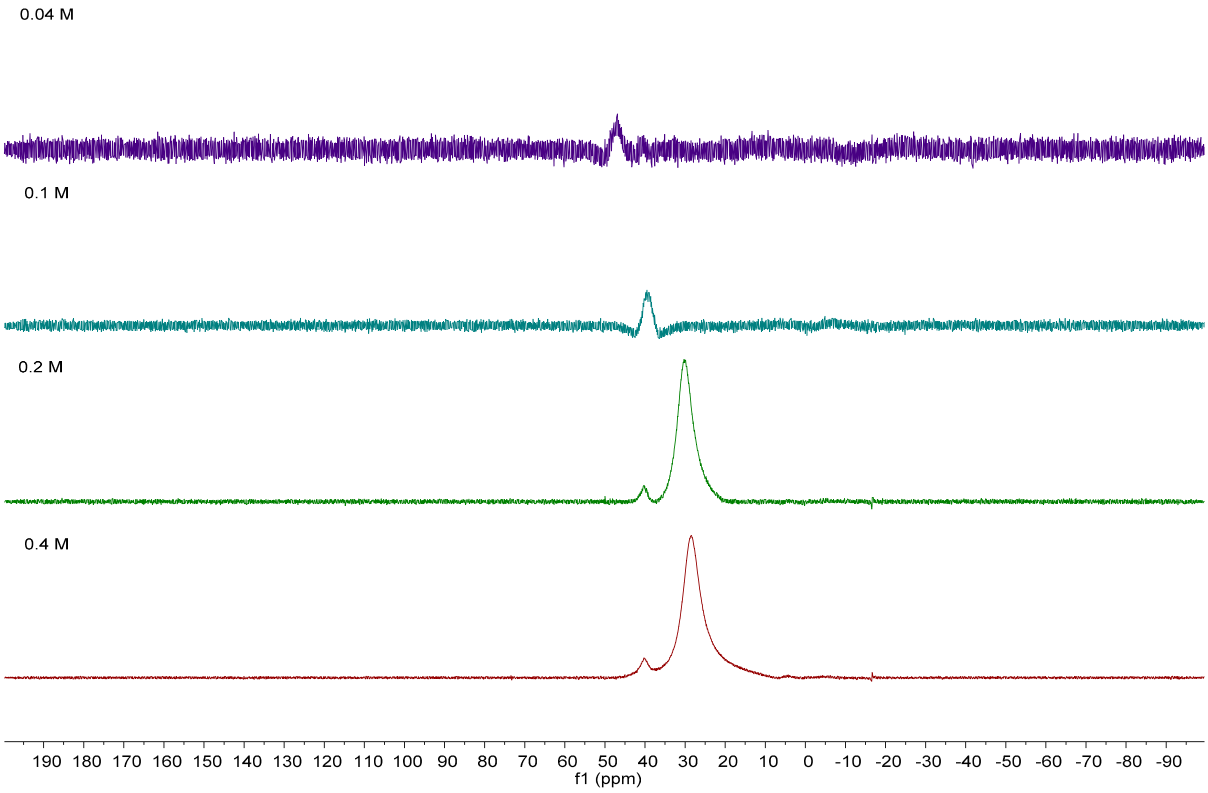

^{19}F-NMR (283 MHz, $CDCl_3$, 298 K) spectrum of **2a** crystals.

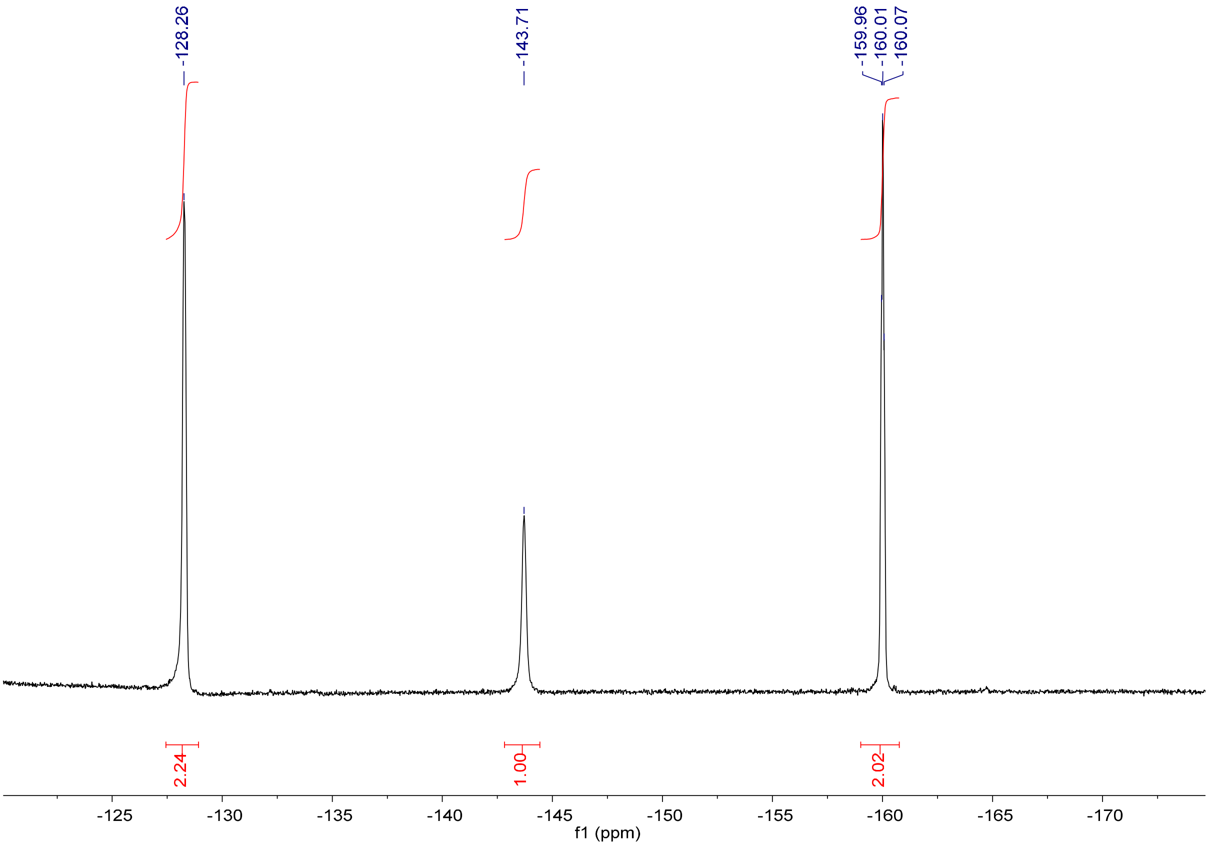

In situ ^{19}F-NMR (283 MHz, $CDCl_3$, 298 K, 0.4 M) spectrum of **2a**.

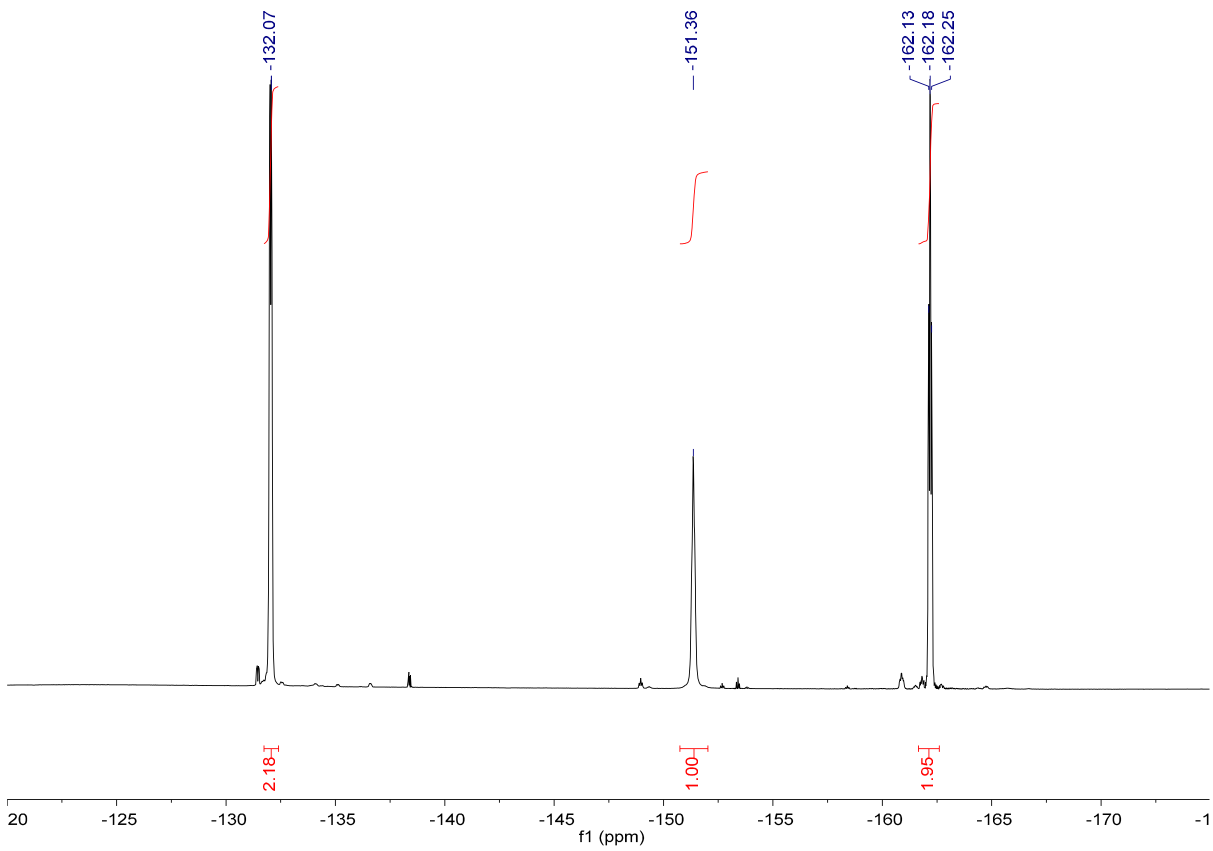

In situ ^{19}F-NMR (283 MHz, $CDCl_3$, 298 K, 0.2 M) spectrum of **2a**.

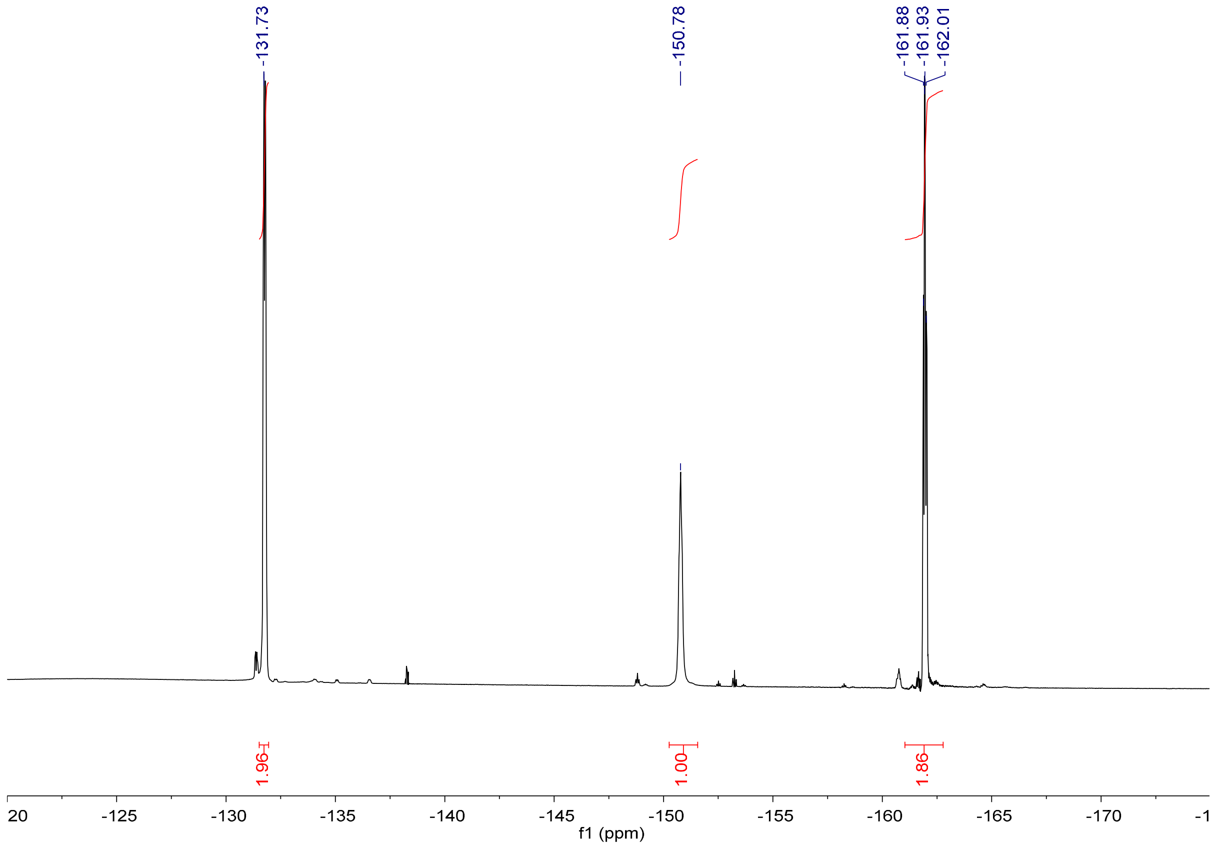

In situ ^{19}F-NMR (283 MHz, $CDCl_3$, 298 K, 0.1 M) spectrum of **2a**.

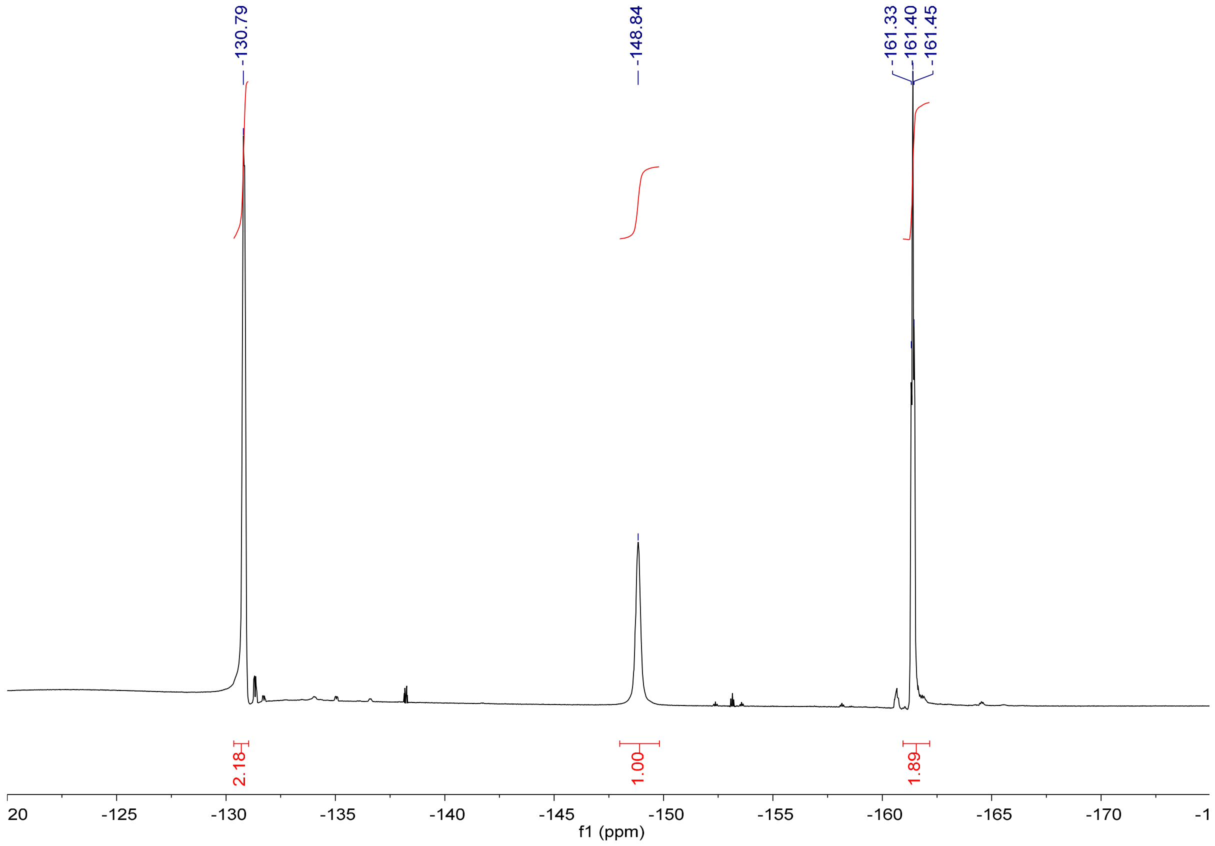

In situ ^{19}F-NMR (283 MHz, $CDCl_3$, 298 K, 0.04 M) spectrum of **2a**.

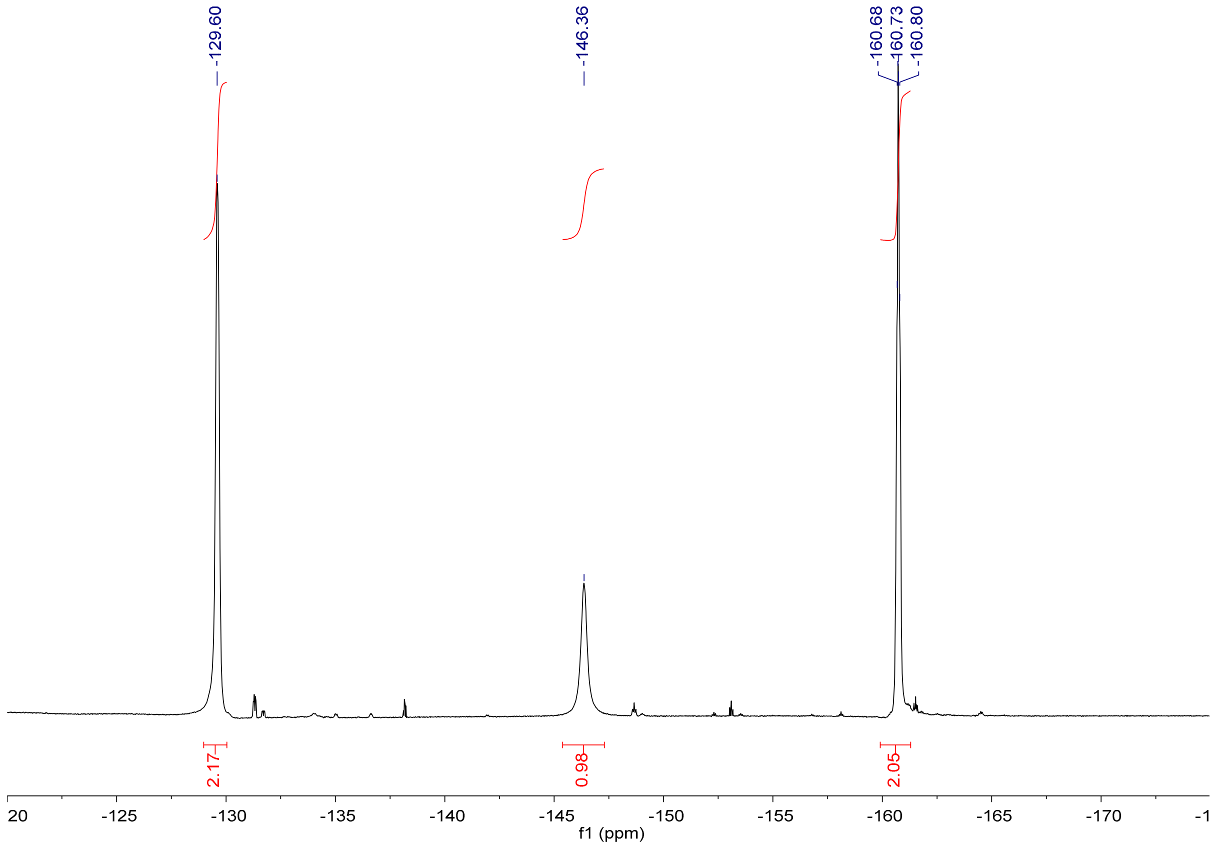

In situ ^{19}F-NMR (283 MHz, $CDCl_3$, 298 K) spectra of **2a** across concentrations 0.04–0.4 M

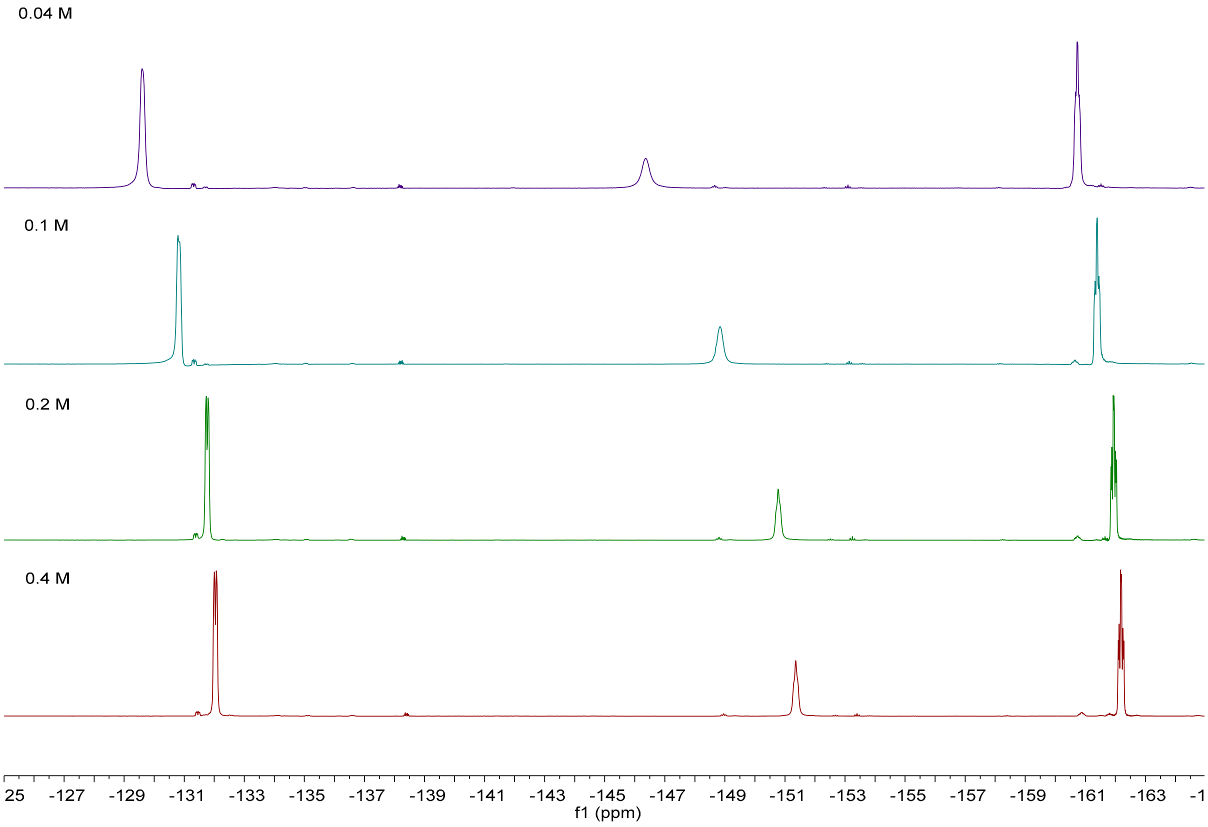

^{1}H-NMR (500 MHz, $CDCl_3$, 298K) spectrum of **2b** crystals.

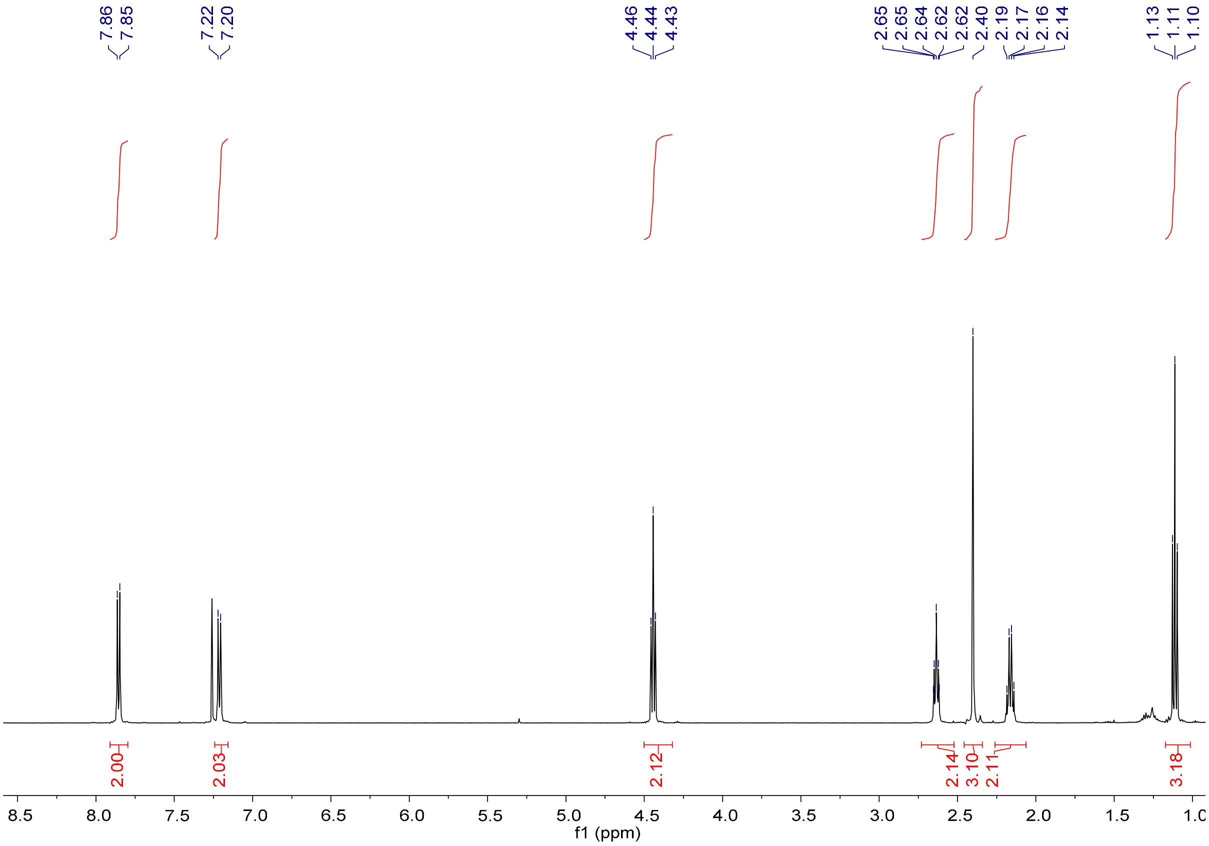

In situ ^{1}H-NMR (500 MHz, $CDCl_3$, 298K, 0.4 M) spectrum of **2b**.

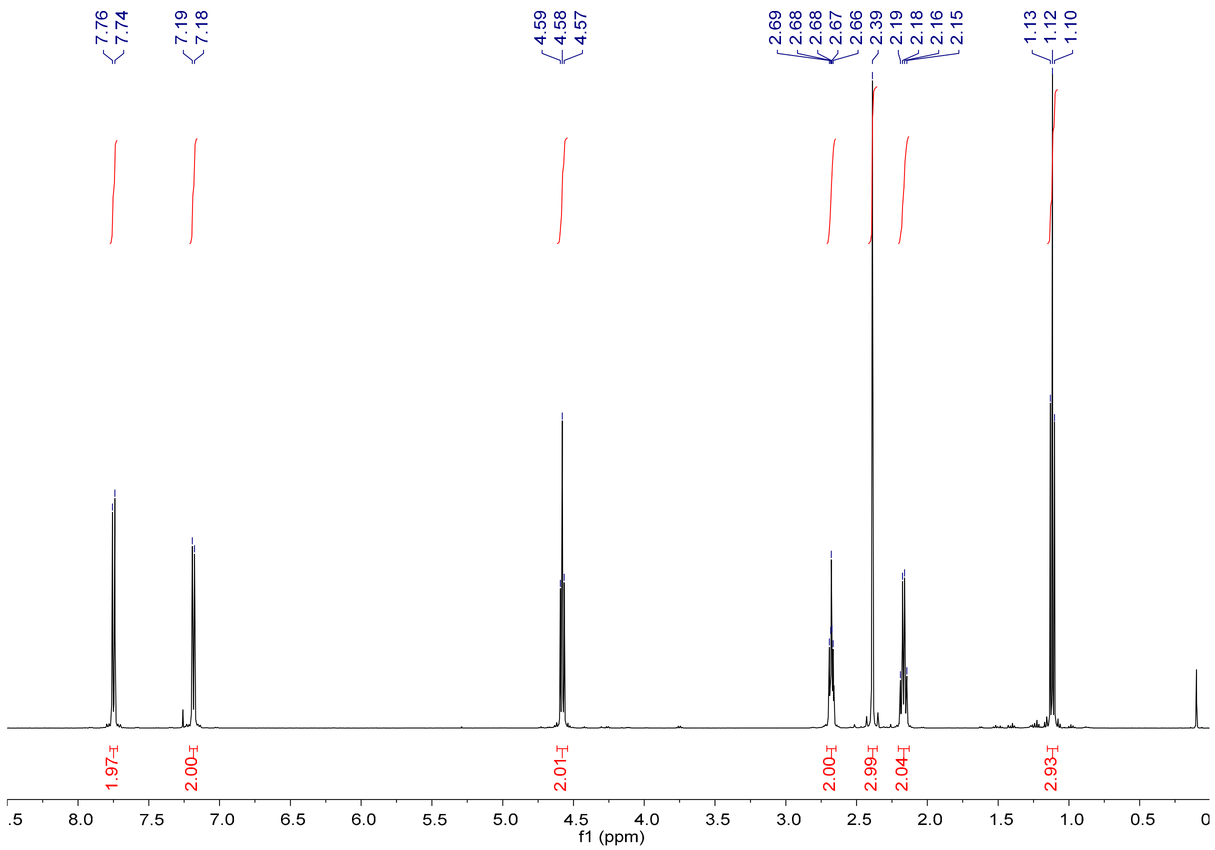

In situ ^{1}H-NMR (500 MHz, $CDCl_3$, 298K, 0.2 M) spectrum of **2b**.

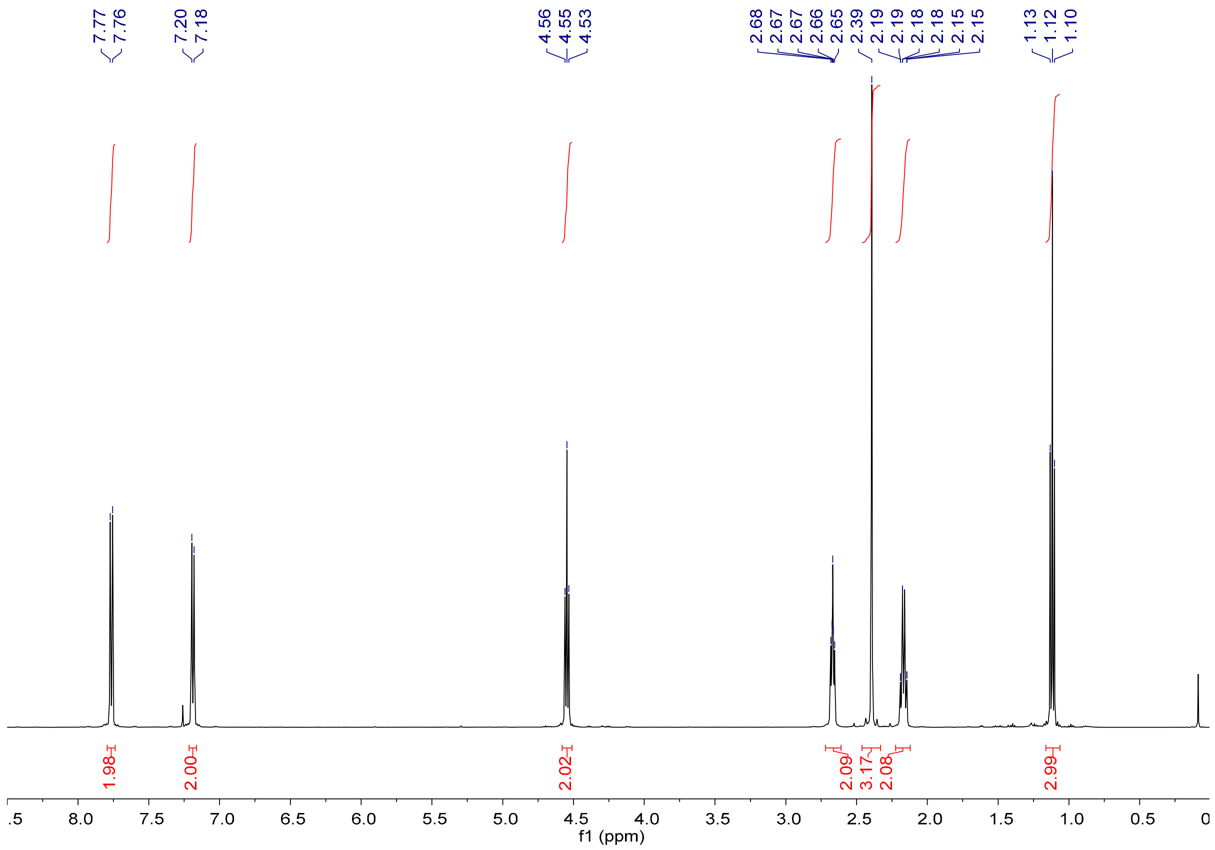

In situ ^{1}H-NMR (500 MHz, $CDCl_3$, 298K, 0.1 M) spectrum of **2b**.

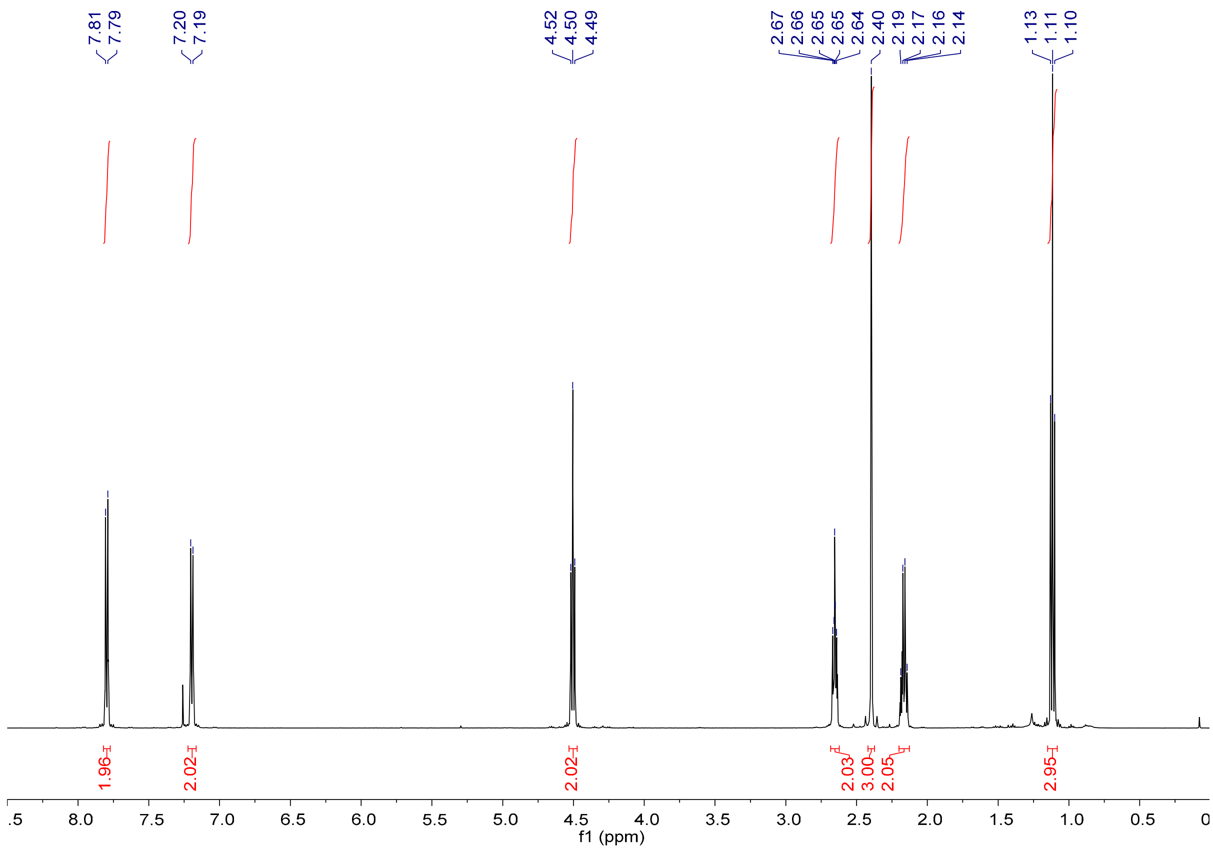

In situ ^{1}H-NMR (500 MHz, $CDCl_3$, 298K, 0.04 M) spectrum of **2b**.

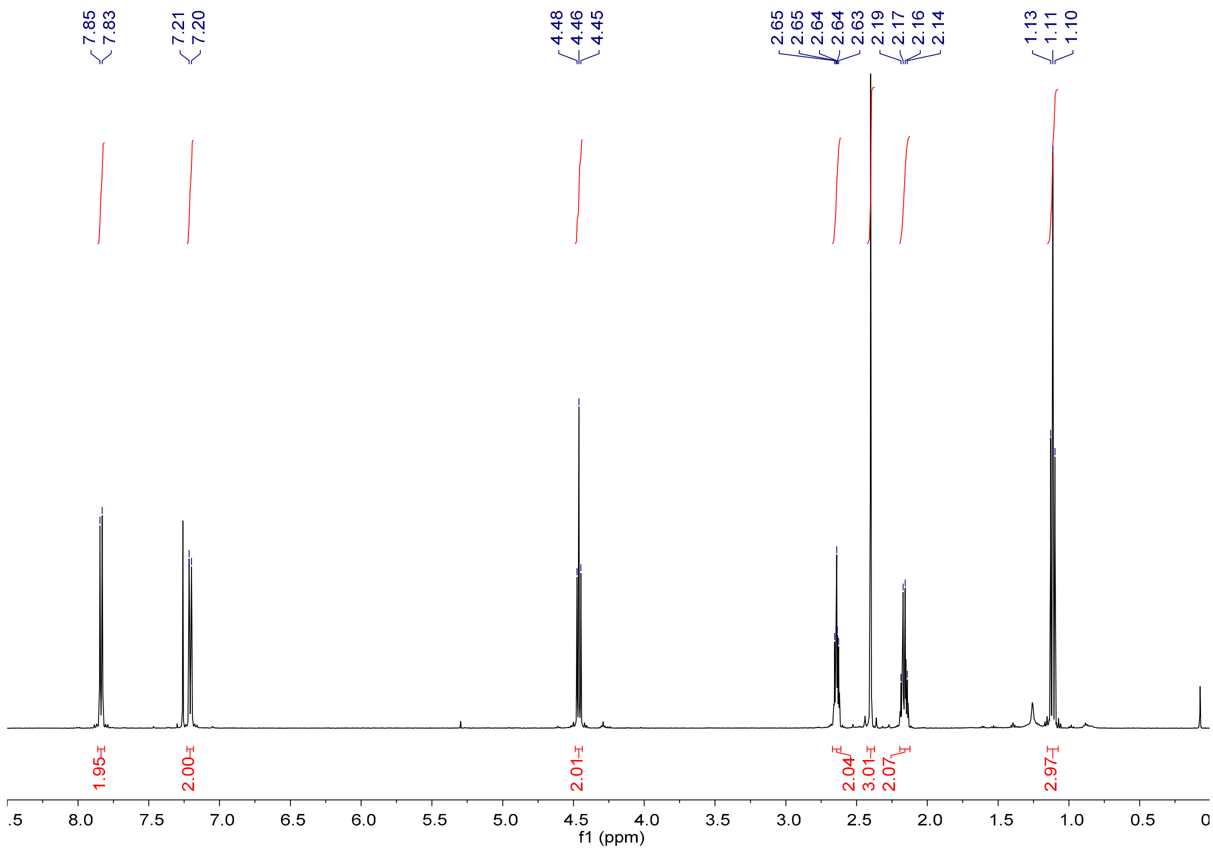

In situ ^{1}H-NMR (500 MHz, $CDCl_3$, 298K) spectra of **2b** across concentrations 0.04–0.4 M.

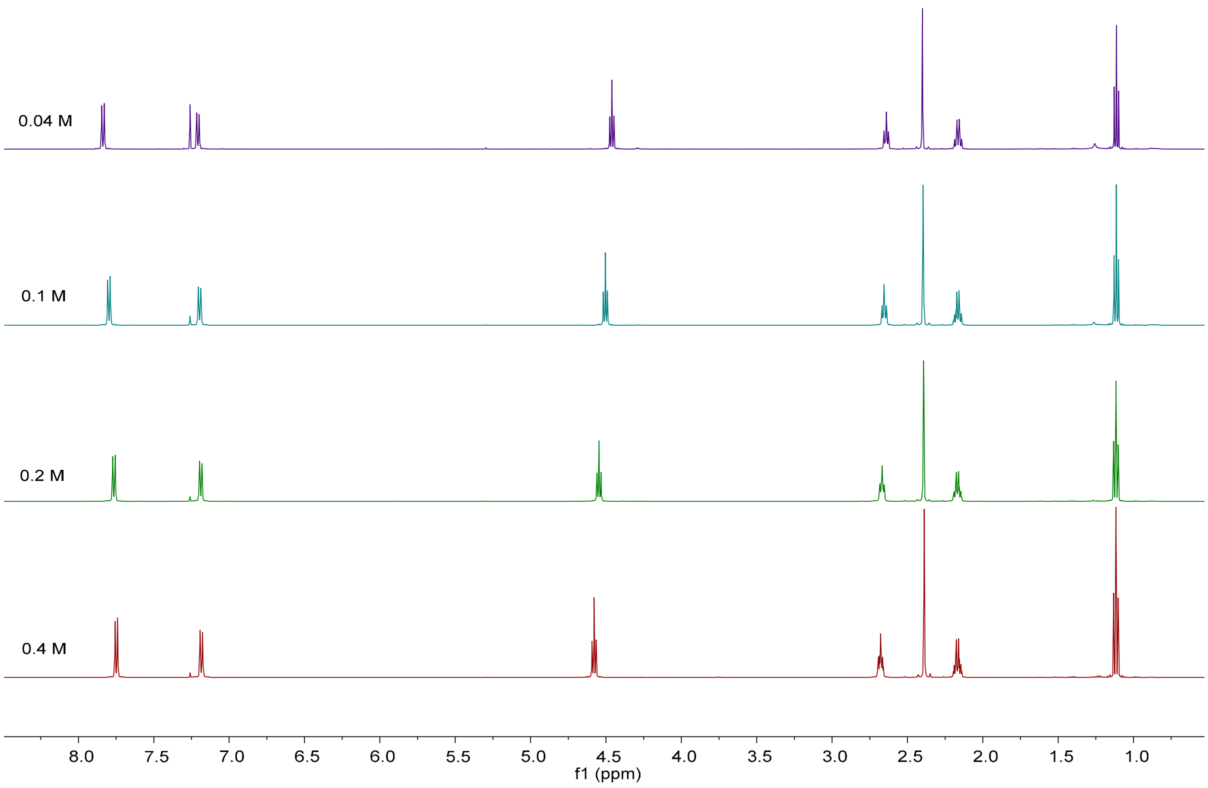

In situ ^{1}H-NMR (500 MHz, $CDCl_3$, 298K) spectra of **2b**, (expansion of C<u>H</u>$_2$O(CO)-) across concentrations 0.04–0.4 M.

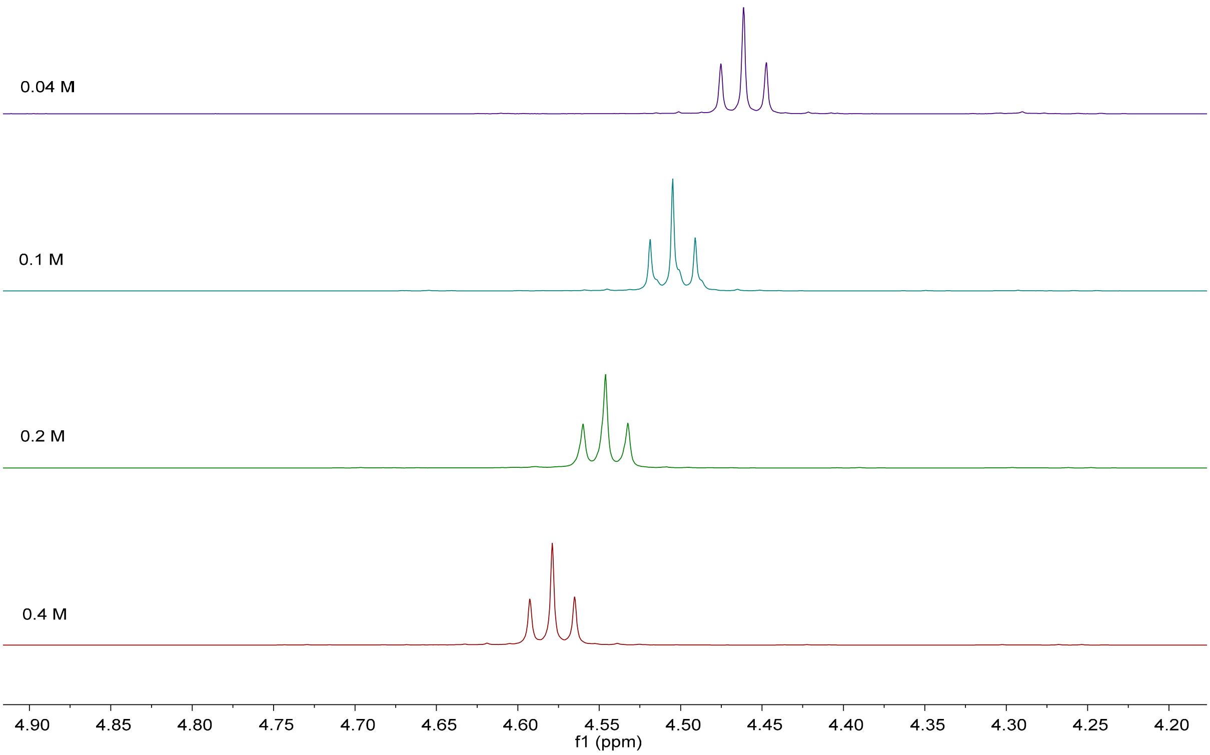

In situ ^{13}C-NMR (125 MHz, $CDCl_3$, 298K, 0.2 M) spectrum of **2b**.

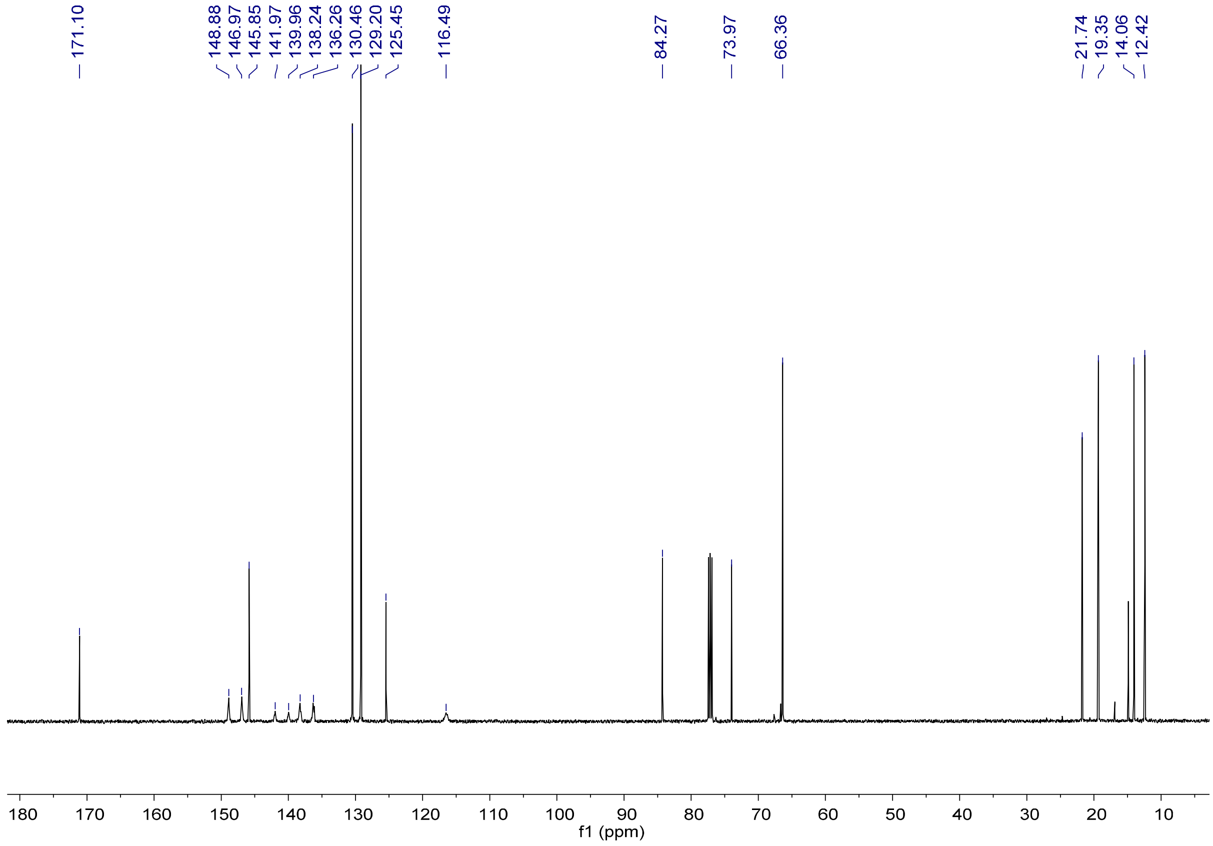

In situ ^{11}B-NMR (160 MHz, $CDCl_3$, 298 K, 0.4 M) spectrum of **2b**.

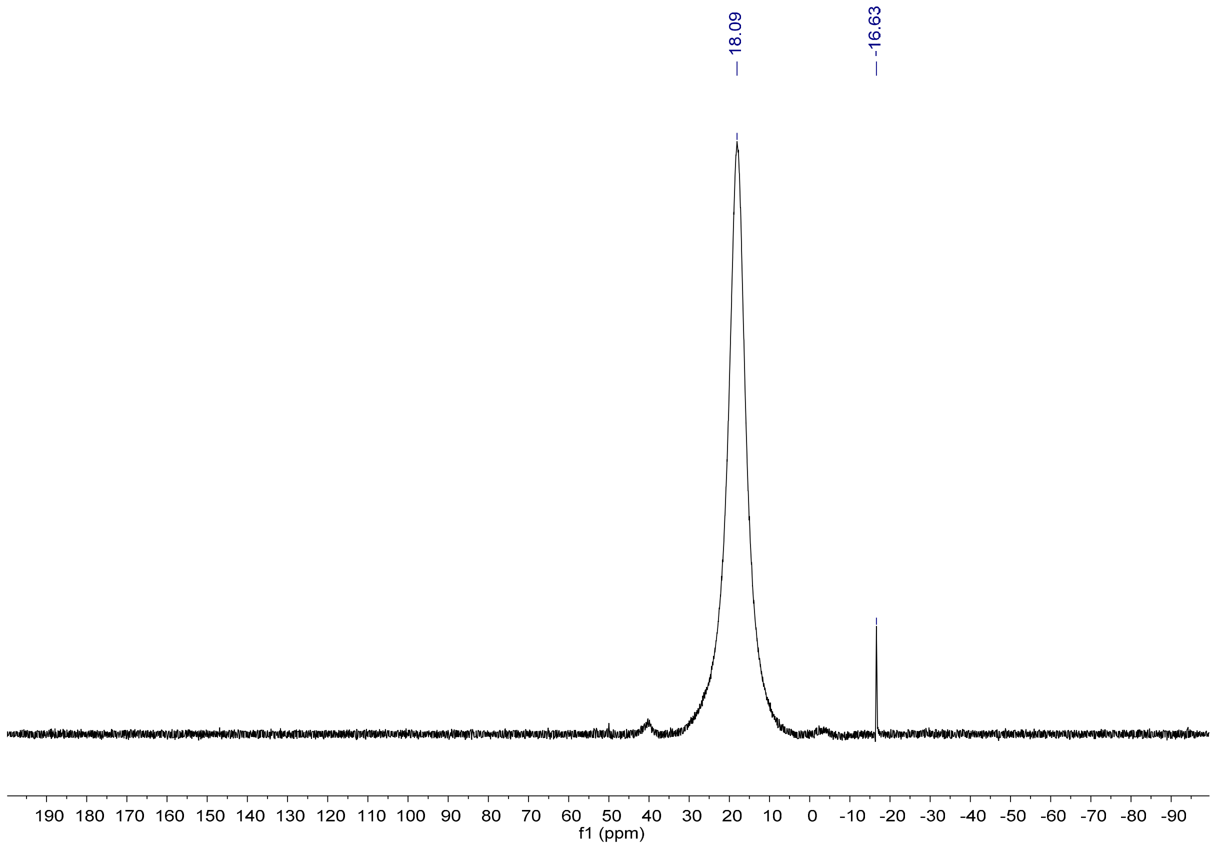

In situ ^{11}B-NMR (160 MHz, $CDCl_3$, 298 K, 0.2 M) spectrum of **2b**.

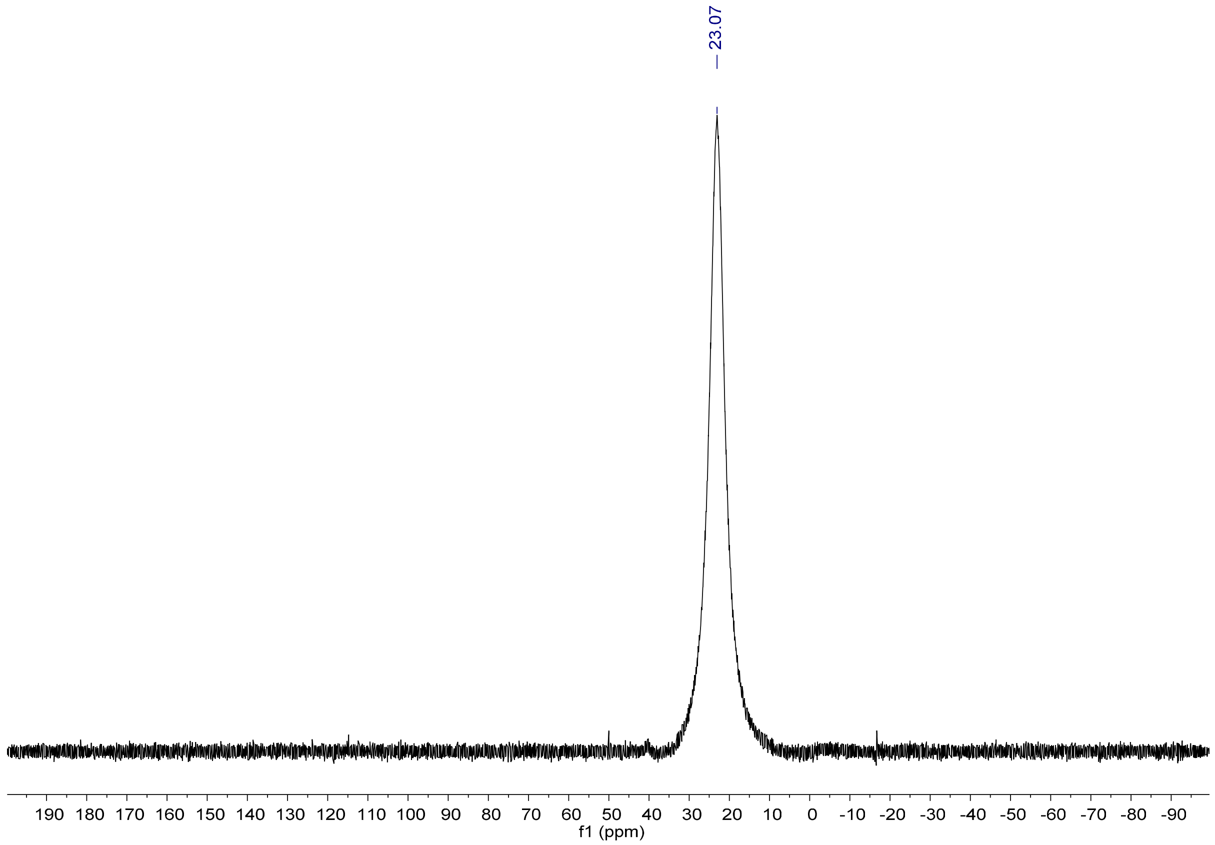

In situ ^{11}B-NMR (160 MHz, $CDCl_3$, 298 K, 0.1 M) spectrum of **2b**.

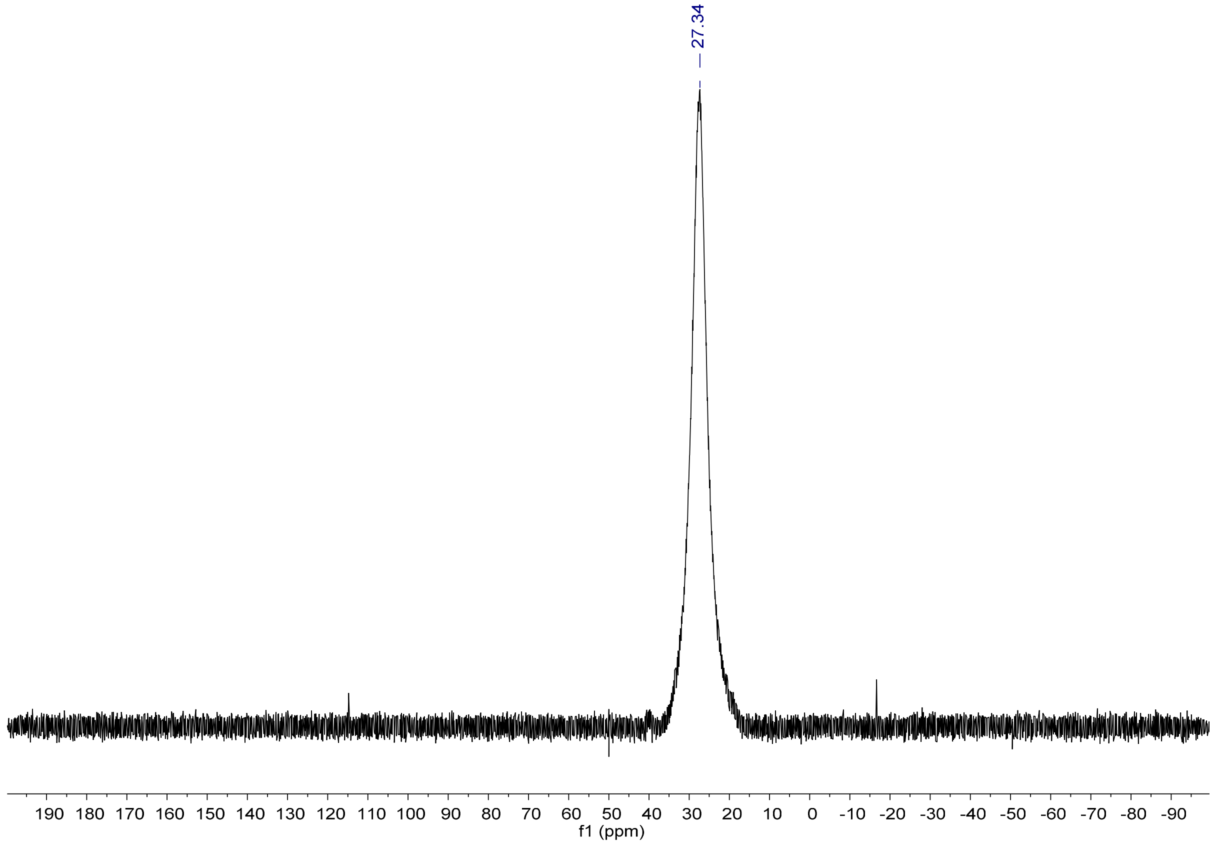

In situ ^{11}B-NMR (160 MHz, $CDCl_3$, 298 K, 0.04 M) spectrum of **2b**.

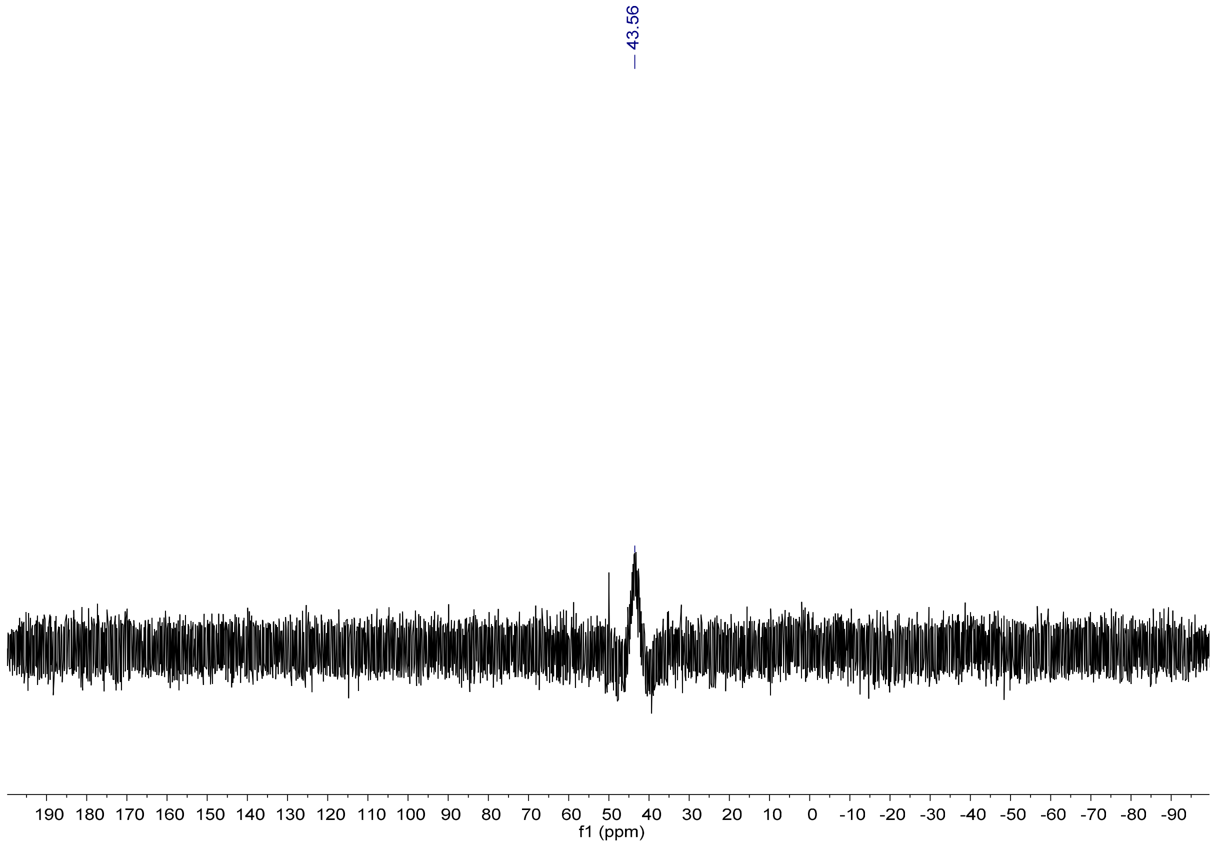

In situ ^{11}B-NMR (160 MHz, $CDCl_3$, 298 K) spectra of **2a** across concentrations 0.04–0.4 M.

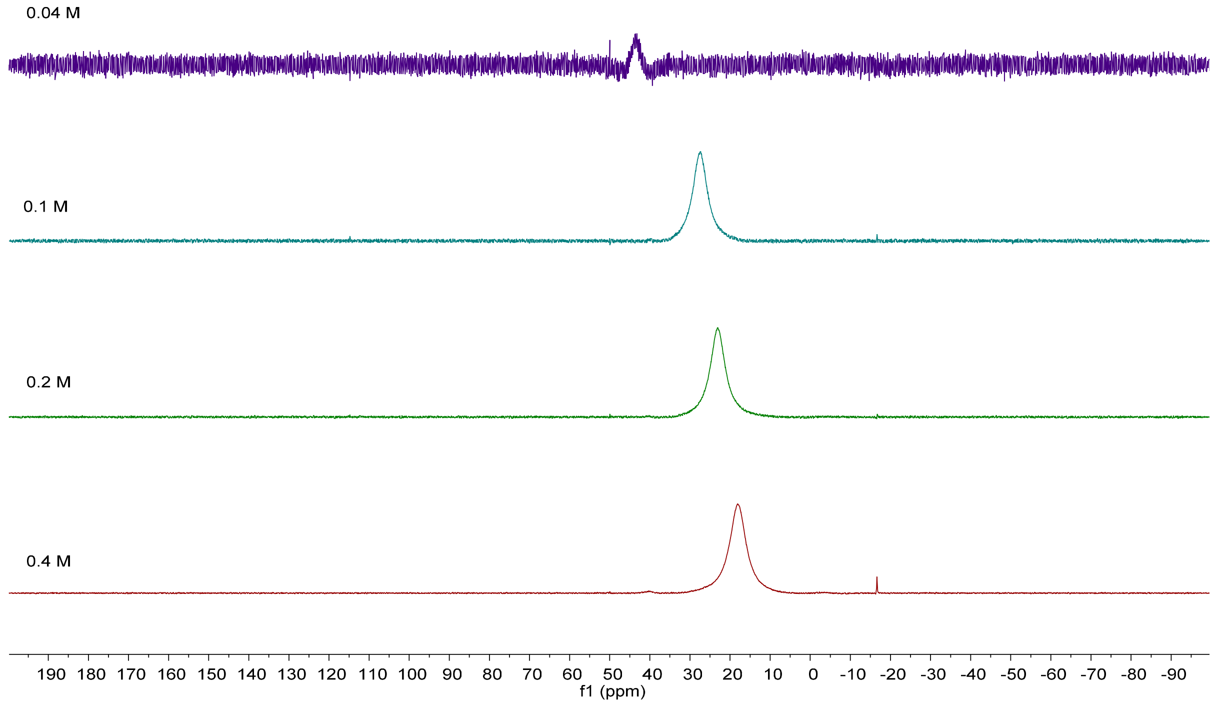

^{19}F-NMR (283 MHz, $CDCl_3$, 298 K) spectrum of **2b** crystals.

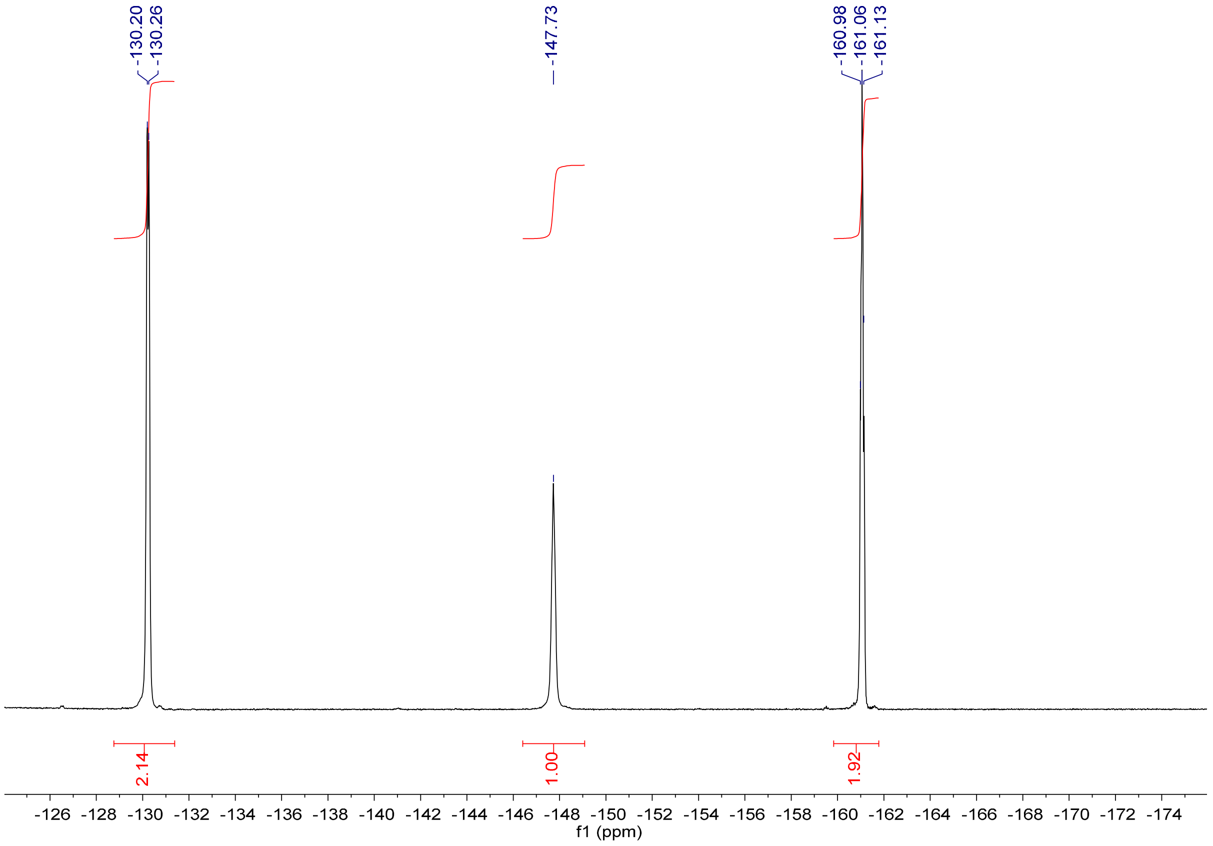

In situ ^{19}F-NMR (283 MHz, $CDCl_3$, 298 K, 0.4 M) spectrum of **2b**.

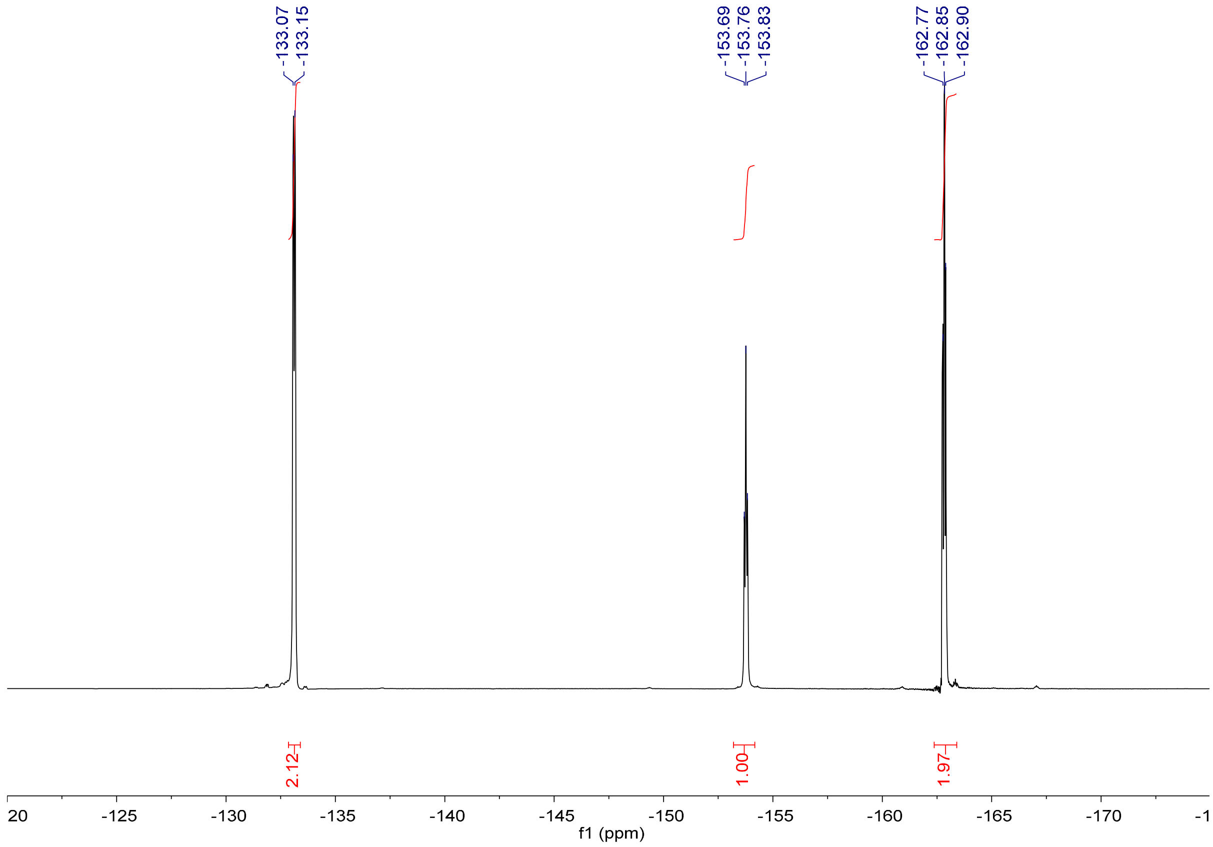

In situ ^{19}F-NMR (283 MHz, $CDCl_3$, 298 K, 0.2 M) spectrum of **2b**.

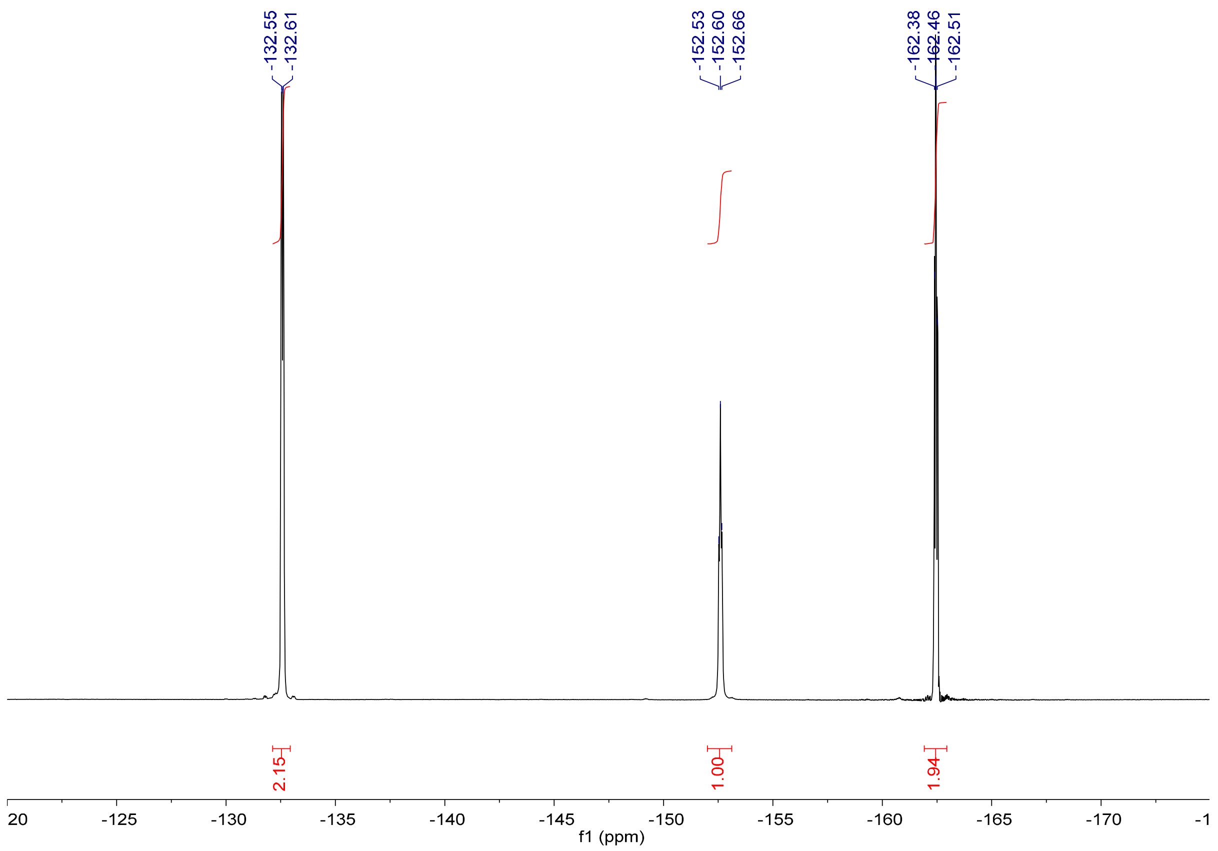

In situ ^{19}F-NMR (283 MHz, $CDCl_3$, 298 K, 0.1 M) spectrum of **2b**.

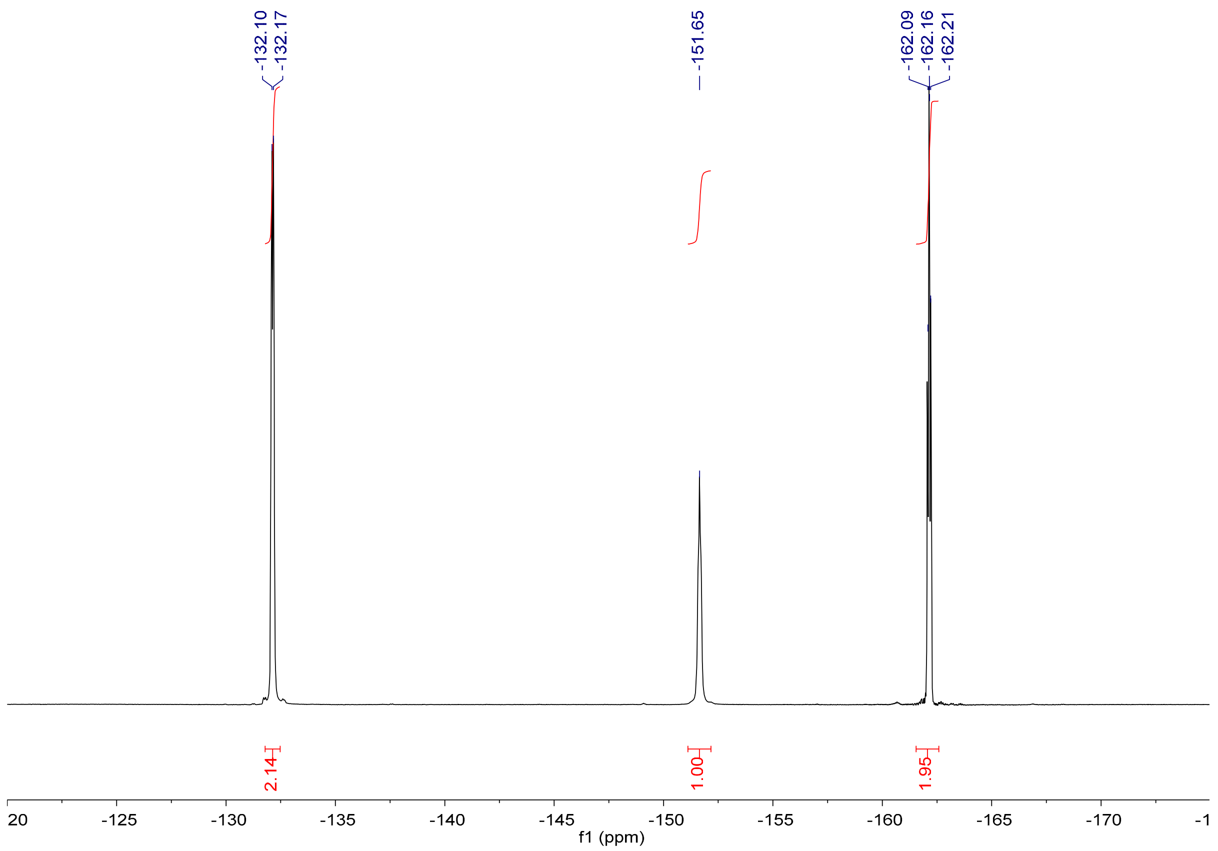

In situ ^{19}F-NMR (283 MHz, $CDCl_3$, 298 K, 0.04 M) spectrum of **2b**.

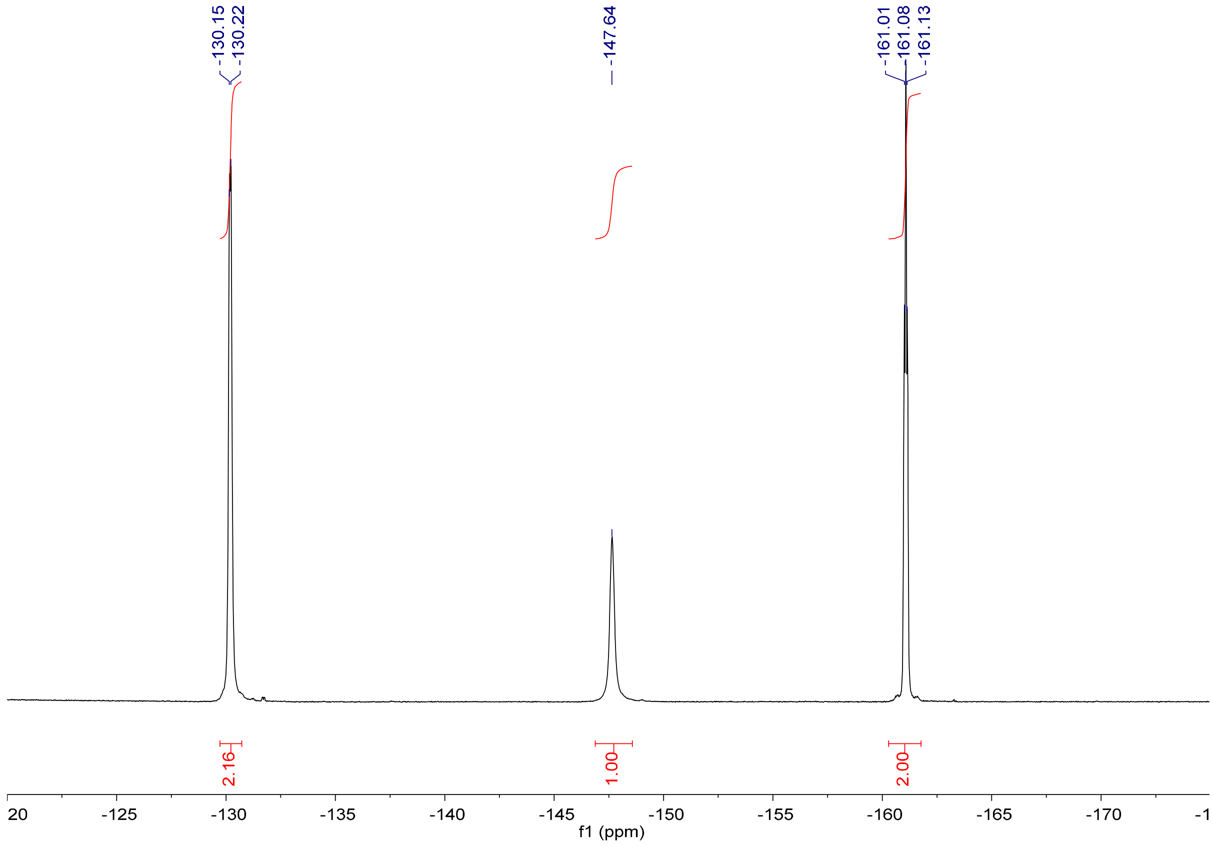

In situ ^{19}F-NMR (283 MHz, $CDCl_3$, 298 K) spectra of **2b** across concentrations 0.04–0.4 M

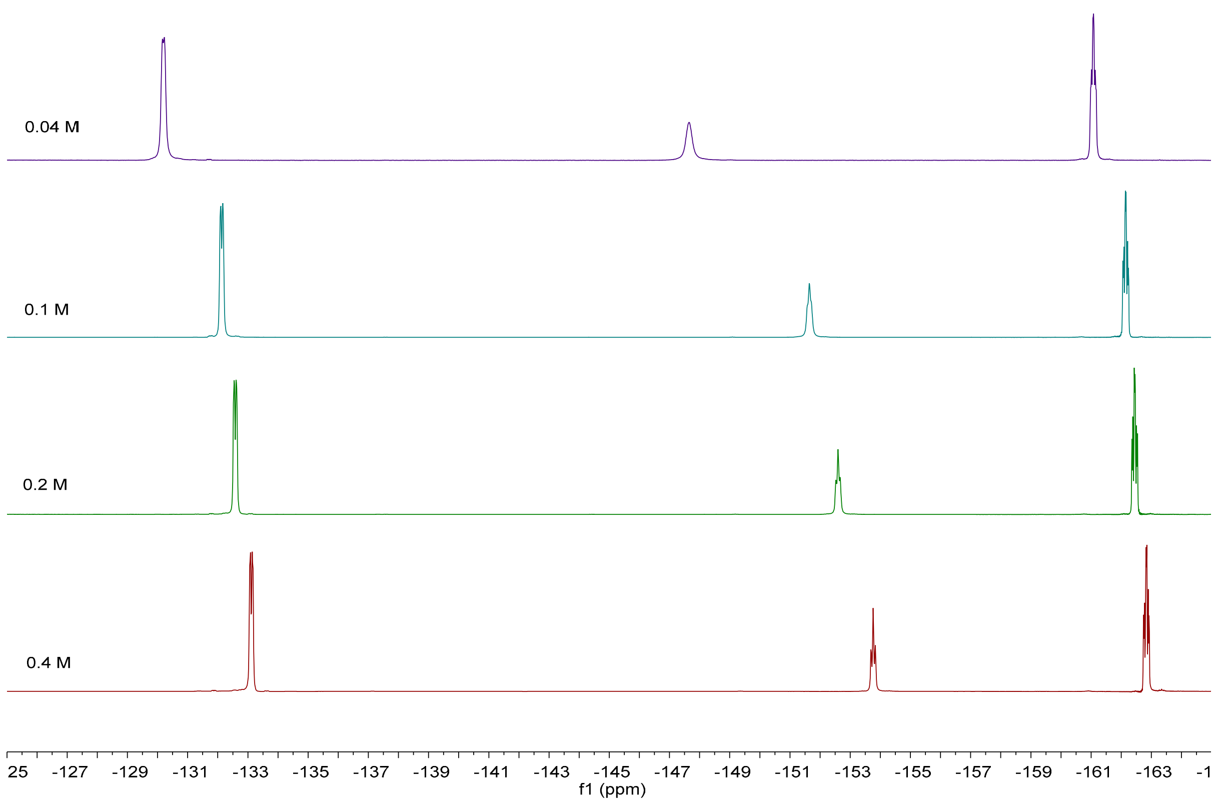

^{1}H-NMR (500 MHz, $CDCl_3$, 298K) spectrum of **2c** crystals.

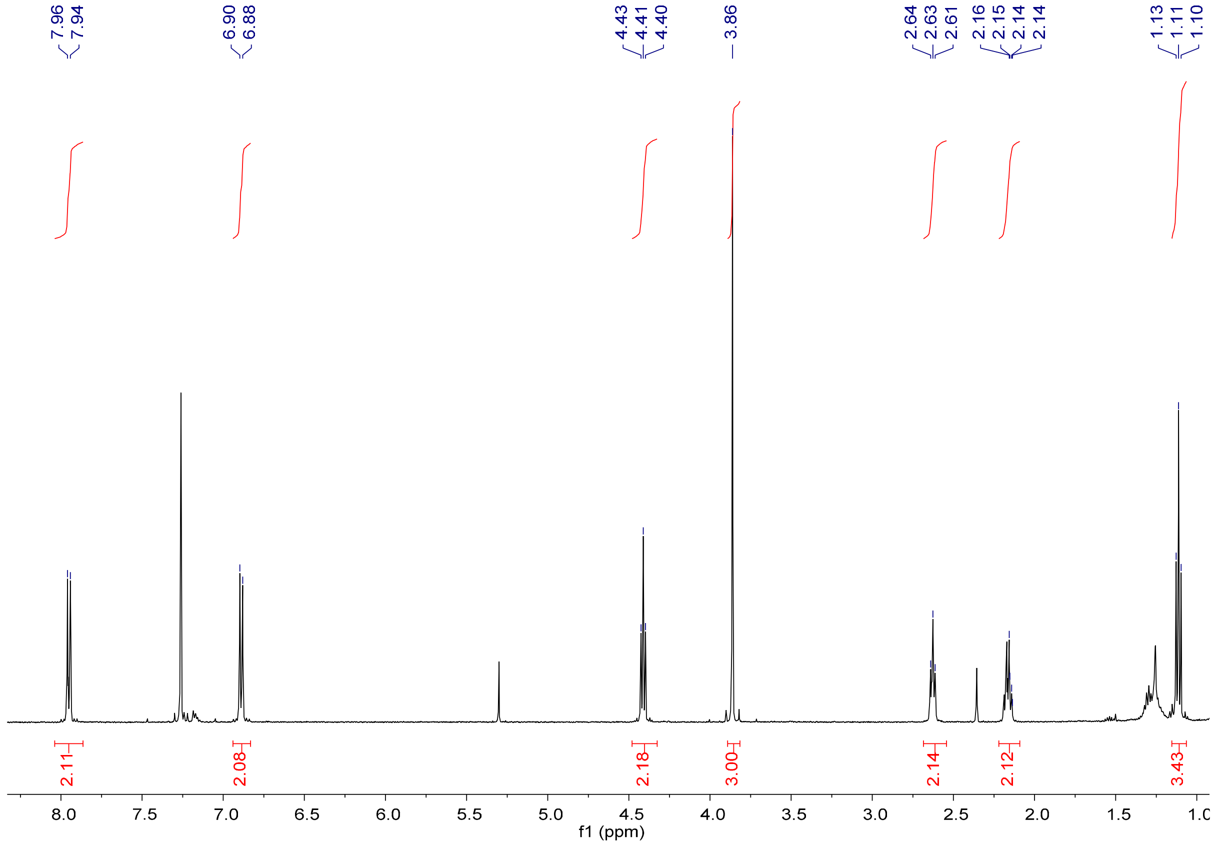

In situ ^{1}H-NMR (500 MHz, $CDCl_3$, 298K, 0.4 M) spectrum of **2c**.

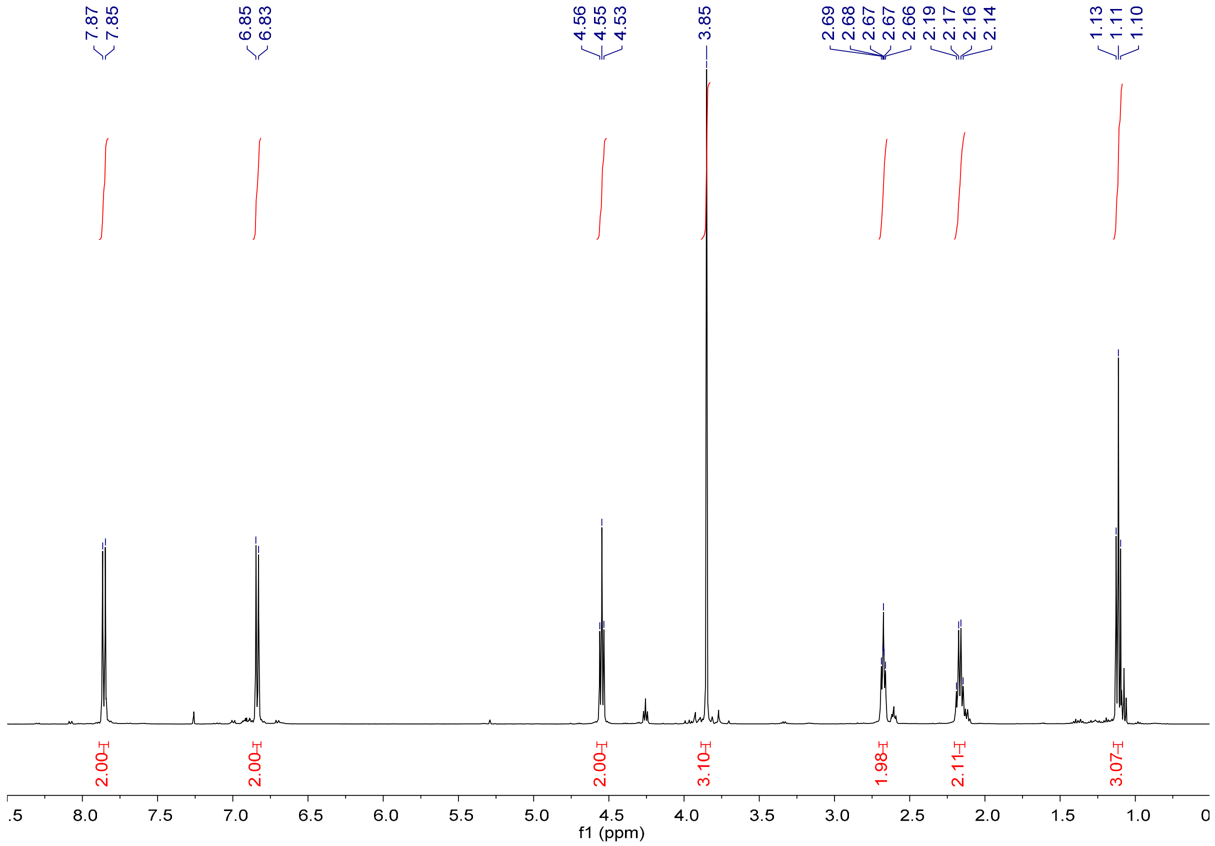

In situ ^{1}H-NMR (500 MHz, CDCl₃, 298K, 0.2 M) spectrum of **2c**.

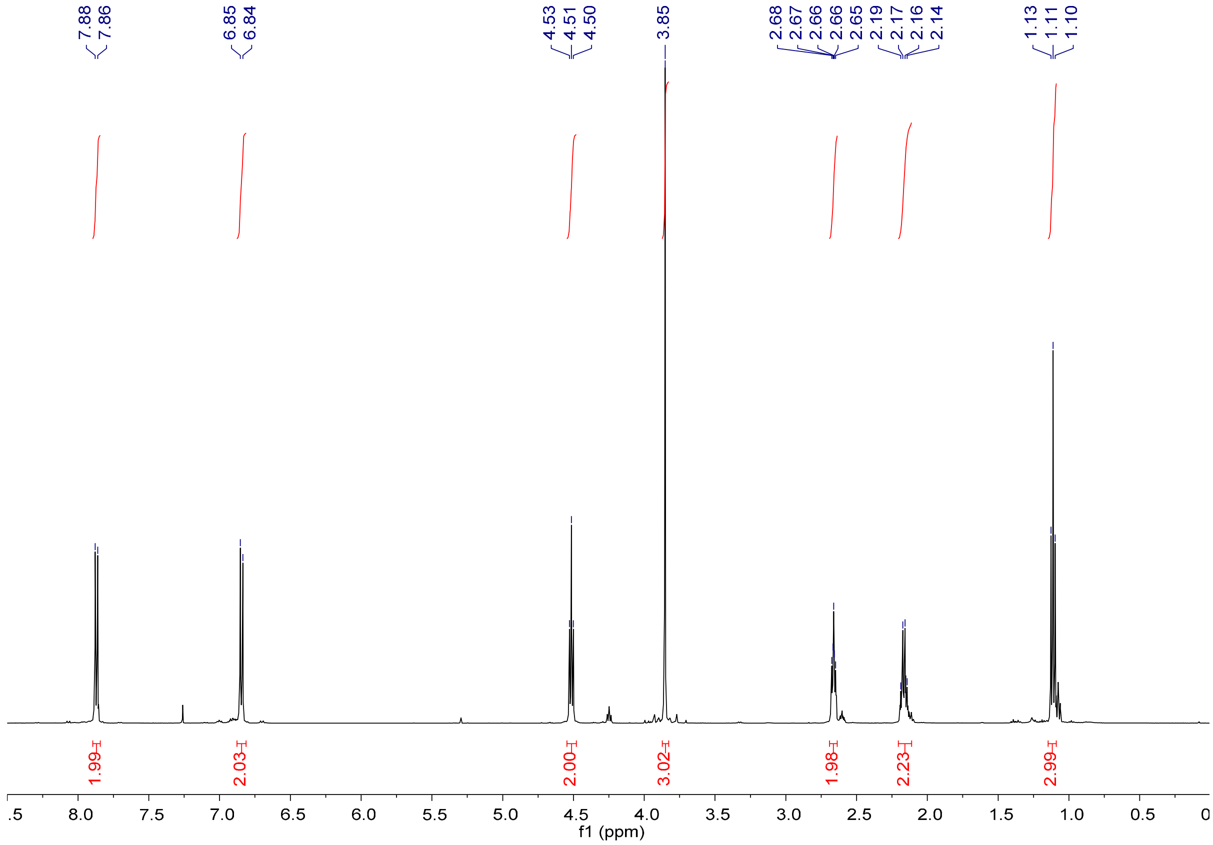

In situ ^{1}H-NMR (500 MHz, CDCl₃, 298K, 0.1 M) spectrum of **2c**.

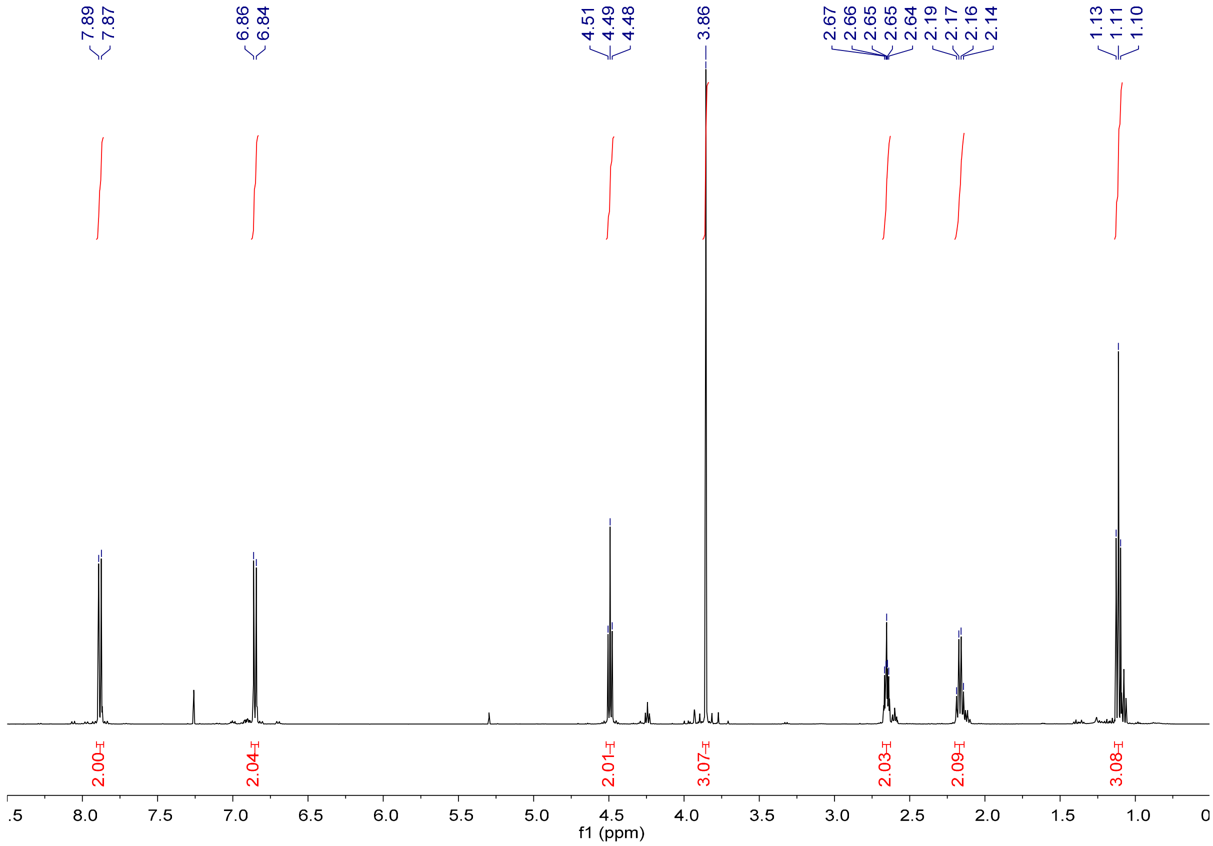

In situ ^{1}H-NMR (500 MHz, CDCl3, 298K, 0.04 M) spectrum of **2c**.

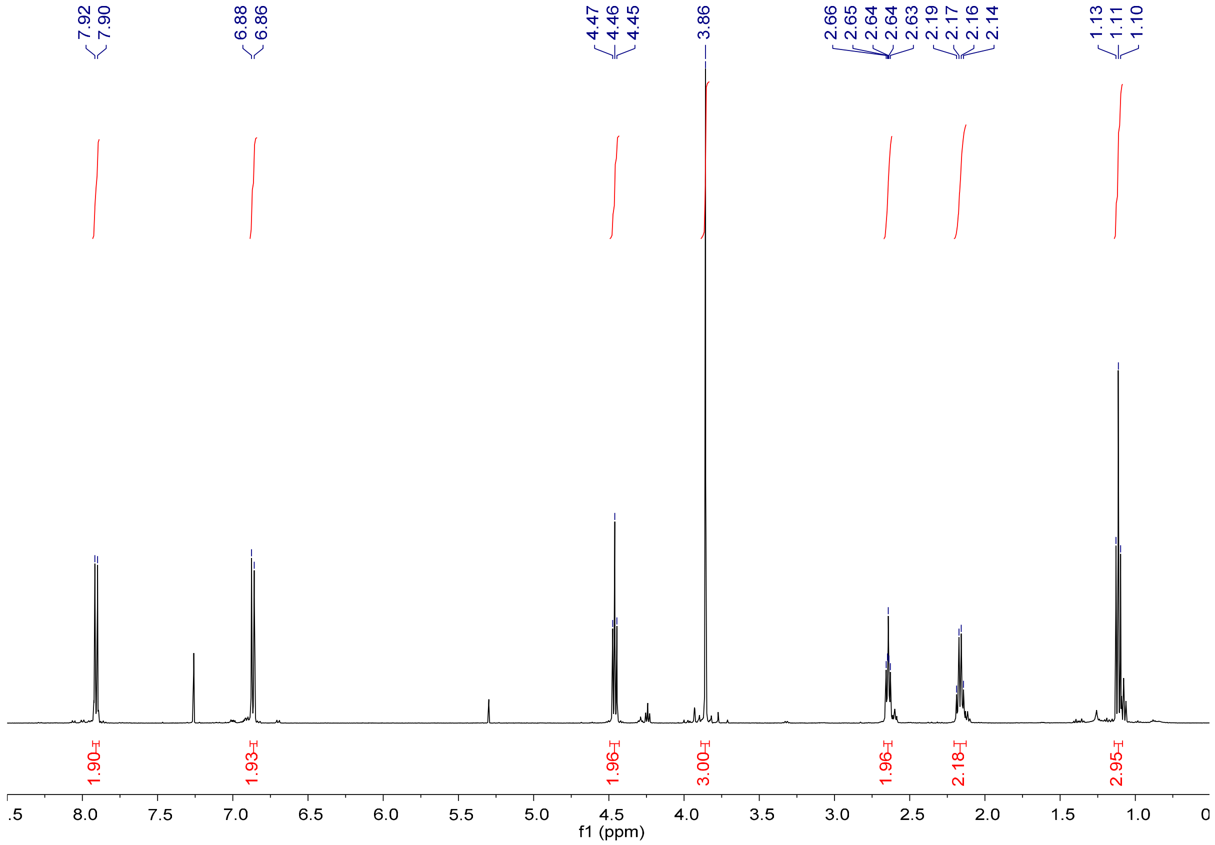

In situ ^{1}H-NMR (500 MHz, CDCl3, 298K) spectra of **2c** across concentrations 0.04–0.4 M.

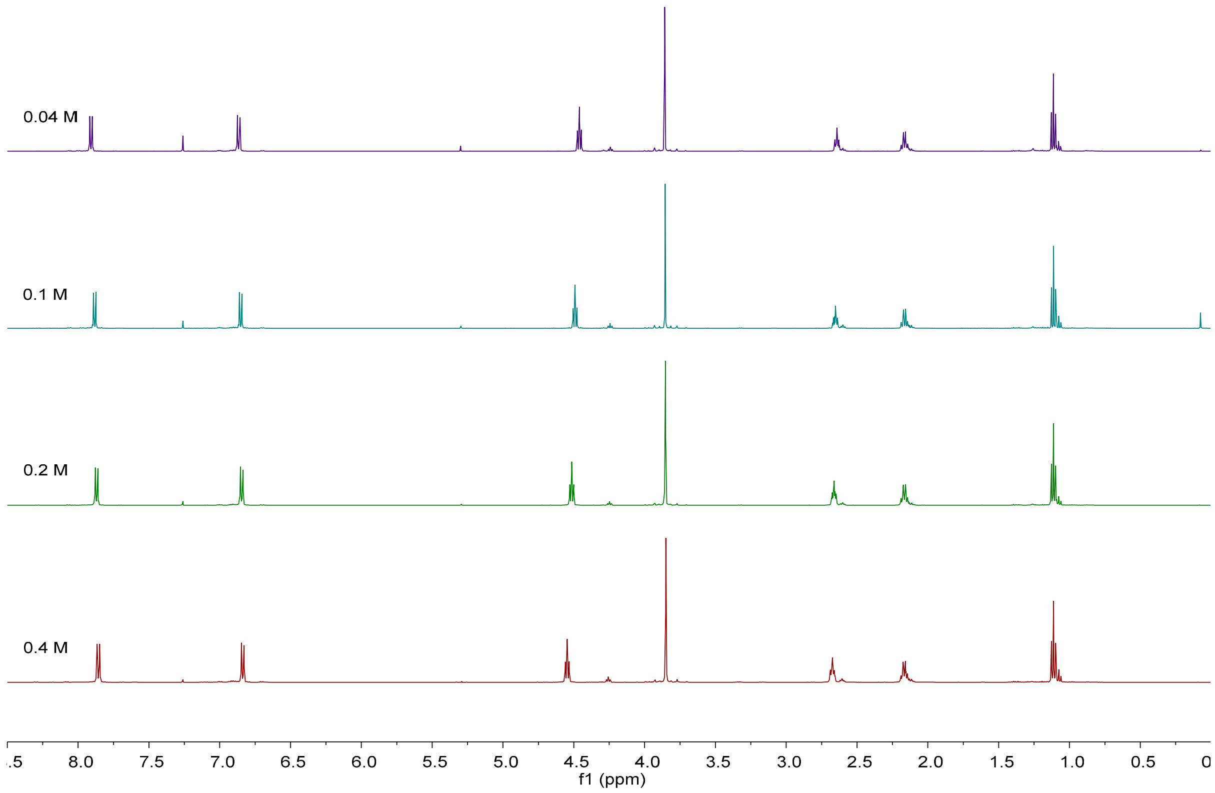

In situ ^{1}H-NMR (500 MHz, $CDCl_3$, 298K) spectra of **2c,** expansion of C<u>H</u>$_2$O(CO)-) across concentrations 0.04 –0.4 M.

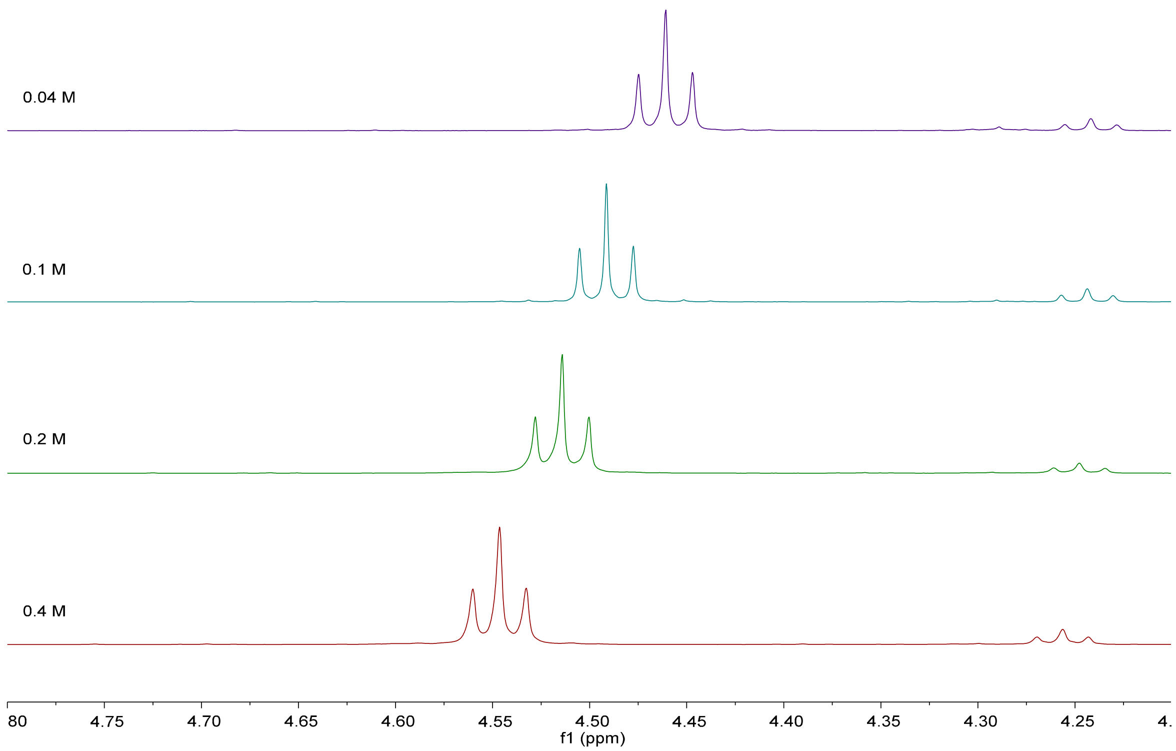

In situ ^{13}C-NMR (125 MHz, $CDCl_3$, 298K, 0.2 M) spectrum of **2c**

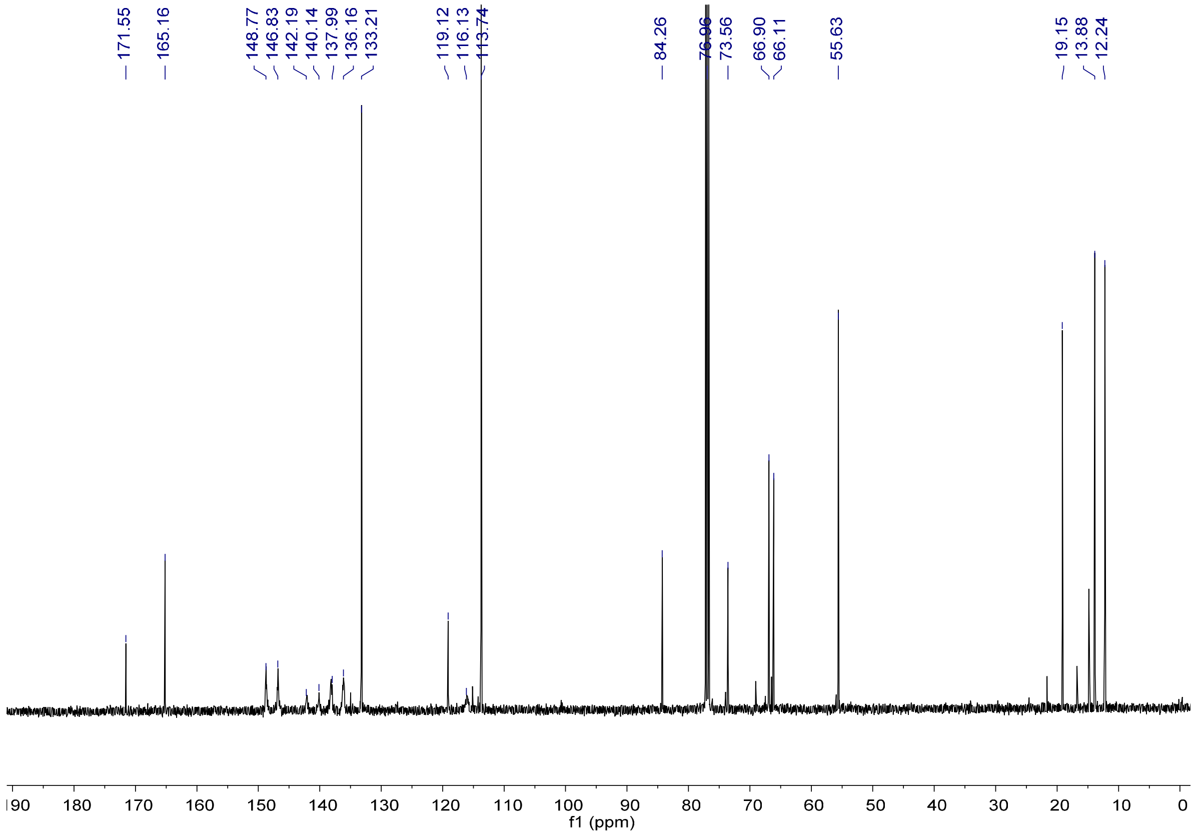

In situ ^{11}B-NMR (160 MHz, $CDCl_3$, 298 K, 0.4 M) spectrum of **2c**.

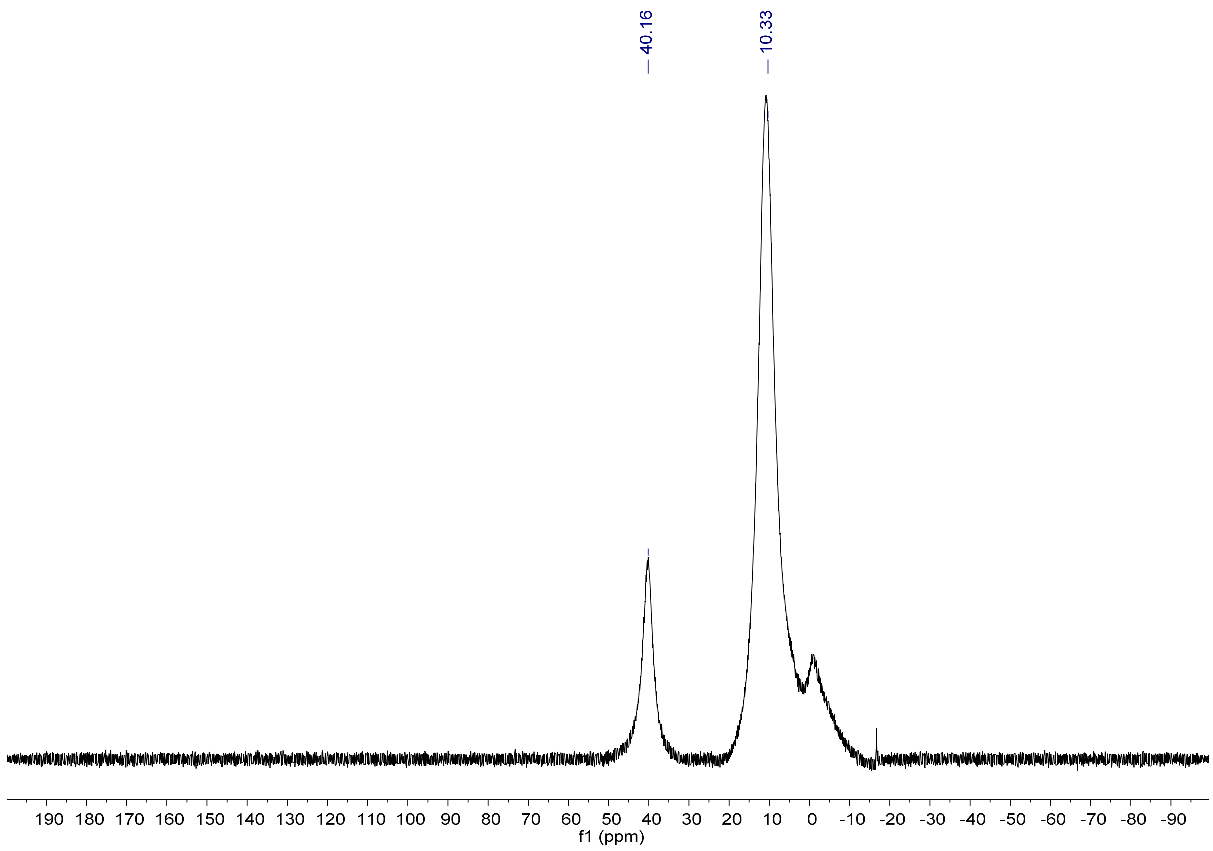

In situ ^{11}B-NMR (160 MHz, $CDCl_3$, 298 K, 0.2 M) spectrum of **2c**.

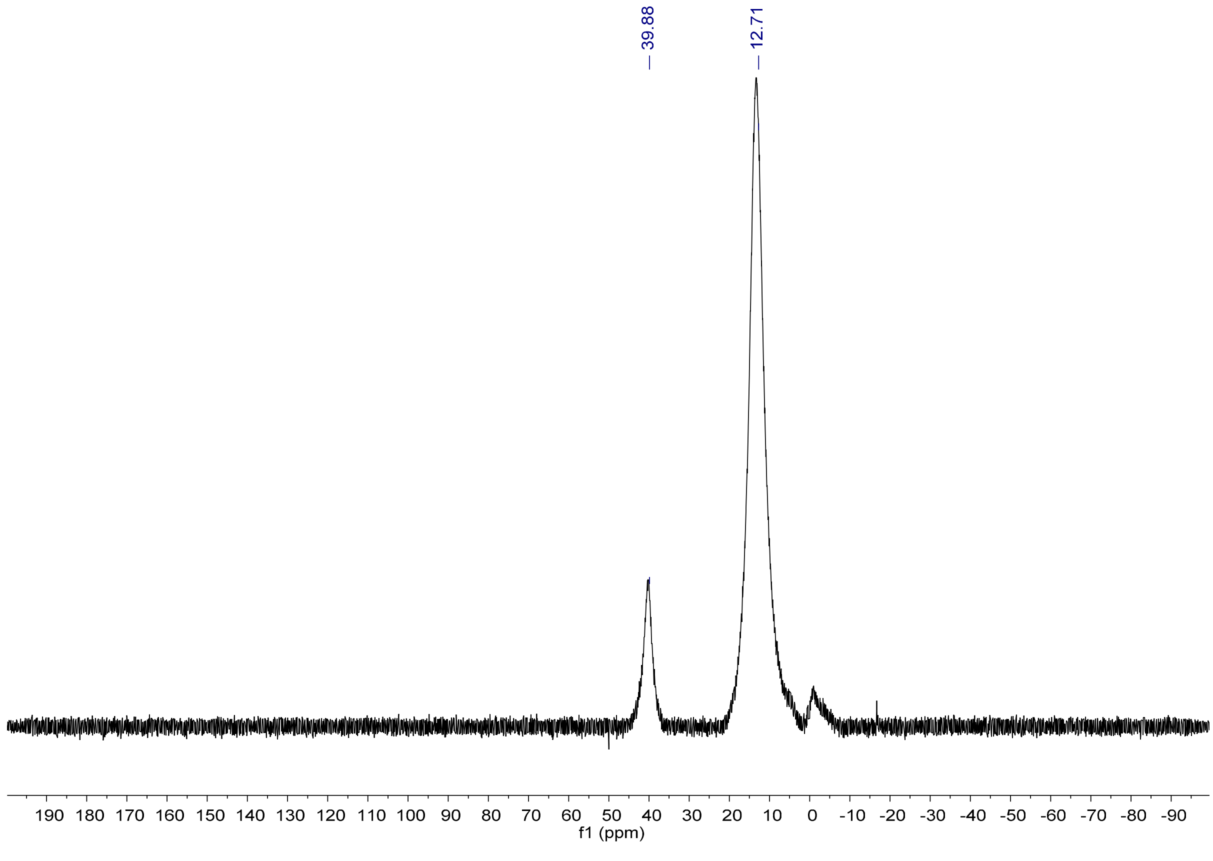

In situ ^{11}B-NMR (160 MHz, $CDCl_3$, 298 K, 0.1 M) spectrum of **2c**.

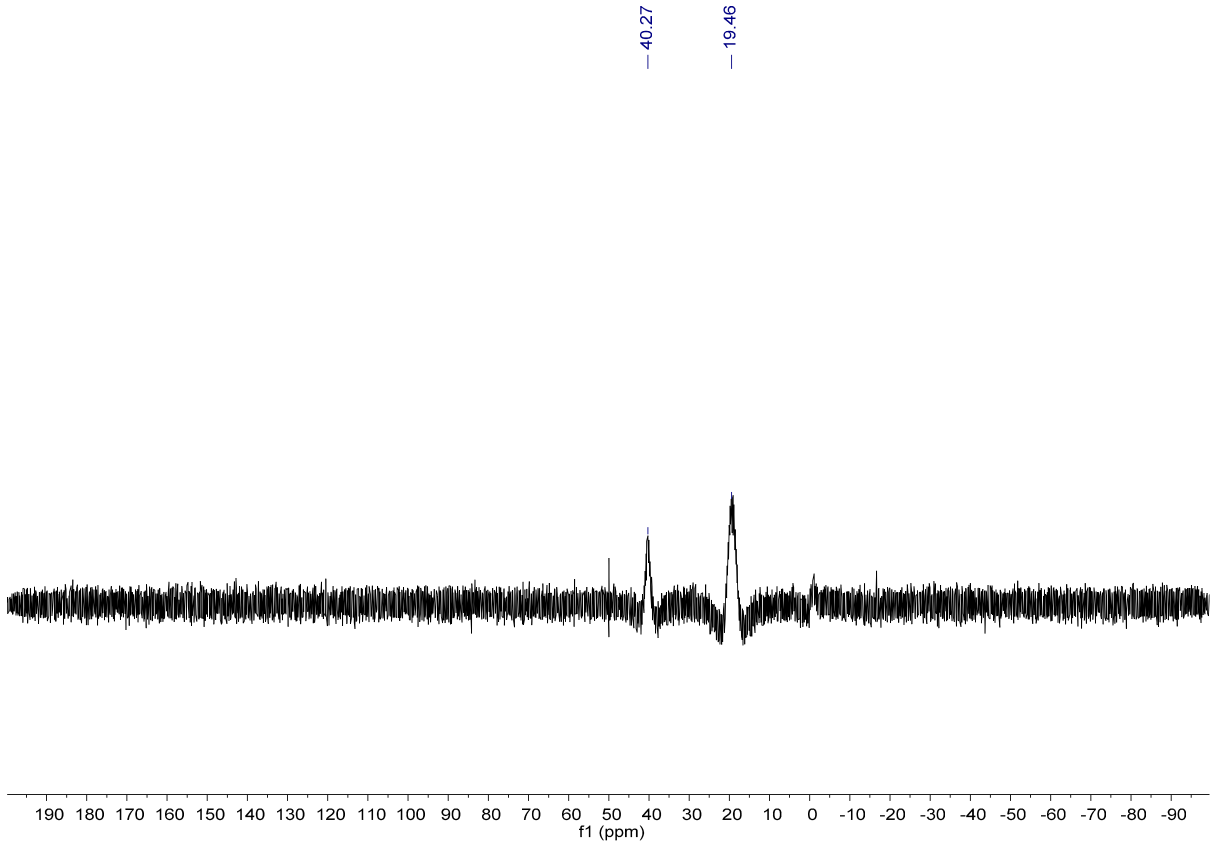

In situ ^{11}B-NMR (160 MHz, $CDCl_3$, 298 K, 0.04 M) spectrum of **2c**.

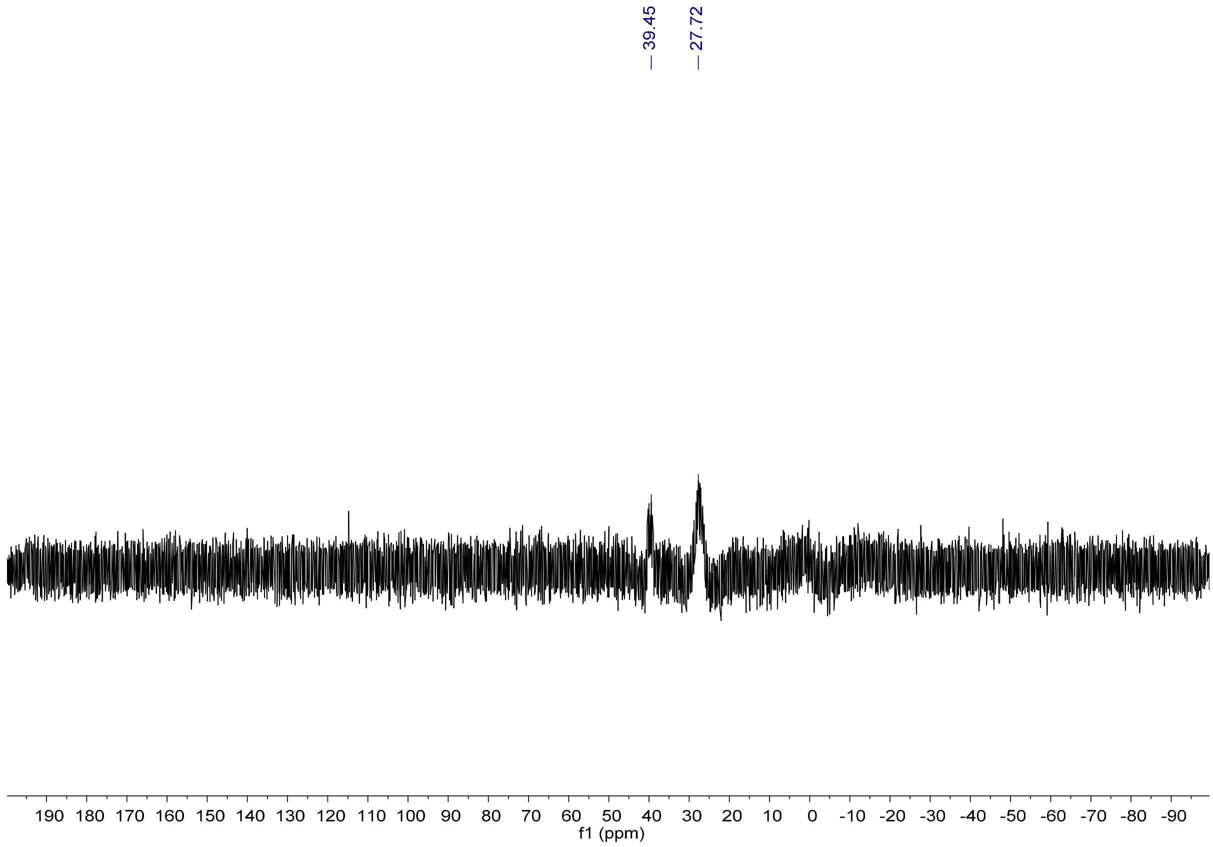

In situ ^{11}B-NMR (160 MHz, $CDCl_3$, 298 K) spectrum of **2c** across concentrations 0.04–0.4 M.

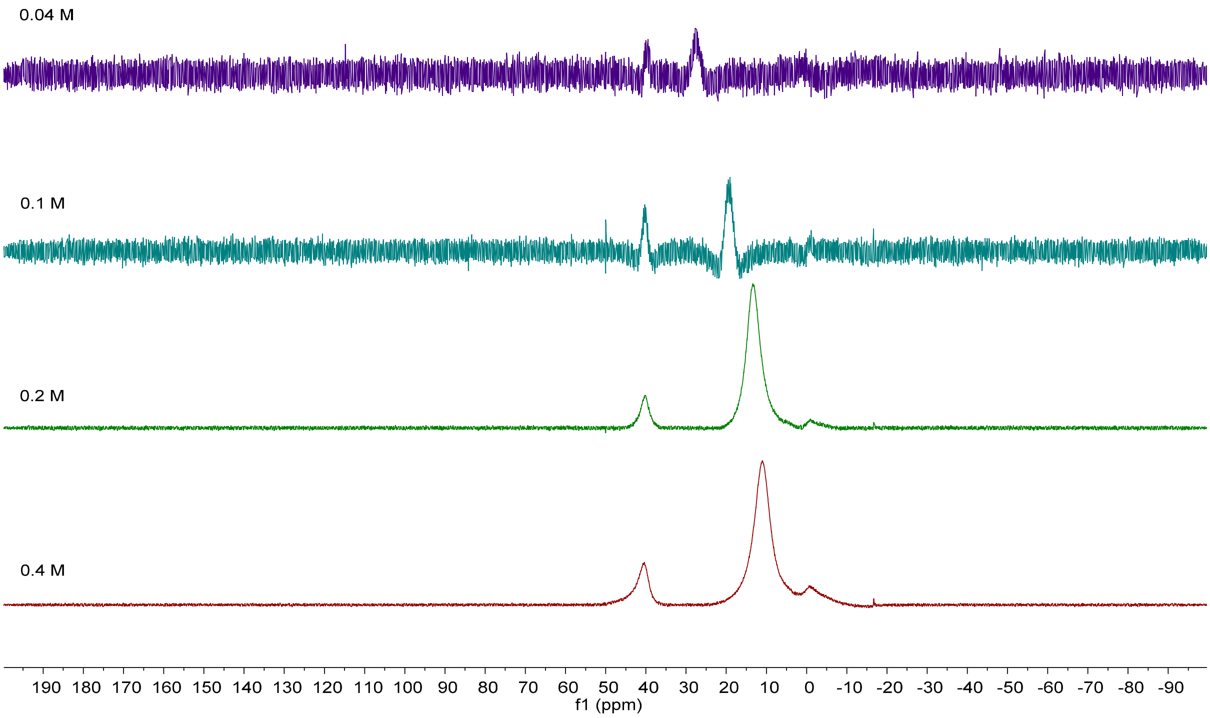

^{19}F-NMR (283 MHz, $CDCl_3$, 298 K) spectrum of **2c** crystals.

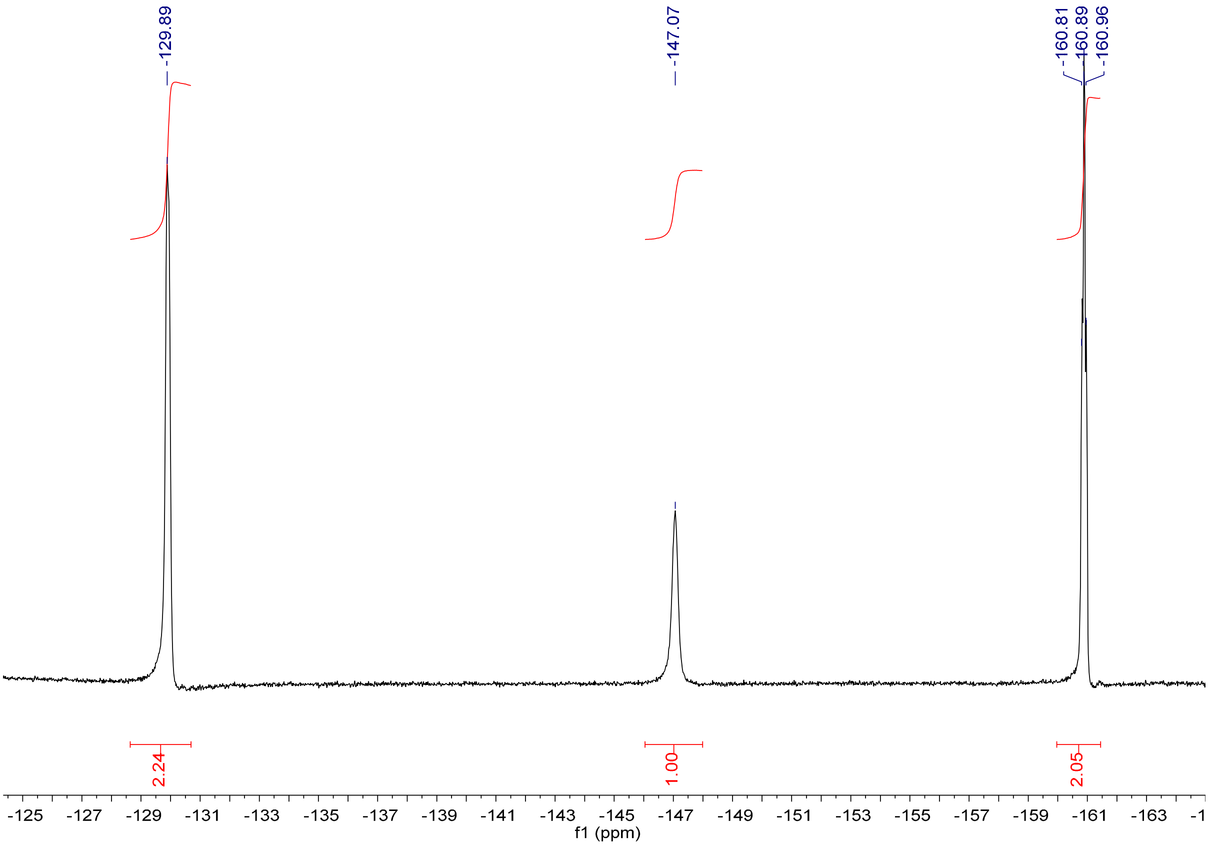

In situ ^{19}F-NMR (283 MHz, $CDCl_3$, 298 K, 0.4 M) spectrum of **2c**.

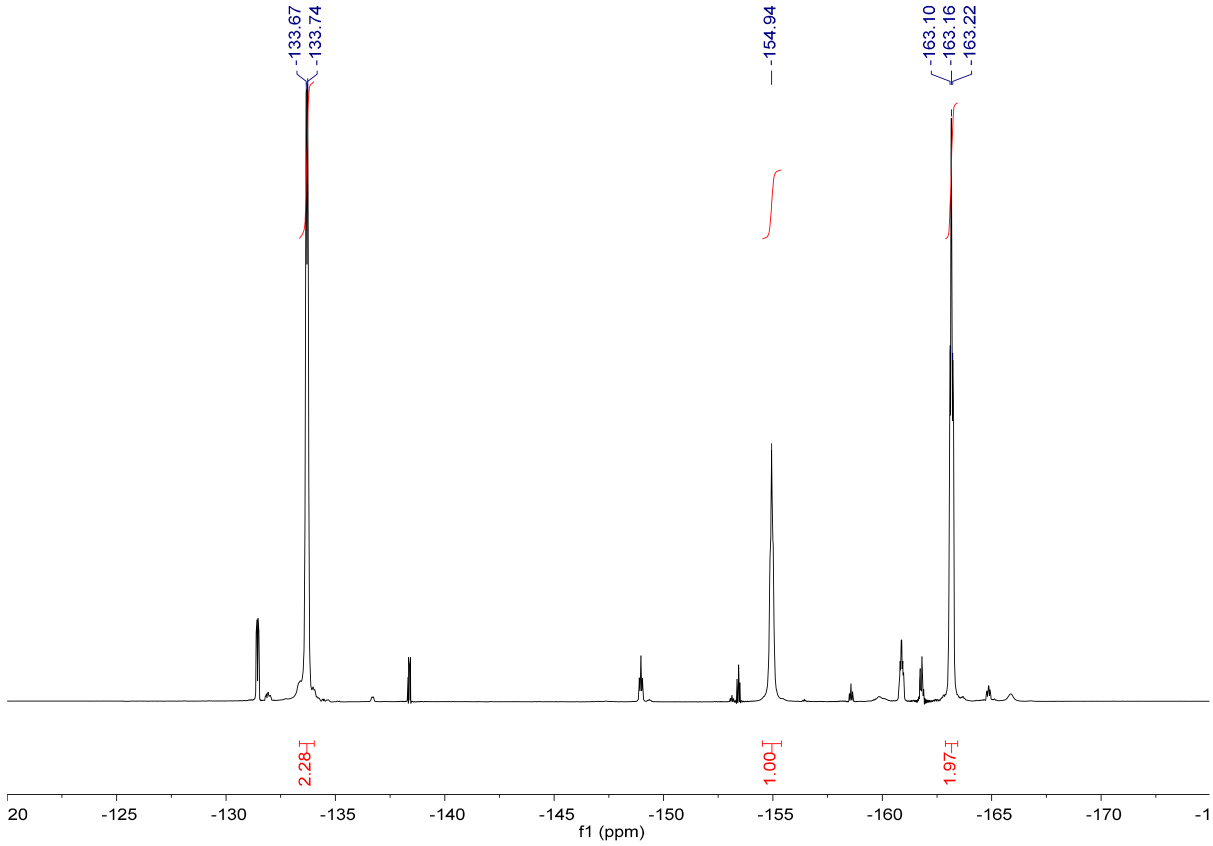

In situ ^{19}F-NMR (283 MHz, $CDCl_3$, 298 K, 0.2 M) spectrum of **2c**.

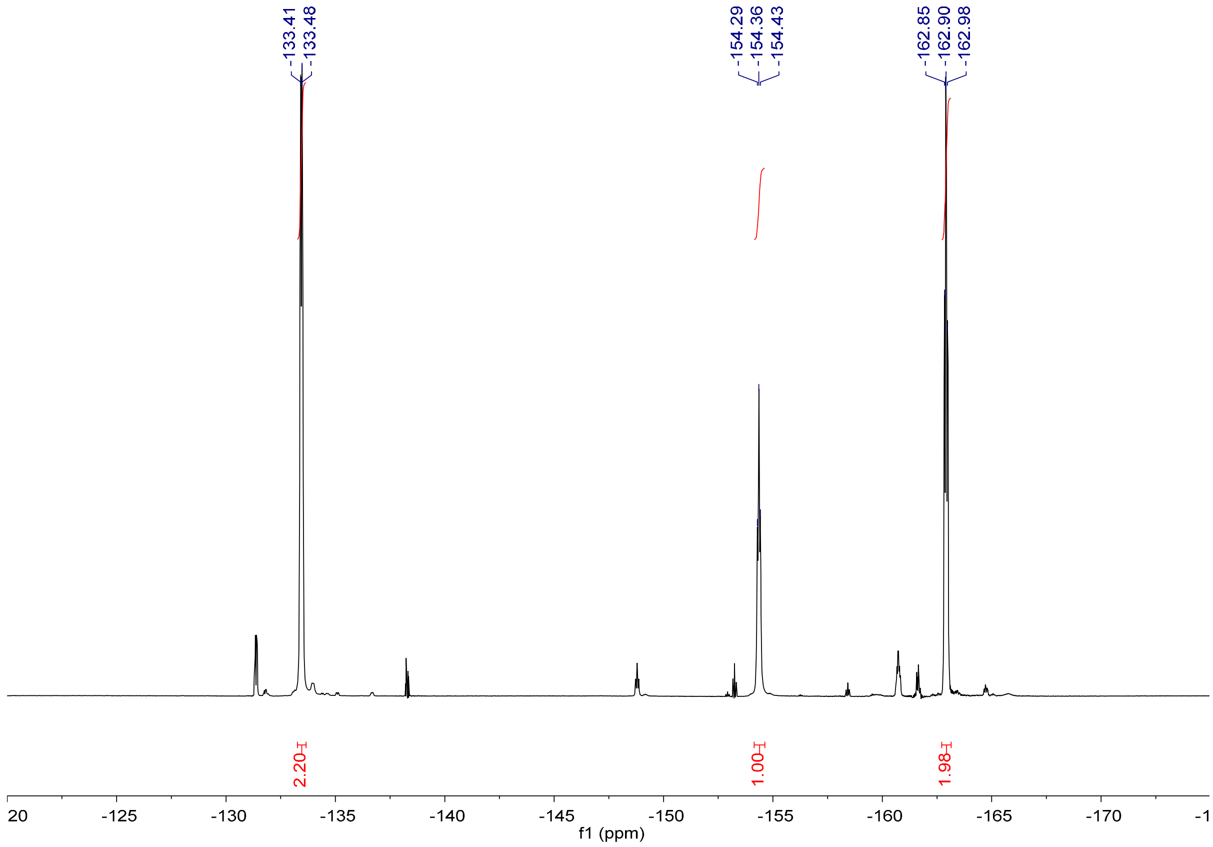

In situ ^{19}F-NMR (283 MHz, $CDCl_3$, 298 K, 0.1 M) spectrum of **2c**.

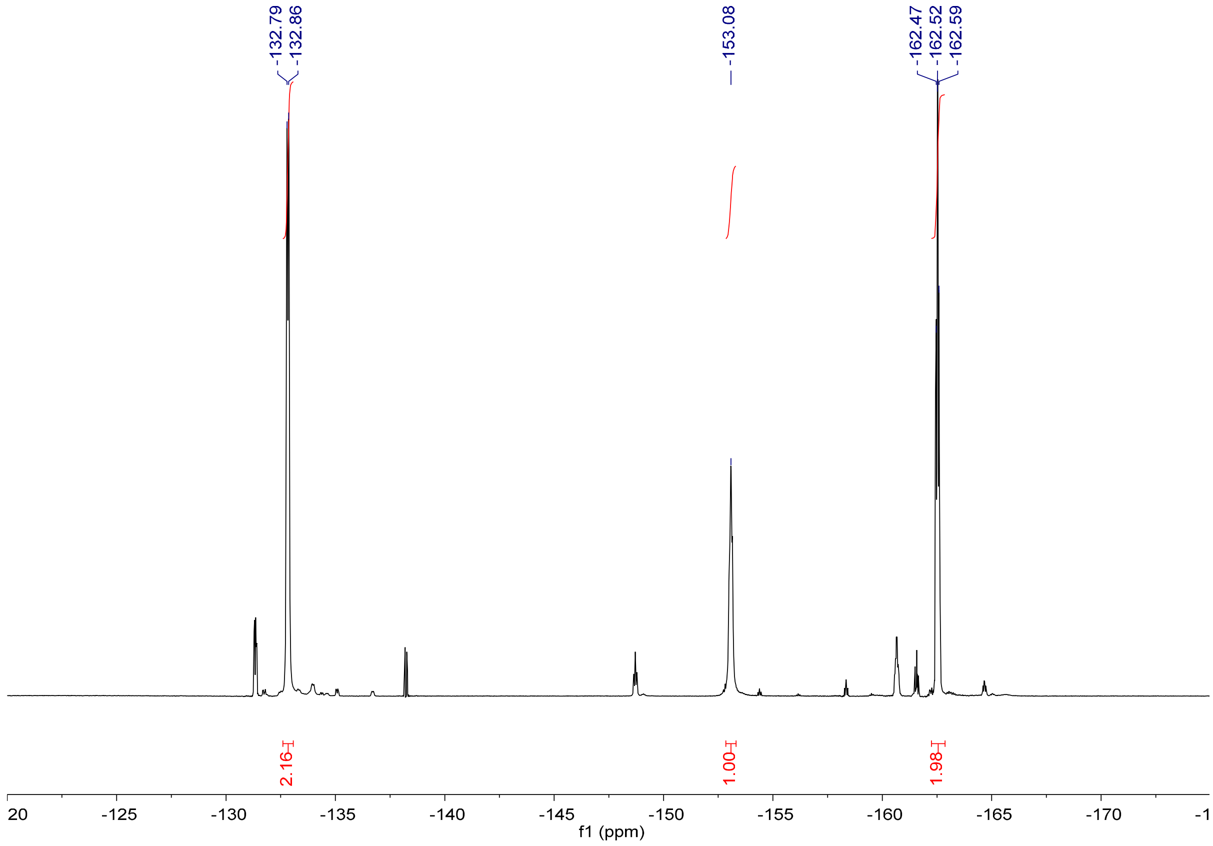

In situ ^{19}F-NMR (283 MHz, $CDCl_3$, 298 K, 0.04 M) spectrum of **2c**.

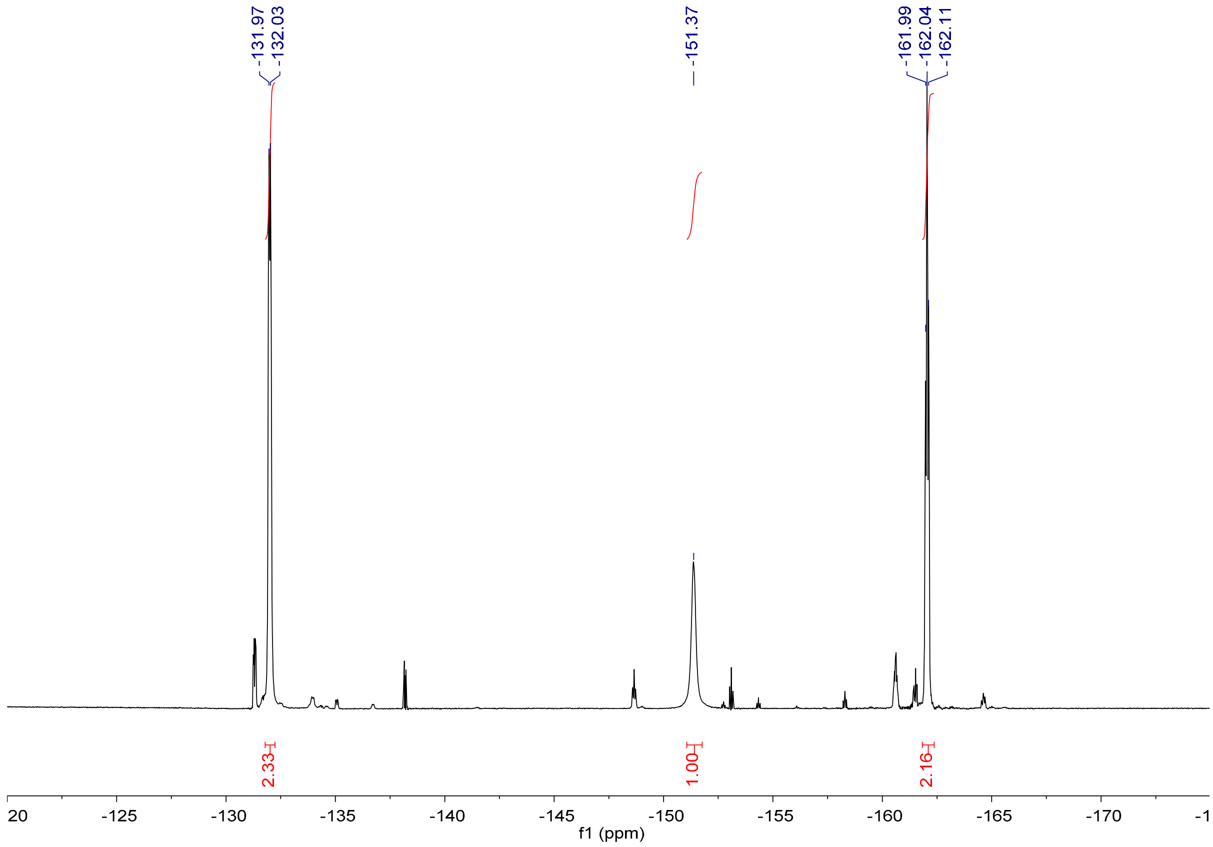

In situ ^{19}F-NMR (283 MHz, $CDCl_3$, 298 K) spectra of **2c** across concentrations 0.04–0.4 M

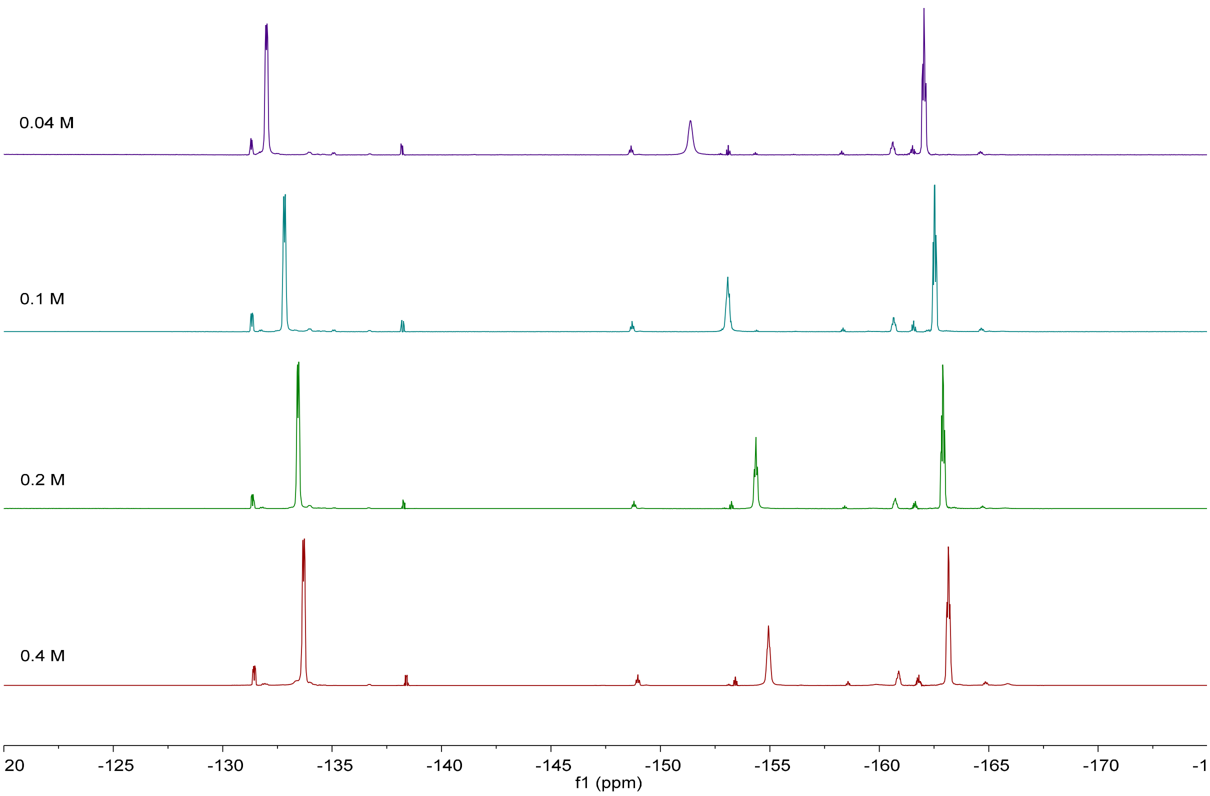

In situ ^{1}H-NMR (500 MHz, $CDCl_3$, 298K) spectrum of excess $B(C_6F_5)_3$.

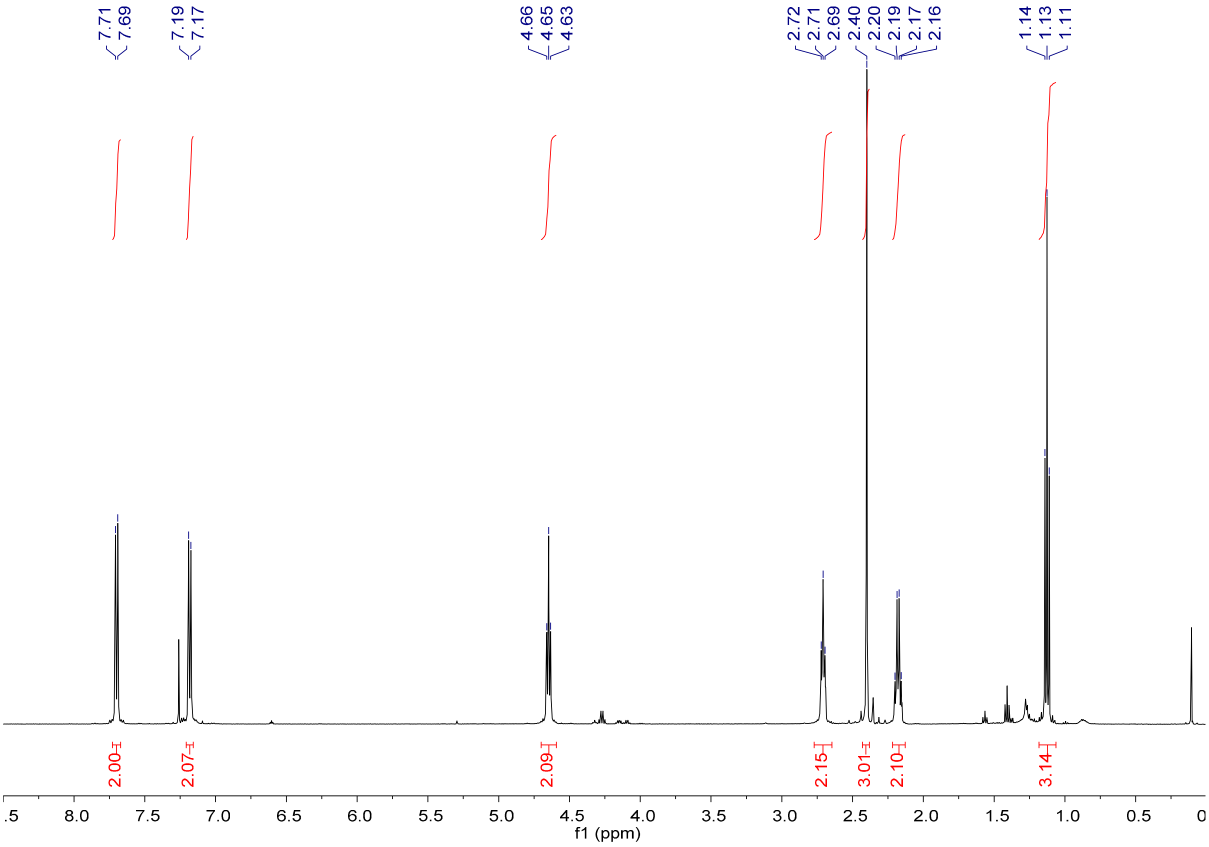

In situ ^{11}B-NMR (160 MHz, $CDCl_3$, 298 K) spectrum of excess $B(C_6F_5)_3$.

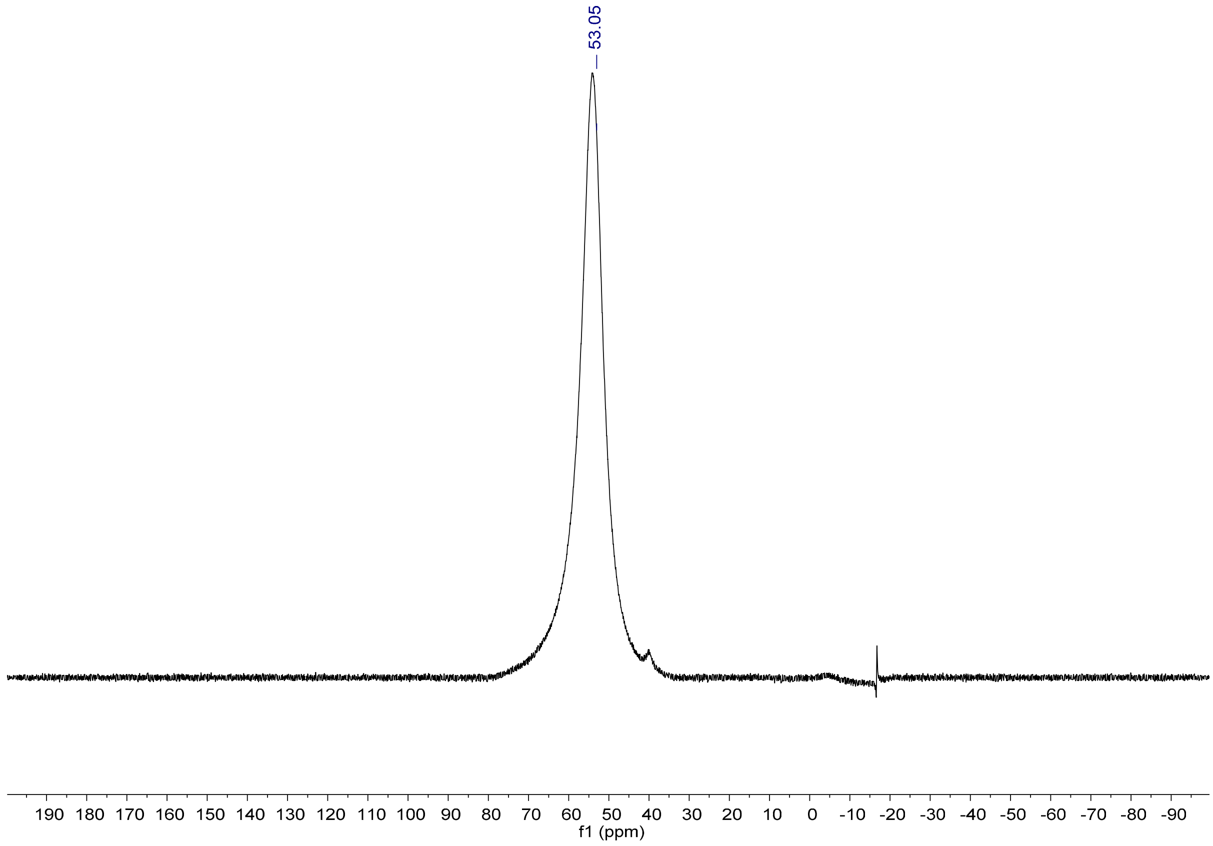

In situ ^{19}F-NMR (283 MHz, $CDCl_3$, 298 K) spectrum of excess $B(C_6F_5)_3$.

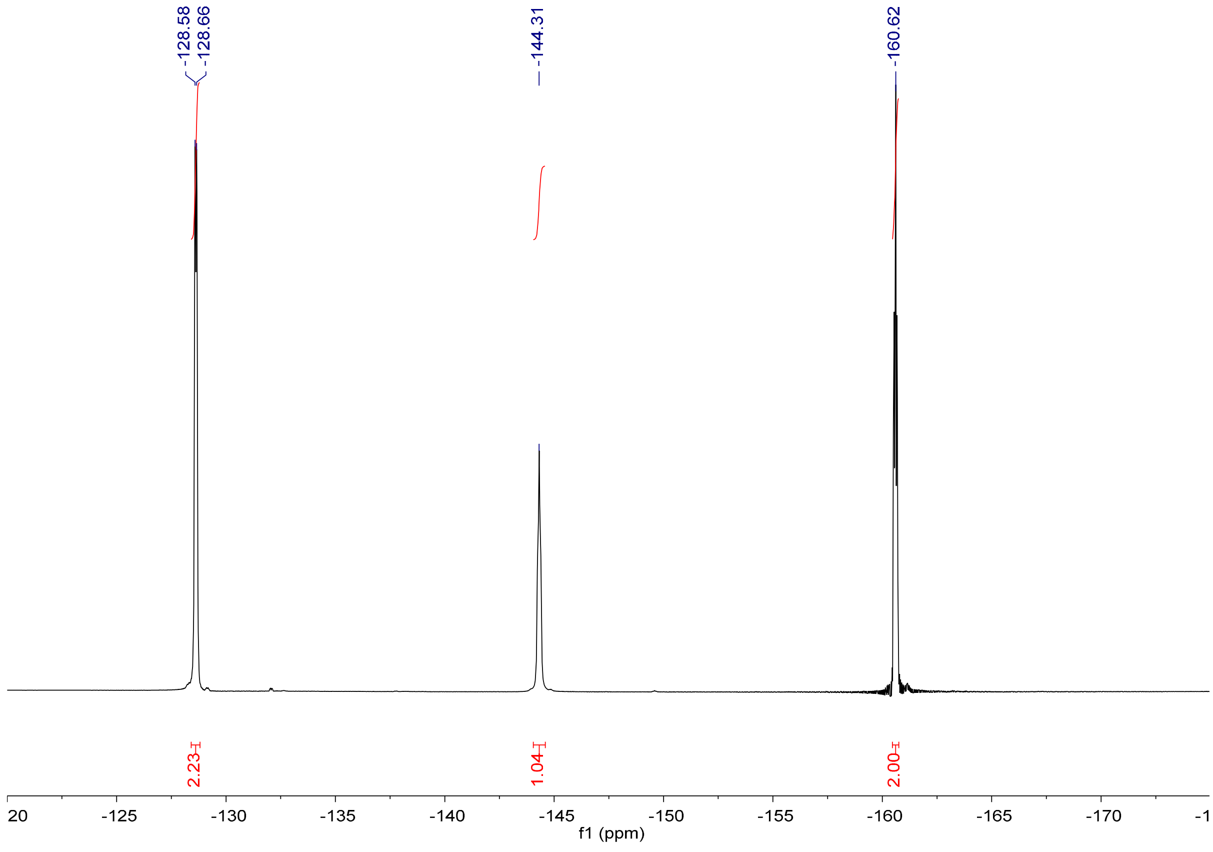

In situ ^{1}H-NMR (500 MHz, $CDCl_3$, 298K) spectrum of excess **2b**.

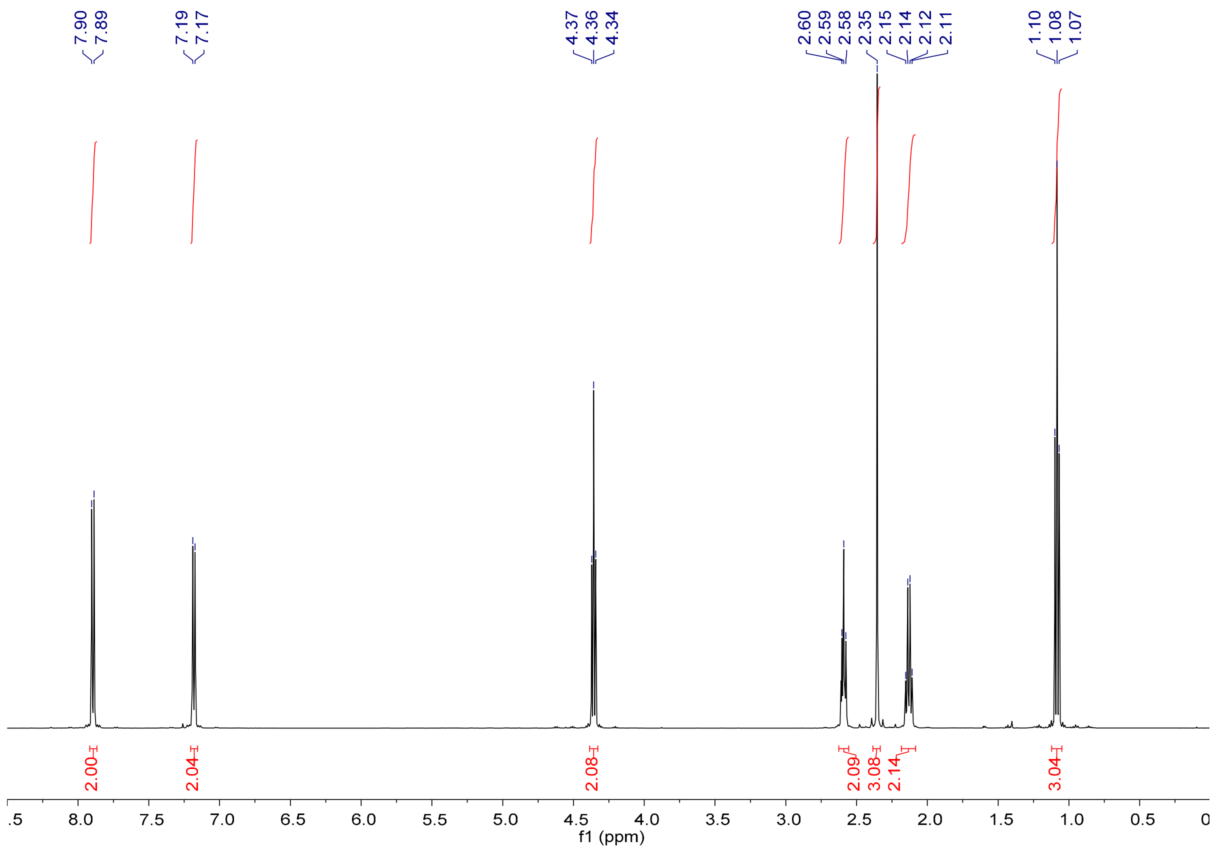

In situ ^{11}B-NMR (160 MHz, $CDCl_3$, 298 K) spectrum of excess **2b**.

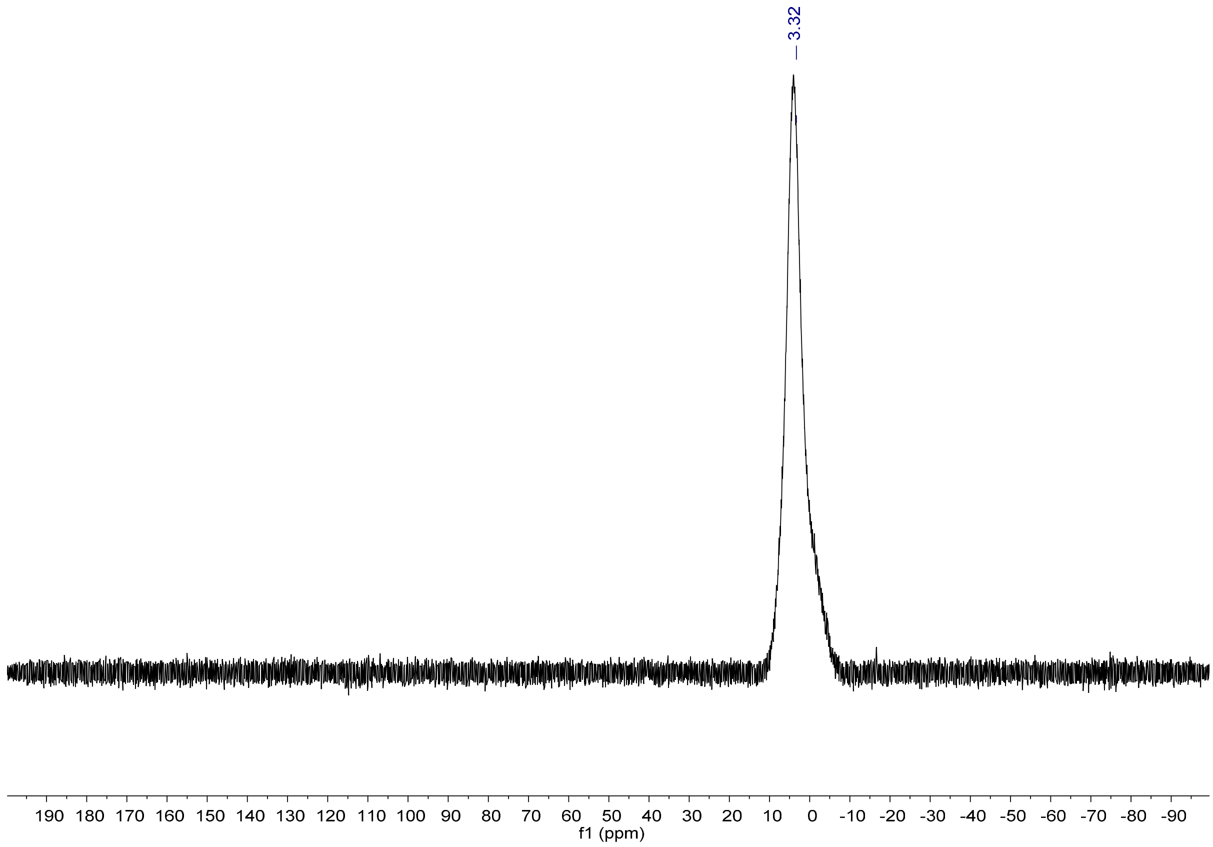

In situ ^{19}F-NMR (283 MHz, $CDCl_3$, 298 K) spectrum of excess **2b**.

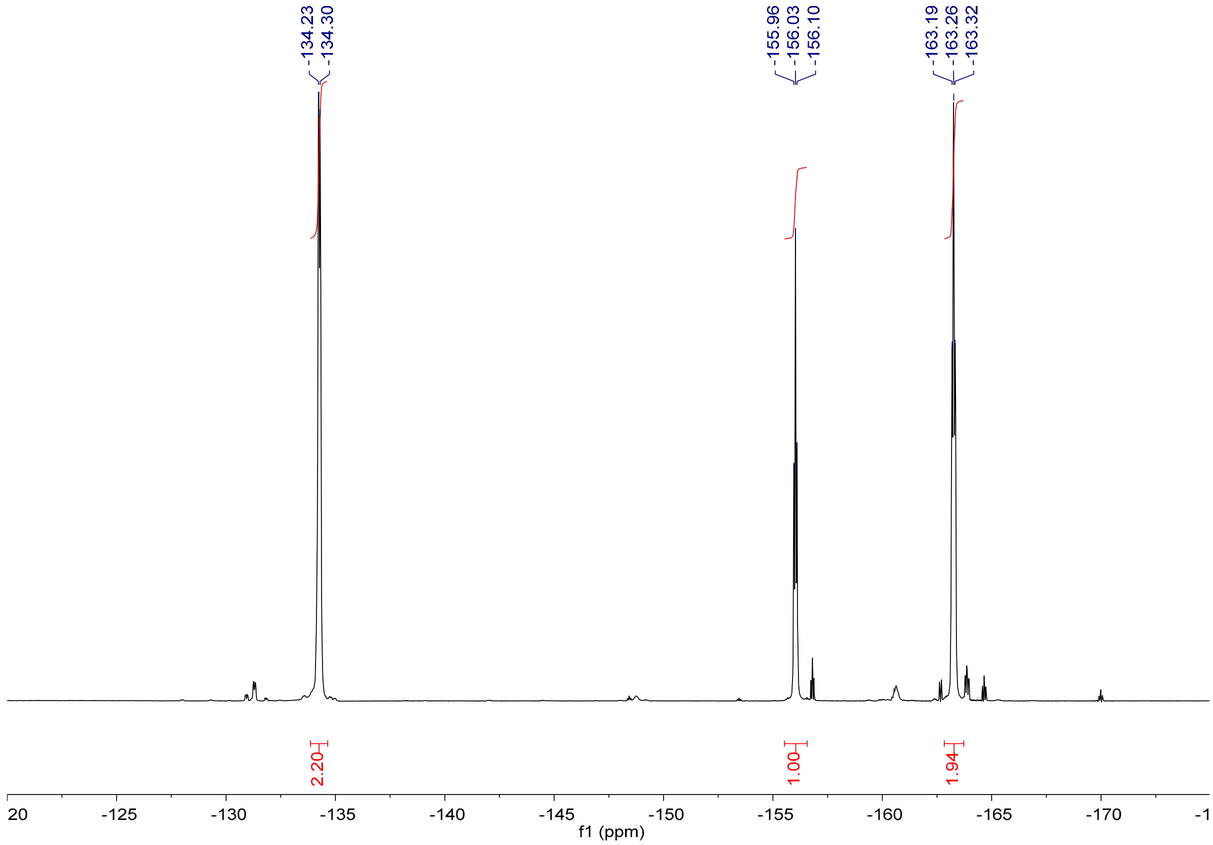

Supplement: Supplementary file 1 [file molecules-20-04530-s001.pdf]
